# Identification of Rocaglate Acyl Sulfamides as Selective Inhibitors of Glioblastoma Stem Cells

**DOI:** 10.1021/acscentsci.4c01073

**Published:** 2024-08-08

**Authors:** Zihao Wang, Ritesh P. Thakare, Shalaka Chitale, Alok K. Mishra, Stanley I. Goldstein, Alice C. Fan, Rui Li, Lihua Julie Zhu, Lauren E. Brown, Regina Cencic, Sidong Huang, Michael R. Green, Jerry Pelletier, Sunil K. Malonia, John A. Porco

**Affiliations:** ∇ Department of Chemistry and Center for Molecular Discovery (BU-CMD), Boston University, 590 Commonwealth Avenue, Boston, Massachusetts 02215, United States; ○ Department of Molecular, Cell and Cancer Biology, University of Massachusetts Chan Medical School, Worcester, Massachusetts 01605, United States; ◆ Boston University Target Discovery Laboratory (BU-TDL), Boston, Massachusetts 02215, United States; ¶ Department of Pharmacology, Physiology, and Biophysics, Boston University, Boston, Massachusetts 02118, United States; ⊥ Department of Molecular Medicine and Program in Bioinformatics and Integrative Biology, University of Massachusetts Chan Medical School, Worcester, Massachusetts 01605, United States; # Department of Biochemistry, McGill University, Montreal, QC H3G 1Y6, Canada

## Abstract

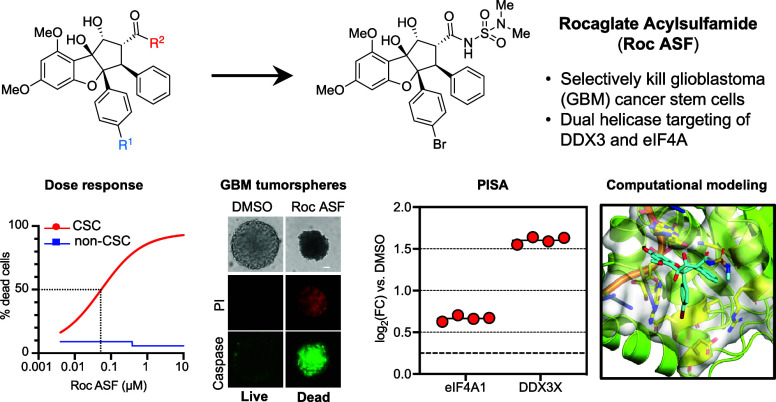

Glioblastoma (GBM) is the most aggressive and frequently occurring type of malignant brain tumor in adults. The initiation, progression, and recurrence of malignant tumors are known to be driven by a small subpopulation of cells known as tumor-initiating cells or cancer stem cells (CSCs). GBM CSCs play a pivotal role in orchestrating drug resistance and tumor relapse. As a prospective avenue for GBM intervention, the targeted suppression of GBM CSCs holds considerable promise. In this study, we found that rocaglates, compounds which are known to inhibit translation *via* targeting of the DEAD-box helicase eIF4A, exert a robust, dose-dependent cytotoxic impact on GBM CSCs with minimal killing of nonstem GBM cells. Subsequent optimization identified novel rocaglate derivatives (rocaglate acyl sulfamides or Roc ASFs) that selectively inhibit GBM CSCs with nanomolar EC_50_ values. Furthermore, comparative evaluation of a lead CSC-optimized Roc ASF across diverse mechanistic and target profiling assays revealed suppressed translation inhibition relative to that of other CSC-selective rocaglates, with enhanced targeting of the DEAD-box helicase DDX3X, a recently identified secondary target of rocaglates. Overall, these findings suggest a promising therapeutic strategy for targeting GBM CSCs.

## Abstract 2

Rocaglate acyl sulfamides (Roc ASFs) are potent inhibitors of glioblastoma cancer stem cells (GBM CSCs). They act by targeting RNA helicases, with DDX3X and eIF4A identified as predominant targets.

## Introduction

Metastatic spread and development of therapeutic resistance pose major challenges for the treatment of cancer.^1,2^ A large body of evidence suggests that tumor initiation, progression, metastasis, and recurrence are driven by a small subpopulation (1–5%) of cells within tumors called tumor-initiating cells or cancer stem cells (CSCs). CSCs are slow-dividing, undifferentiated, self-renewing cells that give rise to the differentiated cells comprising the bulk of the tumor (nonstem cancer cells, hereafter termed “non-CSCs”). CSCs have been identified in various types of tumors such as leukemia, breast, brain, colon, and lung, although the markers and driver pathways vary among tumor types.^3,4^ In addition, CSCs interact with multiple components of the tumor microenvironment and can modulate the immune response to tumors.^5^ CSCs possess a range of capabilities that confer resistance to chemo- and radiotherapies and therefore not only persist after treatment but are often actually enriched, leading to tumor recurrence.^6,7^ These capabilities include a robust DNA damage repair system, upregulated efflux pumps, activation of survival pathways, enhanced cellular plasticity, immune evasion, and the ability to adapt to hostile microenvironments.^8,9^ Additionally, CSCs can undergo phenotypic changes such as epithelial–mesenchymal transition (EMT) which further enhance their resistance to treatment.^8^ Thus, targeting of CSCs is crucial to preventing tumor recurrence and improving patient survival after chemotherapy. Of particular interest are compounds that specifically target and eliminate CSCs while minimizing the impact on non-CSCs. Such compounds are more likely to demonstrate specific efficacy against cancers rather than acting as general cytotoxic agents.^10−13^

Glioblastoma (GBM) is the most common and aggressive malignant brain tumor in adults and generally has a poor prognosis. Irrespective of treatment, which includes surgical resection, radiotherapy, and chemotherapy, almost all patients experience tumor recurrence, leading to mortality and a median survival of <15 months. Thus, targeted prevention of tumor recurrence, by specifically eradicating GBM CSCs, is a potential therapeutic strategy for glioblastoma.

**Figure 1:**
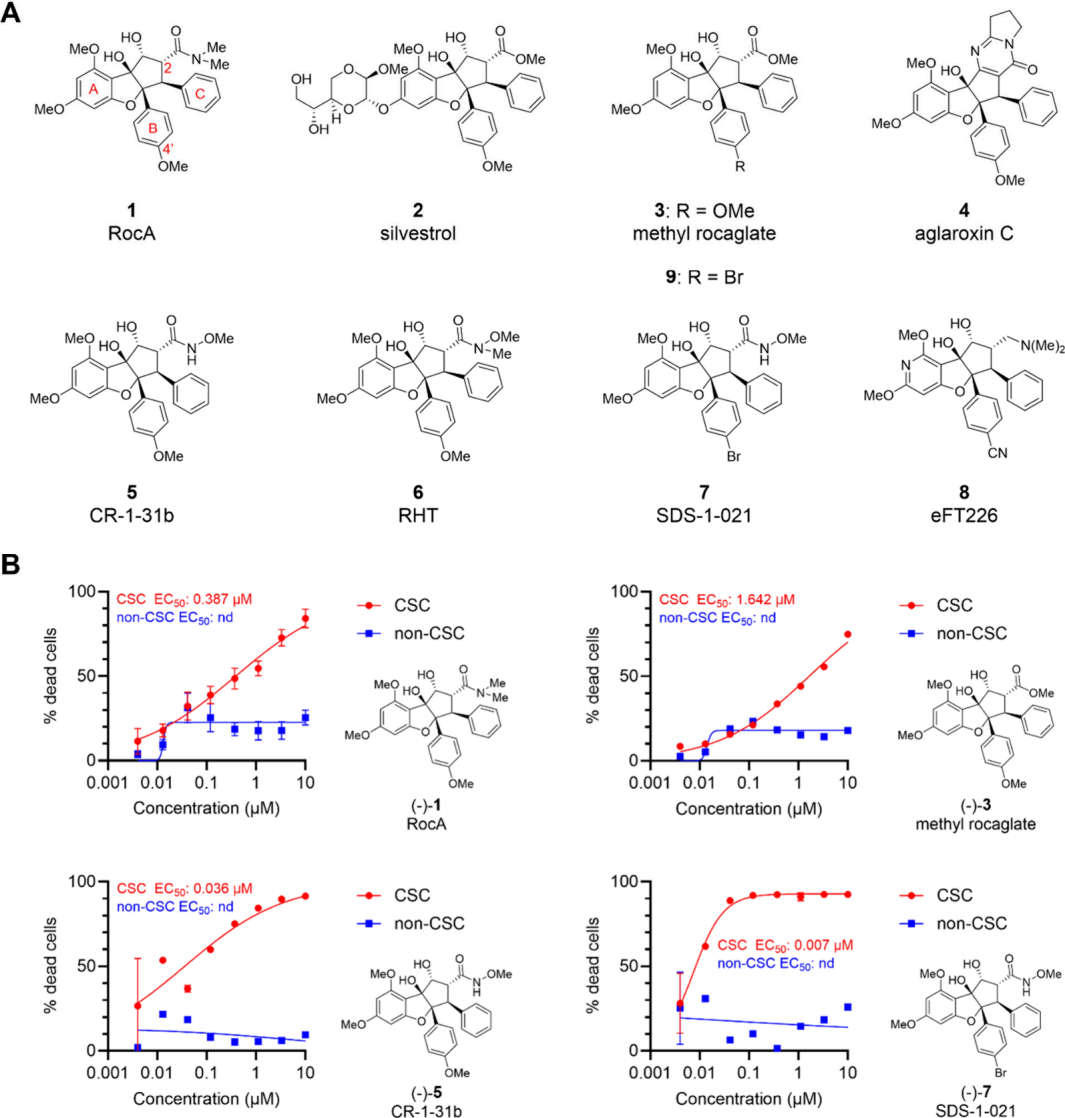
(A) Nature-produced (**1**–**4**) and synthetic (**5**–**9**) rocaglates. (**B**) Comparative dose–response for killing of CSC (red) and non-CSC (blue) populations for selected rocaglates.

Rocaglates (also known as flavaglines) are a group of natural products containing a cyclopenta[*b*]tetrahydrobenzofuran skeleton originally isolated from plants of the genus *Aglaia*.^14^ Since the first report of rocaglamide A (RocA, **1**) (Figure 1A) as an antitumor agent, there have been extensive biological studies on rocaglates.^15^ Beyond RocA, other nature-produced rocaglates (Figure 1A) include silvestrol (**2**), methyl rocaglate/aglafoline (**3**), and aglaroxin C (**4**). In addition, many synthetic rocaglates have been developed as molecular probes and drug candidates, including the *C2*-hydroxamates **CR-1-31b** (**5**), rohinitib (RHT, **6**), and **SDS-1-021** (**7**) as well as the *C2*-amine congener eFT226 (zotatifin, **8**), a compound currently in clinical development for breast and nonsmall cell lung cancers. In a comprehensive study of >200 natural and synthetic rocaglates, Pelletier and co-workers showed that most rocaglates preferentially repress translation of mRNAs containing purine-rich 5′ leaders by stimulating the binding of DEAD-box helicase eIF4A to these sequences and in some cases also exerting a *trans*-inhibitory effect on global translation by limiting the pool of eIF4A (and parent complex eIF4F) available for ribosome recruitment.^16^ Mechanistically, rocaglates bind a bimolecular cavity formed by the complexation of eIF4A onto polypurine RNA, as shown in an X-ray cocrystal structure of a RocA:eIF4A1:r(AG)_5_ complex reported by Iwasaki and co-workers.^17^ The same group found that RocA could additionally clamp the related DEAD-box helicase DDX3 to polypurine RNA in an ATP-independent manner, thereby expanding our understanding of the potential mechanisms underlying RocA’s antiproliferative effects.^18^

In this study, we sought to probe the activity of rocaglates against GBM CSCs. Using comparative dose–response assays, we found that rocaglate translation inhibitors exhibit potent, dose-dependent cytotoxic effects against GBM CSCs at concentrations that are predominantly nonlethal to non-CSC populations, prompting further study of this chemotype and the underlying mechanism. Herein we describe our results, including the identification of new rocaglate congeners for use as tool compounds to explore the mechanism of action for targeted and selective killing of GBM CSCs.

## Results and Discussion

### Rocaglates Exhibit Selective, Dose-Dependent Killing of CSCs in a Patient-Derived Glioblastoma Cell Line

To initiate our study, we first evaluated a cohort of seven rocaglate translation inhibitors (**1**, **3**–**7**, and **9**, Figure 1 and Table 1; compounds **2** and **8** were not tested due to unavailability). To assay for compounds that selectively affect GBM CSCs, we employed the patient-derived tumor-initiating cell (TIC) cell line 0308^19^ that forms neurospheres (consisting exclusively of tumor-initiating cells or CSCs) when cultured in neurobasal (NBE) medium. Upon exposure to serum and growth factor withdrawal, however, the cell line differentiates, acquires a flattened morphology, and forms a non-CSC population.^20^ Thus, compounds can be tested in parallel to assess their effect on GBM CSCs versus GBM non-CSCs as separate populations with a clean and robust response. This approach has been previously used to perform RNAi-based genetic screens to identify modulators of CSCs.^20^ In our study, GBM0308 cells, cultured in stem cell conditions to form CSCs or in medium containing serum to induce differentiation into a non-CSC population, were treated in parallel with compounds at varying doses. Three days post-treatment, propidium iodide (PI) and Hoechst staining were performed and quantified using a Celigo image cytometer. Hoechst dye stained all live nucleated cells, while PI exclusively stained dead cells. Cell viability and percentage of cell death were then determined.

**Table 1:**
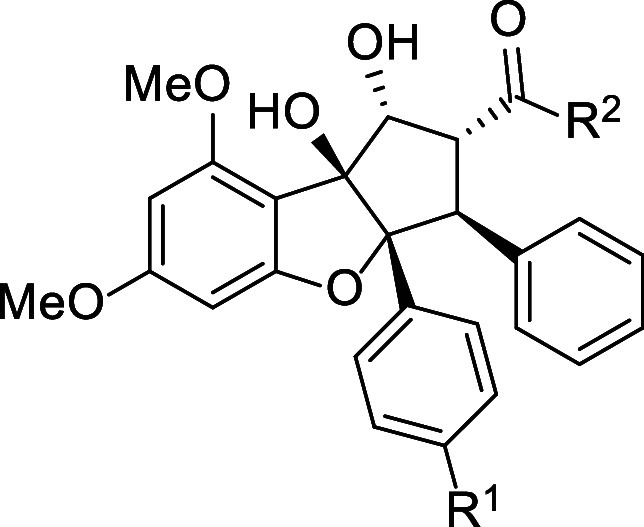
Comparative Activity of Key Rocaglates against GBM CSCs and Non-CSCs

			CSCs		Non-CSCs	
compound	R^1^	R^2^	EC_50_ (μM)^b^	max. observed efficacy (% dead cells)	EC_50_ (μM)^b^	max. observed efficacy (% dead cells)
(−)-**1**	OMe	N(Me)_2_	0.387	84	nd	31
(−)-**3**	OMe	OMe	1.642	75	nd	23
*rac*-**4**^a^	–	–	nd	42	nd	26
(−)-**5**	OMe	NH(OMe)	0.036	91	nd	22
*rac*-**6**	OMe	N(Me)OMe	0.922	81	nd	25
(−)-**7**	Br	NH(OMe)	0.007	92	nd	31
(−)-**9**	Br	OMe	0.151	88	nd	25

a See Figure 1A for chemical structure.

b For each cell type, EC_50_ values are provided for compounds causing at least 50% cell death. Values shown are relative EC_50_ values obtained from a variable-slope, four-parameter nonlinear regression constrained to bottom = 0% and top <100% cell death.

Table 1 and Figure 1 summarize our results for these experiments. Interestingly, all seven rocaglates showed specific cytotoxic activity against CSCs with lesser impact on non-CSCs (*cf.*Figures 1 and S1 for dose–response curves). This selectivity manifests as a striking difference in the maximum observed percent death of the respective cell populations; in CSCs, several compounds showed dose-dependent cytotoxicity that plateaued at ≥75% cell death, whereas their dose-dependent cytotoxicity against non-CSCs, when observed, plateaued at ≤31% of the cells. A notable exception was the rocaglate pyrimidinone (RP) aglaroxin C (**4**) (Figure 1A) which failed to surpass >50% cell death in CSCs (Table 1 and Figure S1).

While most compounds showed CSC selectivity, there was a wide variance in their anti-CSC potency, with CSC EC_50_ values ranging from ∼1.6 μM to 7 nM (Table 1). We also noted nascent structure–activity relationships (SARs) among the set. First, two of the more potent compounds ((−)-**7** and (−)-**9**) bore a bromine at the B-ring C4′, a site that is methoxy-substituted in most rocaglates found in nature. A head-to-head comparison showed that the *C4′*-brominated congeners **7** and **9** each exhibited a 5- to 10-fold improvement in potency over their *C4′*-methoxy congeners **5** and **3**, respectively. In addition, the *C2*-hydroxamic esters (**5**, **7**) also outperformed their *C2*-methyl ester counterparts **3** and **9**, respectively, by >20-fold, in addition to outperforming the *C2*-dimethyl amide RocA (**1**) and *N*-methyl hydroxamic ester RHT (**6**). This preliminary SAR suggested that a protic N–H at C2 as well as a bromine at C4′ were key potency drivers. Given the potential for hydroxamates such as **5** and **7** to ionize at physiological pH (p*K*_a_ range 6–10), we postulated that the N–H hydroxamic esters may behave as carboxylic acid surrogates.^21^ To further probe this hypothesis, we sought to synthesize additional analogs bearing both carboxylic acids and ionizable acid isosteres at C2 while also continuing to probe the impact of bromination at C4′.

### Synthesis of N-Acylated Derivatives of Rocaglaic Acids

After identification of *O*-methyl hydroxamic esters **5** and **7** as the most potent and selective inhibitors of GBM CSCs, we targeted direct replacement of the *C2*-hydroxamic ester with carboxylic acids and acid bioisosteres such as *N*-acyl sulfamides and sulfonamides, whose p*K*_a_ values generally fall within the range for carboxylic acids (4–5).^21^ Employing our previously established method for excited-state intramolecular proton transfer (ESIPT)-mediated [3 + 2] photocycloaddition to produce rocaglates,^22^ we subsequently transformed the derived rocaglaic acids **10** and **11** (obtained by hydrolysis of **3** and **9**) to the rocaglate β-lactones **12** and **13** by treatment with *bis*(2-oxo-3-oxazolidinyl)phosphinic chloride (BOP-Cl)/triethylamine.^23^

**Scheme 1:**
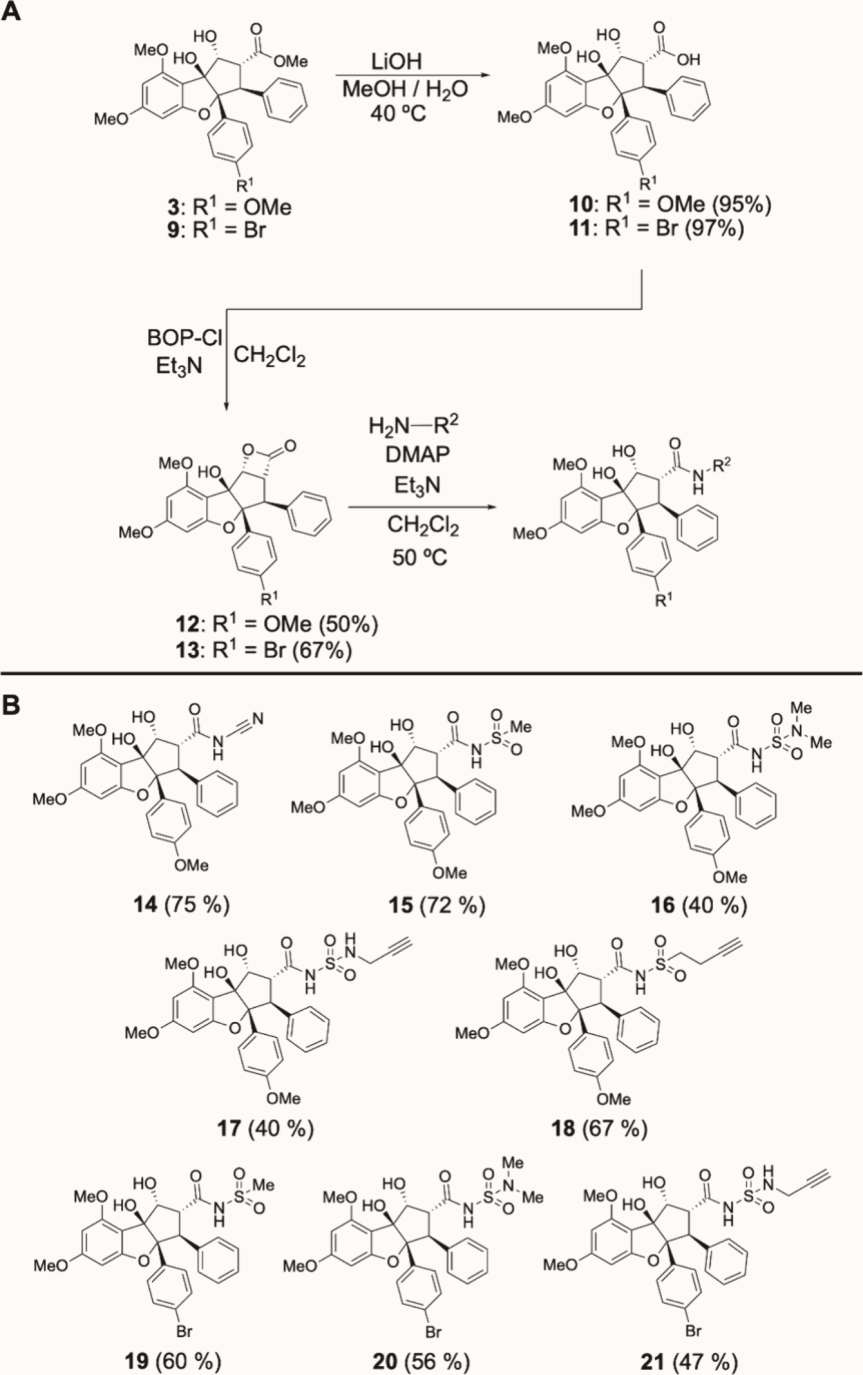
(A) Synthesis of Rocaglaic Acids and Derived *N*-Acyl Derivatives; (B) Substrate Scope for β-Lactone Ring Opening (One-Step Yields from Precursors Are Denoted in Parentheses)

We next used β-lactones **12** and **13** as lynchpin substrates to generate *N*-acylated congeners through β-lactone ring opening with various nitrogen nucleophiles (*e.g.*, cyanamide, sulfonamide, and sulfamide; Scheme 1). Ring opening of **12** with cyanamide as a nucleophile afforded the corresponding rocaglate acyl cyanamide **14** in 75% yield. When methanesulfonamide was used in ring opening with **12**, a 72% yield of rocaglate acyl sulfonamide **15** was obtained. However, use of *N*,*N*-dimethylsulfamide as the nucleophile with **12** afforded a lower yield (40%) of rocaglate acyl sulfamide **16**. We also used alkynylated reagents such as *N*-propargyl sulfamide and 3-butyne-1-sulfonamide for ring opening of **12** to produce alkyne-tagged congeners such as **17** and **18**, respectively (*vide infra*). Additionally, we prepared the 4′-brominated congeners **19**–**21** using ring openings of β-lactone substrate **13**. We note that using these weak nitrogen nucleophiles, stoichiometric DMAP and mild heating (50 °C) were required for successful β-lactone ring opening. Conventionally, carboxylic acids such as **10** and **11** can be converted to amides by reaction with amines in the presence of excess *N*,*N*′-dicyclohexylcarbodiimide (DCC), but the *N*,*N*′-dicyclohexylurea byproduct generated is often difficult to remove entirely *via* column chromatography.^24^ β-Lactone ring opening of **12** and **13** offers an alternative method to generate *N*-acylated rocaglate derivatives using mild conditions and comparatively facile purification.

**Scheme 2:**
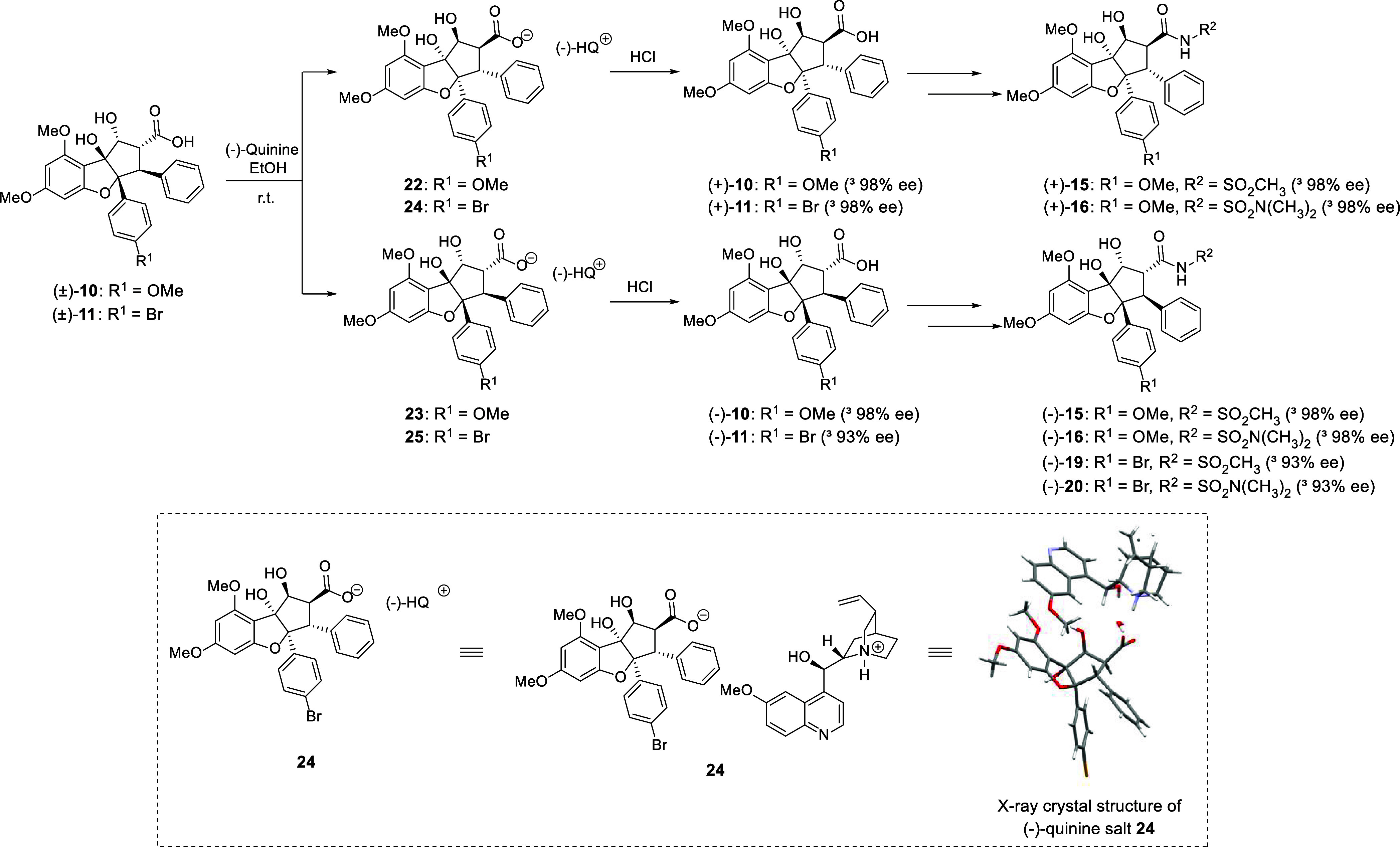
Chiral Resolution of (+)- and (−)-Rocaglaic Acids; The Absolute Stereochemistry of the Derived Salt **24** Was Confirmed by X-ray Crystallography (Inset)

While initial derivatization studies employed (±)-**12** and (±)-**13** (thus producing chiral, racemic congeners), we also sought to access key analogs in enantioenriched form. We have previously reported access to enantiomerically enriched rocaglates through reductive kinetic resolution of an aglain ketone precursor, several steps upstream from our ester starting materials **3** and **9**.^25^ Toward an alternative, scalable method for separation of chiral, racemic rocaglaic acids at a later stage, we evaluated direct resolution of rocaglaic acids using chiral bases *via* formation of diastereomeric salts.^26^ In these experiments, we found that (±)-rocaglaic acid **10** could be resolved to its corresponding enantiomers after treatment with (−)-quinine. Specifically, we treated (±)-rocaglaic acid **10** with 1 equiv of (−)-quinine at 25 °C in ethanol to afford a mixture of the diastereomeric salts **22** and **23** in quantitative yield (Scheme 2). We were able to cleanly isolate **22** through recrystallization in acetone, while **23** was recovered from the mother liquor. Enantioenriched rocaglaic acids (+)-**10** and (−)-**10** were obtained after treatment of the respective salts **22** and **23** with 5% HCl (Figure S2). This process was repeated by resolving (+)-**11** and (−)-**11** from the (±)-brominated rocaglaic acid **11** (which was obtained by hydrolysis of chiral, racemic **9**) *via* diastereomeric salts **24**/**25**. Single-crystal X-ray diffraction analysis confirmed the absolute configuration (Flack parameter = 0.002) of (+)-rocaglaic acid (−)-quinine salt **24** (Scheme 2), which is the opposite enantiomer of naturally occurring rocaglates. Subsequently, the enantioenriched rocaglate acyl sulfonamides (−)-**15**/(−)-**19** as well as enantioenriched acyl sulfamides (−)-**16**/(−)-**20** were synthesized from their corresponding precursors (−)-**10**/(−)-**11**, respectively, *via* β-lactone formation and ring-opening (*cf.*Scheme 1). For comparison purposes, the enantiomeric derivatives (+)-**15** and (+)-**16** were also prepared from (+)-**10**. Our chiral resolution method using (−)-quinine offers a means to generate enantioenriched rocaglates that is more economical and scalable than our previously established methods involving kinetic resolution of aglain precursors or using TADDOL derivatives as chiral mediators.^25,27,28^

**Figure 2:**
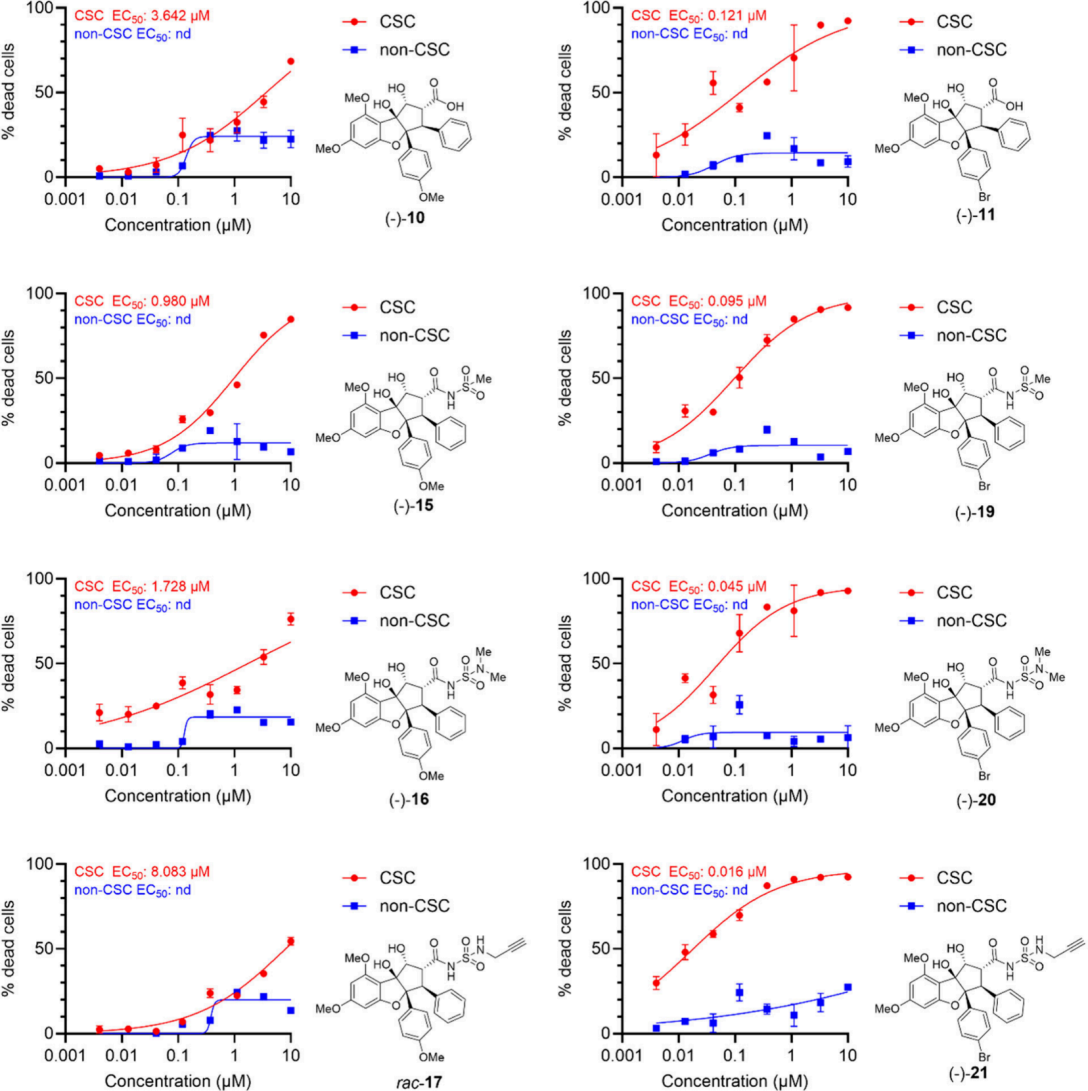
Comparative dose–response for killing of CSC (red) and non-CSC (blue) populations for selected rocaglaic acid and *N*-acylated derivatives showing enhanced selective CSC killing for *C4′*-brominated congeners (right column) over their *C4′*-methoxy-substituted counterparts (left column).

Figure 2, Table 2, and Figure S1 summarize the activity of the rocaglaic acids and *N*-acylated derivatives against GBM CSC and non-CSC populations. Consistent with the earlier cohort, CSC-selective activity (defined as CSC maximum observed cell death, ≥75%; non-CSC maximum observed cell death, <30%) was observed for nearly all compounds synthesized, except for acyl cyanamide **14**, alkynylated acyl sulfamide **17**, and alkynylated acyl sulfonamide **18**, which all showed <75% cell killing at the highest tested concentration (10 μM) against both CSCs and non-CSCs (Figures 2 and S1). Also consistent with earlier results, head-to-head comparisons revealed that the *C4′*-brominated congeners consistently showed superior potency, with compounds (−)-**11**, (−)-**19**, (−)-**20**, and (−)-**21** exhibiting CSC EC_50_ < 125 nM, while their *C4′*-methoxy counterparts (−)-**10**, (−)-**15**, (−)-**16**, and *rac-***17** had CSC EC_50_ values in the range of ∼1–10 μM (Figure 2). As the alkynylated derivative (−)-**21** was also able to retain high potency (16 nM) with selectivity against GBM CSCs (CSC maximum observed cell death, 92%; non-CSC maximum observed cell death, 27%), this compound may serve as a “clickable” probe for future mechanistic investigations. As expected, for the “unnatural” enantiomers (+)-**10**, (+)-**15**, and (+)-**16**, we observed limited activity against both CSCs and non-CSCs (Figure S1).

**Table 2:**
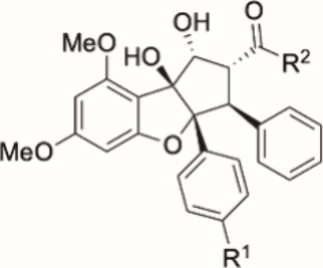
Preliminary SAR of Rocaglaic Acid (**10**) and its N-Acylated Derivatives against GBM CSCs and Non-CSCs

			CSCs		non-CSCs	
compound	R^1^	R^2^	EC_50_ (μM)^a^	max. observed efficacy (% dead cells)	EC_50_ (μM)^a^	max. observed efficacy (% dead cells)
(−)-**10**	OMe	OH	3.642	68	nd	27
(−)-**11**	Br	OH	0.121	92	nd	25
*rac*-**14**	OMe	NHCN	nd	44	nd	36
(−)-**15**	OMe	NHSO_2_Me	0.980	85	nd	19
(−)-**16**	OMe	NHSO_2_N(Me)_2_	1.728	76	nd	23
*rac*-**17**	OMe	NHSO_2_NHCH_2_CCH	8.083	54	nd	24
*rac*-**18**	OMe	NHSO_2_(CH_2_)_2_CCH	nd	40	nd	29
(−)-**19**	Br	NHSO_2_Me	0.095	92	nd	20
(−)-**20**	Br	NHSO_2_N(Me)_2_	0.045	93	nd	26
(−)-**21**	Br	NHSO_2_NHCH_2_CCH	0.016	92	nd	27
(+)-**10**	OMe	OH	7.085	58	nd	30
(+)-**15**	OMe	NHSO_2_Me	nd	34	nd	6
(+)-**16**	OMe	NHSO_2_N(Me)_2_	nd	49	nd	36

a For each cell type, EC_50_ values are provided for compounds causing at least 50% cell death. Values shown are relative EC_50_ values obtained from a variable-slope, four-parameter nonlinear regression constrained to bottom = 0% and top <100% cell death.

To further validate the observed CSC selectivity, compound (−)-**20**, one of the most potent compounds identified against GBM0308 CSCs, was also evaluated against additional GBM stemlike cell lines, including BT112 and BT145, both of which also showed selective susceptibility of CSCs versus non-CSCs (Figure S3), with CSC EC_50_ values of 34 and 93 nM determined against the two cell lines, respectively.

### Derivatization-Free Target Identification Using a Modified Proteome Integral Solubility Alteration Assay

We next pursued proteome-wide target identification to better understand the target profiles of CSC-selective rocaglates. To enable cross-compound comparisons of target engagement, we opted to use the proteome integral solubility alteration (PISA) assay,^29^ a derivative of the mass spectrometry-based cellular thermal shift assay (MS-CETSA). In MS-CETSA,^30,31^ target engagement is inferred through proteome-wide analysis of compound-induced changes to protein thermal stability; proteins with a shift in their half-maximal thermal denaturation temperature upon compound treatment are flagged as potential targets. The PISA assay obviates the need for MS-CETSA-derived thermal denaturation curves through curve integration by pooling of individual temperature points into a single “integral” sample for each treatment condition, thereby increasing throughput (Figure S4). In contrast to MS-CETSA (which relies on curve comparisons), PISA determinations are performed by simply comparing soluble protein abundance levels between compound- and DMSO-treated samples. Importantly, protein fold-change measurements in PISA experiments generally scale with the thermal shift derived from CETSA experiments, and the magnitude of a thermal shift can inform on relative affinity between compounds and individual targets.^30,32−35^ Maximal stability changes are protein-specific,^36^ however, precluding estimations of preference for one target over another. Thus, PISA allows for simultaneous target profiling and comparisons of the binding affinity between different compounds within a given target.

**Figure 3:**
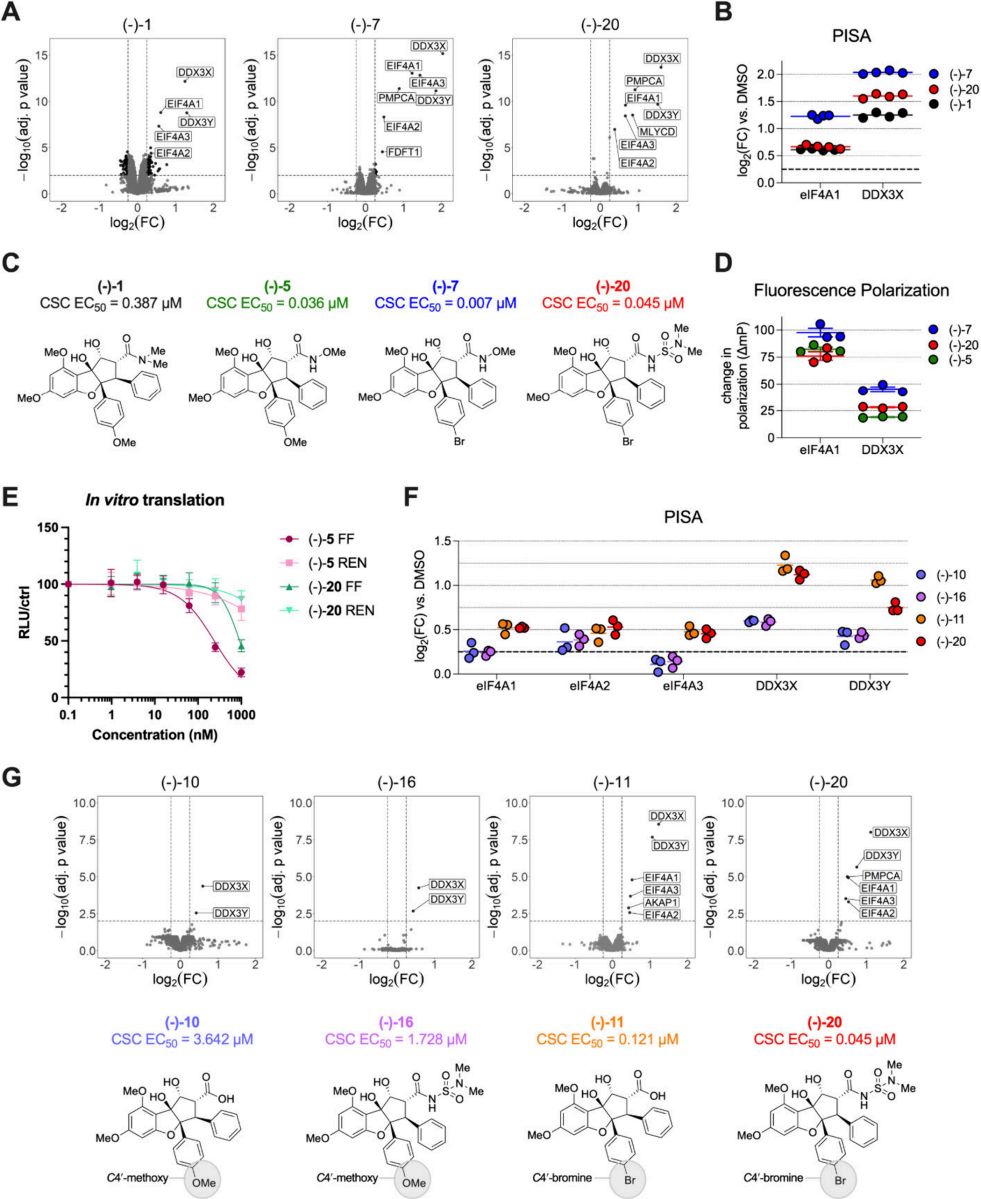
(A) Comparative PISA profiling. Volcano plots depicting differentially stabilized GBM0308 lysate proteins in the presence of the natural rocaglate (−)-**1** (left), derivative (−)-**7** (middle), and derivative (−)-**20** (right). Compounds were tested at a concentration of 10 μM. Significance thresholds were set to FDR < 0.01 with |log_2_(FC)| > 0.25. (B) Extracted individual log_2_(FC) (*N* = 4) from PISA assays for DDX3X and eIF4A1 stabilization in the presence of compounds (−)-**1**, (−)-**7**, and (−)-**20**. (C) Structures of compounds (−)-**1**, (−)-**5**, (−)-**7**, and (−)-**20** and their corresponding EC_50_ values against GBM CSCs. (D) Change in polarization (ΔmP) obtained with DDX3X:FAM-labeled r(AG)_8_ and eIF4A1:FAM-labeled r(AG)_8_ by FP assay in the presence of (−)-**5**, (−)-**7**, and (−)-**20** (10 μM). Background-subtracted values were generated using a DMSO control (*N* = 3; lines indicate mean ± SEM). (E) *In vitro* translation experiments testing the bicistronic mRNA reporter construct FF–HCV-Ren. The translation of the FF cistron is cap-dependent, while translation of the HCV-Ren cistron is driven off an HCV-IRES, rendering translation of this cistron cap-independent. (F) Extracted individual log_2_(FC) (*N* = 3) for DDX3 and eIF4A paralog stabilization in the presence of compounds (−)-**10**, (−)-**11**, (−)-**16**, and (−)-**20**. Horizontal bars indicate mean values. (G) Comparative PISA profiling of compounds (−)-**10**, (−)-**11**, (−)-**16**, and (−)-**20** in GBM0308 cell lysate, with volcano plots depicting differentially stabilized lysate proteins in the presence of *C4′*-methoxy ((−)-**10**, (−)-**16**) (left) and *C4′*-brominated ((−)-**11**, (−)-**20**) (right) compounds tested at a concentration of 10 μM. Significance thresholds were set to FDR < 0.01 with |log_2_(FC)| > 0.25.

We initially attempted to benchmark PISA for rocaglate target identification using **CR-1-31b** ((−)-**5**) (Figure 1A), a well-characterized polypurine RNA-dependent inhibitor of eukaryotic translation initiation factor 4A1 (eIF4A1).^37^ Using this compound, we found that a standard lysate-based PISA assay was not suitable for the detection of the rocaglate:protein:RNA complex. Following a detailed investigation,^38^ we found that the use of two key lysate additives, a polypurine (AG)_8_ RNA probe and the nonhydrolyzable ATP analog AMP-PNP satisfied the requirements for rocaglate:eIF4A1 target engagement, enabling detection of compound (−)-**5**-induced eIF4A1 stabilization. We then performed PISA in GBM0308 (CSC) lysates with compounds (−)-**1**, (−)-**7**, and (−)-**20** to assess the possibility of differential targeting driven by substitutions at the rocaglate R^1^ and R^2^ positions as suggested by our SAR studies (Tables 1 and 2). Consistent with previous reports,^17,18,39^ we detected thermal stabilization of eIF4A paralogs and DDX3X by (−)-**1** and confirmed that these proteins are also stabilized by (−)-**7** and (−)-**20** (Figure 3A). Of note, we also detected stabilization of DDX3Y, a paralog which shares >90% sequence similarity with DDX3X. DDX3Y had been speculated, but not yet shown, to be an additional target of (−)-**1** in male-derived cells.^18^ We then extracted the individual fold changes for eIF4A1 and DDX3X (Figure 3B) and noted that, interestingly, the relative stabilization of DDX3X among the three compounds ((−)-**7** ≫ (−)-**20** > (−)-**1**) more closely reflected the observed SAR differences in anti-CSC potency than did the compounds’ relative stabilization of eIF4A1 ((−)-**7** ≫ (−)-**20** ≈ (−)-**1**).

Given these observations, we next sought to interrogate the effects of compound (−)-**20** in secondary assays for both eIF4A and DDX3 engagement. To directly validate our PISA findings showing stabilization of both eIF4A1 and DDX3X, we conducted fluorescence polarization (FP) assays using a fluorescein amidite (FAM)-labeled r(AG)_8_ RNA probe developed previously.^17,40^ Under these conditions, we found that ATP was a necessary additive to enhance the degree of anisotropy change for both proteins compared with ADP + P_i_ (Figure S5). As shown in Figure 3D, compounds (−)-**5**, (−)-**7**, and (−)-**20** (10 μM concentration) all strongly stimulated the binding activity of eIF4A1 to RNA under these conditions, with the strongest clamping observed for (−)-**7**. For DDX3X, the *C4′*-brominated rocaglates (−)-**7** and (−)-**20** stimulated RNA binding to a greater extent than did *C4′*-methoxy rocaglate (−)-**5**. This trend held in FP-based binding affinity experiments wherein each of the helicases was titrated against the FAM-labeled (AG)_8_ RNA probe in the presence of 50 μM rocaglate and 1 mM ATP.^18,41^ In these experiments, compounds (−)-**5**, (−)-**7** and (−)-**20** showed similar potency and efficacy toward stimulation of eIF4A1:(AG)_8_ complex formation (Figure S6, top), whereas DDX3X:(AG)_8_ complex formation was clearly favored in the presence of (−)-**7**, followed by (−)-**20** and (−)-**5**, the latter of which was significantly less efficacious at stimulating binding at the tested concentrations (Figure S6, bottom). While inherent differences between the FP assay conditions (*e.g.*, probe affinity, protein mobility in FP buffer) preclude direct comparison of absolute ΔmP values across the two different proteins, we noted that relative anti-CSC potencies for these compounds ((−)-**7** > (−)-**5** ≈ (−)-**20**) were similar to their relative degrees of clamping for DDX3X and eIF4A1. To follow up on these findings, we performed *in vitro* translation experiments using a bicistronic mRNA reporter construct FF–HCV-Ren, where translation of the firefly luciferase (FF) cistron is cap-dependent while translation of the Renilla luciferase (HCV-Ren) cistron is driven off an HCV-IRES, rendering translation of this cistron cap-independent. Interestingly, we found that despite their near-equivalent CSC EC_50_ values, Roc ASF derivative (−)-**20** was found to be a significantly less potent cap-dependent translation inhibitor in this assay than (−)-**5** (EC_50_ = 937 nM vs 215 nM, respectively; Figure 3E).^42^

Based on our PISA and FP results confirming eIF4A and DDX3X stabilization for the four tested CSC-selective congeners, we next sought to directly interrogate the impact of *C4′-*bromine versus *C4′*-methoxy substitutions on the relative stabilization of these helicase targets. Accordingly, we performed comparative PISA profiling of two *C4′*-methoxy-substituted compounds ((−)-**10**, (−)-**16**) against two *C4′*-bromine-substituted congeners ((−)-**11**, (−)-**20**) in GBM0308 lysates. We again extracted individual fold changes for each compound and protein of interest and found that the *C4′*-brominated compounds exhibited greater effect sizes and statistical significance than their *C4′*-methoxy congeners (Figures 3F,G) across most targets. While all compounds showed some stabilization of eIF4A, only the *C4′-*brominated compounds stabilized all eIF4A paralogues, especially eIF4A3, beyond significance thresholds. A previous systematic study of hundreds of synthetic rocaglates from our laboratories showed that rocaglates are generally able to induce RNA clamping of eIF4A3 to an extent that is well-correlated with their degree of eIF4A1 clamping.^39^ However, these studies did not show significant differences in eIF4A1 or eIF4A3 RNA clamping between *C4′*-bromo/methoxy-substituted matched pairs (*e.g.* (−)-**5** and (−)-**7**) by FP, despite (−)-**7** frequently outperforming (−)-**5** in other assays such as FP clamping and translation inhibition.^43−45^ Nonetheless, eIF4A3 has been reported to play a role in ribosome biogenesis and may be an emerging target for cancer cells showing elevated rates of ribosome production.^46^ Taken together, our results suggest the importance of the *C4′-*bromine substitution in both improving potency against GBM CSCs and enhancing stabilization of all DEAD-box helicases, with striking effects on the stabilization of DDX3.

### Modeling DEAD-Box Helicase Engagement of the Roc ASF Derivative (−)-**20**

**Figure 4:**
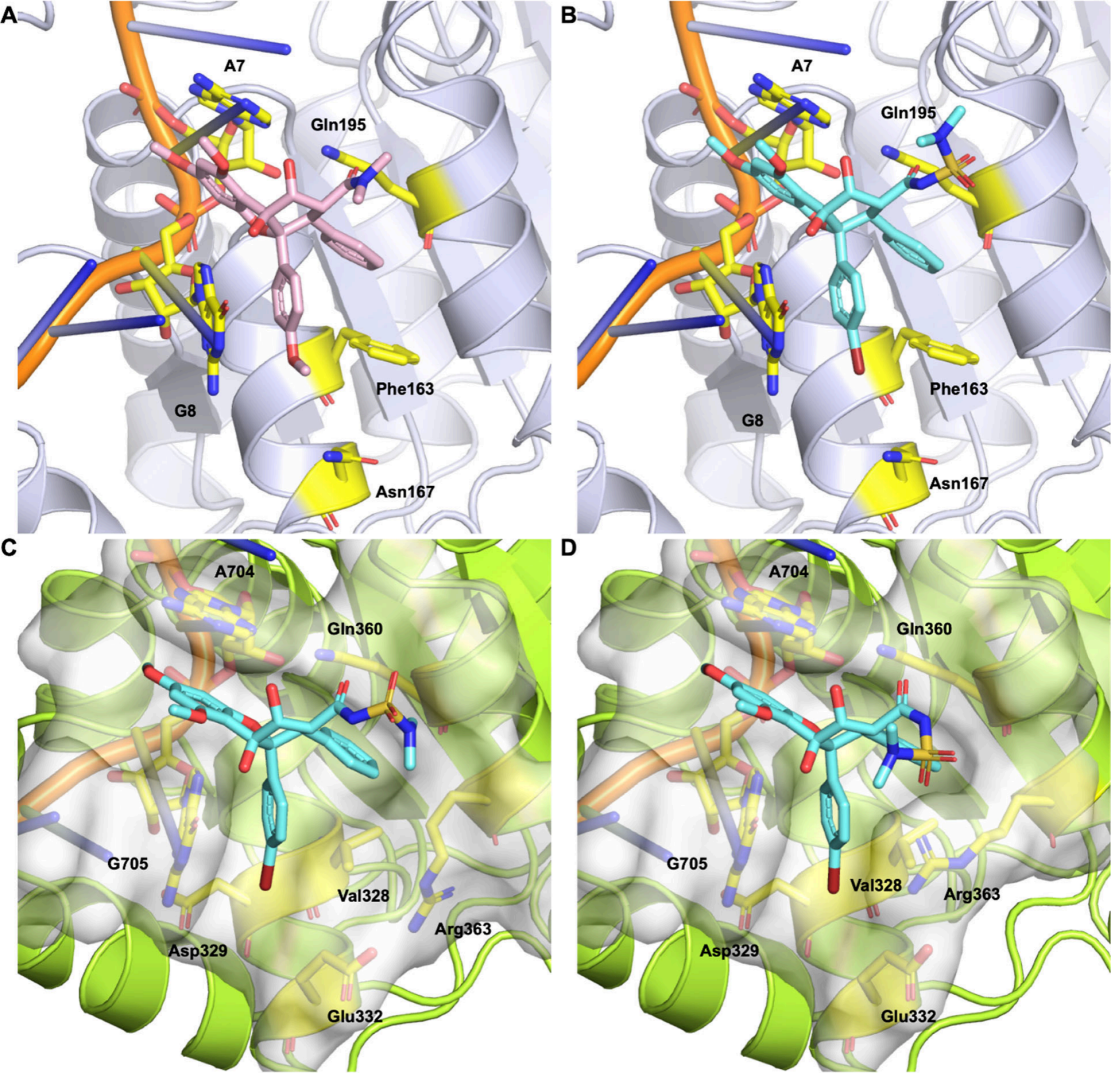
(A) X-ray cocrystal structure of RocA(**1**):eIF4A1:poly(AG) complex (PDB entry 5ZC9). (B) Glide docking pose for (−)-**20** into the 5ZC9 eIF4A1:poly(AG) structure (Glide G_score_ = −11.7 kcal/mol), showing a “canonical” rocaglate binding pose. (C) Top-ranked induced-fit docking pose for (−)-**20** (G_score_ = −7.0 kcal/mol), modeled from an X-ray crystal structure of DDX3X bound to a RNA:DNA hybrid (PDB entry 7LIU), modified with a single-residue mutation (C704A). (D) IFD pose (G_score_ = −6.1 kcal/mol) for (−)-**20** and the 7LIU-C704A receptor showing interaction between the ionized rocaglate acyl sulfamide and the cationic DDX3X residue Arg363. Movement of the Arg363 side chain creates a cleft into which the *C4′-*brominated B ring inserts.

We next sought to use computational modeling to predict and compare how the structural features present in CSC-selective rocaglates, namely, the *C4′-*bromo substituent and ionizable C2 substituent, may impact target engagement of eIF4A and DDX3 paralogs. An X-ray cocrystal structure of a RocA:eIF4A1:poly(AG) complex from the RIKEN group (PDB entry 5ZC9)^17^ shows that RocA acts as a bimodular inhibitor between eIF4A1 and RNA (Figure 4A). Specifically, the RocA C2 carbonyl is hydrogen-bonded to eIF4A Gln195, while the A and B rings engage in π–π stacking interactions with A7 and G8 of RNA, respectively. Lastly, the C ring engages in a parallel-displaced π-stacking interaction with eIF4A Phe163. Close inspection of this binding mode reveals that the C4′ substituent, a methoxy group in the liganded RocA, is largely solvent-exposed, projecting toward eIF4A1 residue Asn167. We envision that a bromine substituent could be slightly preferred to methoxy at this site based on both steric and electrostatic considerations and, depending on trajectory, may allow for halogen bonding of the Asn167 carbonyl. Interestingly, sequence alignments (Figure S7) show divergence at this residue among the PISA-identified helicase targets. While this residue is conserved as Asn in eIF4A2, it is substituted in eIF4A3 as Arg172, which may also discriminate between *C4′-*Br and *C4′-*OMe based on hydrogen bonding and steric considerations. To directly probe the ability of (−)-**20** to bind eIF4A1/RNA, we used Glide docking (Schrödinger, LLC) into the rocaglate binding site of the 5ZC9 X-ray structure. The top-scored pose (Glide G_score_ = −11.725 kcal/mol) was comparable to that observed in the experimentally determined RocA complex (Figure 4B), including all expected π-stacking interactions with the A, B, and C rings and a hydrogen bond between eIF4A1 residue Gln195 and the acyl sulfamide carbonyl, thus supporting the overall compatibility of the Roc ASF chemotype with eIF4A1.

We next sought to evaluate docking of (−)-**20** into DDX3X. Based on sequence and structural alignment between DDX3X and eIF4A1 (Figure S7), it is postulated that rocaglates may target a similar binding pocket near DDX3X residues Val328 (corresponding to eIF4A1 Phe163), Glu332, Gln360 (corresponding to eIF4A1 Gln195), and Arg363, of which Gln360 was previously shown to play a key role in binding RocA.^18^ In the same study, it was also shown through computational overlays to a DDX3X structure lacking a bound oligonucleotide (PDB entry 5E7M) that the phenyl C ring is likely sterically incompatible with DDX3X, requiring an alternate binding mode.^18^ We posited that sequence divergences between eIF4A1 and DDX3X at the rocaglate binding site may impact Roc ASF binding and sought to further probe this hypothesis through modeling.

For DDX3X modeling studies, we used the X-ray crystal structure of DDX3X bound to an ATP analogue and a remodeled RNA:DNA hybrid from Enemark and co-workers (PDB entry 7LIU). We selected this structure for modeling based on multiple factors. First, this structure was the only oligonucleotide-bound DDX3X structure available in a “post-unwound” conformation^47^ that appeared to be rocaglate-competent (backbone RMSD from 5ZC9 = 0.971 Å). In contrast, the only other oligonucleotide-bound DDX3X structure available depicts the protein in a “pre-unwound” state (PDB entry 6O5F, backbone RMSD from 5ZC9 = 21.917 Å).^47^ In addition, using transitive overlays of RocA from the eIF4A1 X-ray structure (5ZC9) (Figure S8A) to DDX3X structures 7LIU (Figure S8B) and 5E7M (Figure S8C), we observed that in the presence of the RNA:DNA hybrid, the binding site topology immediately adjacent to the RocA C ring offers a widened binding pocket (Figure S8B) that more closely resembles that observed for eIF4A1 (Figure S8A), thus mitigating the clear steric clashes observed in the non-oligo-bound 5E7M structure (Figure S8C) that were previously postulated to impact rocaglate binding.^18^

Proceeding with the 7LIU structure, we next used PyMOL (Schrödinger, LLC) to perform a single cytosine-to-adenine point mutation on the RNA:DNA hybrid (7LIU-C704A) based on the known preference of rocaglates to bind polypurine RNA. Accordingly, all residues within the docking grid used to define the ligand binding site were purine ribonucleotides (GGGAGGG), with all deoxyribonucleotides outside of the grid (Figure S9A). This polypurine sequence is consistent with tetramer motifs identified by a previously reported Bind-n-Seq experiment with DDX3X and RocA.^18^ Further, the mutated GAGG RNA tetramer immediately flanking the rocaglate binding site showed excellent conformational overlay to the analogous GAGA tetramer in the 5ZC9 structure (Figure S9B). Unfortunately, our efforts to model compound (−)-**20** at the predicted rocaglate binding site of both the 7LIU and 7LIU-C704A structures using conventional Glide docking failed to produce viable poses. Returning to our comparative overlays, we noted that the 7LIU and 5E7M DDX3X structures both show a variably positioned salt bridge between key binding site residues Glu332 and Arg363 (Figure S8B,C); we posited that despite a more accommodating C-ring binding pocket, clashes between Glu332 and the rocaglate B ring may have sterically impeded our attempt at “rigid-receptor” docking into this structure (Figure S8B). Accordingly, we examined Schrödinger’s induced fit docking (“IFD”), an alternative Glide docking workflow that accounts for the inherent propensity of proteins to undergo side-chain conformational changes in response to ligand binding. Prior studies have established that ∼90% of the rotatable side chains on receptor amino acids undergo subtle conformational changes in response to, and to accommodate, ligand binding.^48^ Thus, in IFD the receptor is treated as partially flexible; the protein backbone atoms (and in this experiment, all oligonucleotide atoms) are held rigid, while side-chain atoms are allowed to move to accommodate ligand binding. Fortunately, these IFD experiments produced multiple induced-fit binding poses with compound (−)-**20** positioned in the “canonical” rocaglate binding mode, albeit with significantly worse docking scores compared to those of our rigid-receptor docking into eIF4A1. In the top-scored IFD pose (G_score_ = −6.986 kcal/mol; Figure 4C), in addition to several subtle adjustments in the positioning of key binding pocket side chains (Figure S10A), we observed an ∼64° rotation of the Glu332 side-chain terminus, presumably to better accommodate the *C4′-*bromo substituent while retaining a salt bridge to Arg363 (Figure S10B). Interestingly, while the second highest IFD pose (G_score_ = −6.131 kcal/mol; Figures 4D and S10C) showed a nearly identical rotation of the Glu332 side chain, this pose also showed movement of the Arg363 side chain to engage in a hydrogen-bonding interaction with the sulfonyl from the ionized acyl sulfamide of (−)-**20** while still maintaining its salt bridge interaction with rotated Glu332 (Figure S10D). Furthermore, we observed that the movement of Arg363 allowed the *C4′-*bromine substituent to nestle in a shallow cleft lined by Arg363, Asp329, Glu332, and Val328 (Figure 4D). Beyond these key differences, both IFD poses show the expected “canonical” rocaglate–helicase interactions, with the phenyl A and B rings π–π-stacked to A704 and G705 of the oligonucleotide, respectively, and the expected hydrogen bonding between the acyl sulfamide carbonyl oxygen and the side chain of Gln360. The consistent ligand-induced rotation of the Glu332 side chain in both structures is notable given the previously noted steric clash of this residue with the B ring predicted by overlay and the clear preference for B-ring *C4′-*bromo (over *C4′-*methoxy) substitution that was illuminated in our SAR studies. Beyond the obvious steric implications of the slightly smaller bromine substituent, bromine was also found to exhibit a high propensity for interaction with arginine in a systematic study of halogenated ligands in the PDB (*cf.*Figure 4D).^49^

Taken together, our modeling studies reveal putative binding modes for compound (−)-**20** with both eIF4A1 and DDX3X, all of which must be validated experimentally. Indeed, given their comparatively low docking scores, our IFD-predicted DDX3X structures likely require substantial further refinement, ideally *via* structural biology of rocaglate:DDX3X:oligonucleotide cocomplexes. Nonetheless, the unique ligand-induced structural adjustments predicted by IFD overcome prior hurdles encountered in modeling rocaglate–DDX3 interactions and suggest a possible rationale for our experimental data clearly showing both improved CSC selectivity and improved DDX3 target engagement for *C4′-*brominated rocaglates over their *C4′-*methoxy-substituted counterparts. In addition, our IFD structure showing interaction between the acyl sulfamide sulfonyl and cationic Arg363 raises the provocative question of whether the observed preference for ionizable C2 substituents may be reflective of ionic engagement of Arg363.

### Roc ASF (−)-**20** Inhibits Pathways and Genes Required for GBM Stem Cell Survival

Rocaglates, including (−)-**1** and (−)-**5**, have been extensively characterized by our group and others as potent translation inhibitors by way of stimulating the binding of DEAD-box helicases eIF4A1/2 and, in the case of (−)-**1**, DDX3X to polypurine mRNAs.^18^ When considering the potential cytotoxic impacts of helicase clamping on GBM CSCs, it is important to note that despite rocaglates’ strong inhibition of protein synthesis *via* induced clamping of these targets, the phenotypic impacts of rocaglate treatment are distinct from those arising from the loss of these helicases or the direct inhibition of their helicase function. In fact, eIF4A-mediated RNA helicase unwinding activity is stimulated by rocaglate translation inhibitors, which show a dominant-repressive inhibitory effect on translation that is further enhanced in the presence of additional helicase target.^41^ Rocaglates promote unscheduled clamping of eIF4A (and DDX3X) to purine-rich segments of mRNA, which in the case of eIF4A impedes recycling of the helicase through its parent eIF4F complex.^16^ The lack of turnover prevents eIF4A’s continued participation in the initiation of eukaryotic translation, leads to stalling of ribosomes as they clamp in an unscheduled manner along the 5′-UTRs of mRNAs, and induces downstream translation repression of non-purine-rich mRNAs due to *trans*-inhibitory effects stemming from eIF4F depletion.^16^ Similarly, DDX3X has been shown to exhibit “dominant-negative” sensitivity to (−)-**1**,^18^ and it is also known to regulate RNA processing in a nonprocessive manner.^47^ It is reasonable to assume that unscheduled clamping of DDX3X to mRNAs would similarly impede catalytic turnover with potential downstream impacts on multiple DDX3X-mediated processes. While the diverse functions of DDX3X include participation in both cap-dependent and cap-independent translation initiation, DDX3X has been shown to exert both stimulatory and suppressive effects on translation, depending on circumstances.^50,51^ Here we observed a paradoxical reduction in translation inhibition potency for (−)-**20** (EC_50_ ≈ 1 μM) compared to its anti-CSC potency (EC_50_ = 45 nM) that starkly contrasts to (−)-**5** (translation EC_50_ ≈ 200 nM, CSC EC_50_ = 36 nM). These results suggest that other DDX3X-driven pathways unique to GBM CSCs may also be affected. There are also limitations to our current ability to characterize the full breadth of helicase targeting for (−)-**20** and other rocaglates. Given the broad and consistent stabilization of multiple helicases observed in PISA, it is possible that the clamping potentiation of (−)-**20** may extend to additional helicase targets that were not detected in PISA due to confounding factors such as inadequate protein coverage or incompatibility of the additive mRNA substrate (*e.g.*, lack of necessary secondary structural features for helicase recognition). We note that our recently reported PISA studies on (−)-**5** in A549 cells detected stabilization of additional DEAD-box helicases (*e.g.*, DDX21) that were not found to be stabilized in our PISA experiments in GBM CSCs by any rocaglates tested.^38^ Nonetheless, our results here suggest that the potency of Roc ASF-mediated CSC death, which in the case of (−)-**20** is ∼20-fold higher than its effects on cap-dependent translation, may arise from either additive or synergistic convergence of multiple gain-of-function processes that extend beyond the inhibition of protein synthesis. Further, these results underscore the specific utility of Roc ASF probes versus other CSC-selective rocaglates such as (−)-**7** (which in contrast to (−)-**20** is an exceptionally potent eIF4A clamper and cap-dependent translation inhibitor) in teasing out subtle mechanistic differences arising from the interplay of differential RNA helicase targeting.^16,39,43^

**Figure 5:**
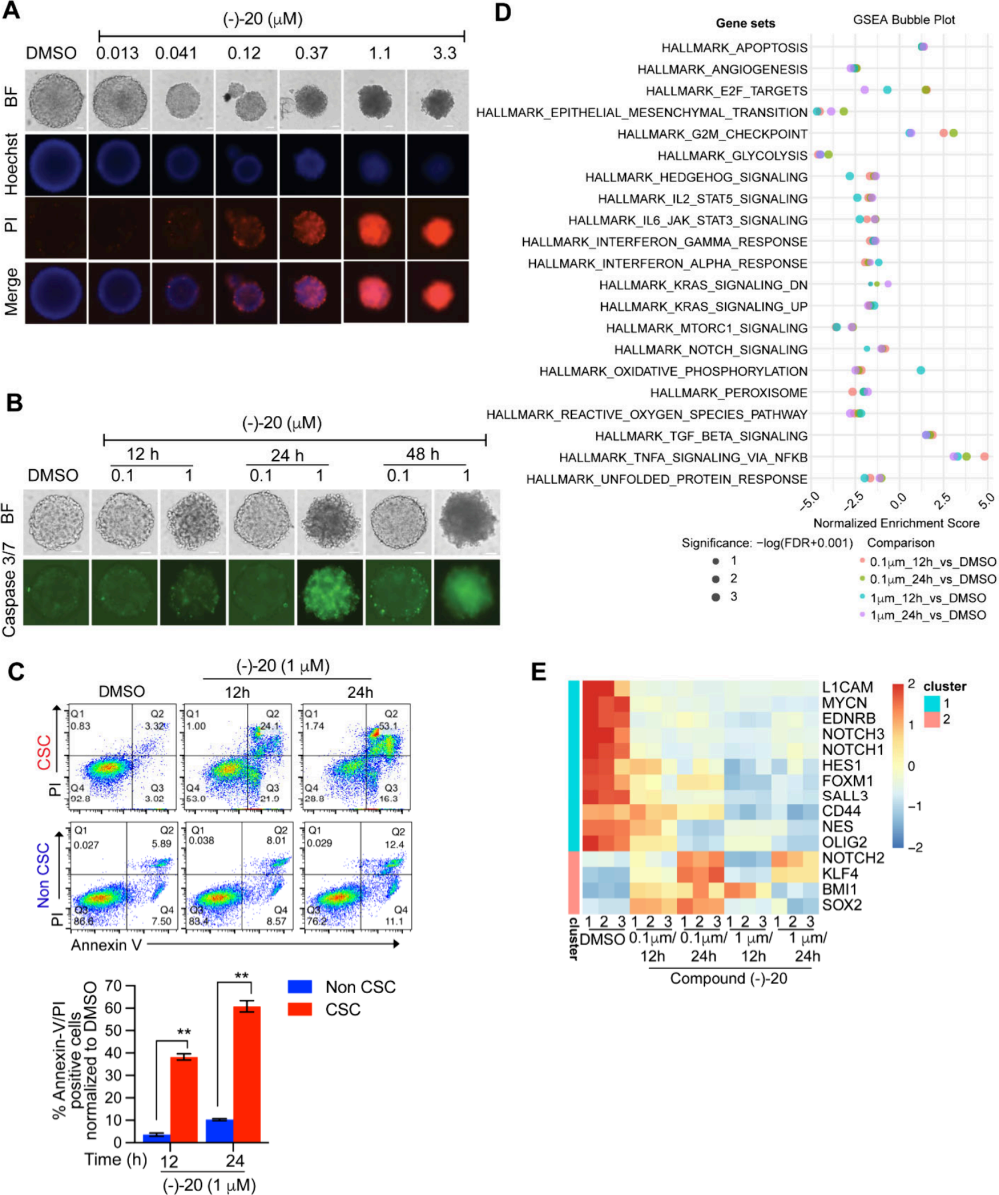
(A) Representative images showing propidium iodide (PI) and Hoechst staining in GBM0308 neurospheres treated with either DMSO or varying doses of compound (−)-**20** for 72 h, acquired using the Celigo image cytometer. Merge images (PI + Hoechst) are also shown. BF, bright-field. (B) Representative images showing caspase 3/7 staining in GBM0308 neurospheres treated with DMSO, 0.1 μM (−)-**20**, or 1 μM (−)-**20** acquired at the indicated times using the Celigo image cytometer. (C) Representative flow cytometry dot plots showing annexin-V/PI staining following treatment with either DMSO or (−)-**20** (1 mM) for 12 and 24 h, respectively (top), and quantification of percent annexin-V- and PI-positive cells normalized to respective DMSO controls (bottom). Data are presented as mean ± SD from two independent experiments, and *p* values were calculated using a two-tailed unpaired *t* test (**, *p* < 0.01). (D) GSEA multibubble plot. The color of the bubble represents the comparison, the size of the bubble represents the significance, and the *x* axis represents the normalized enrichment score (NES). All gene sets with FDR < 0.05 and |LFC| > 0.585 in any of the five comparisons are included here. (E) Z-score heat map visualizing differentially expressed genes (DEGs) involved in CSC stemness. DEG signatures in GBM0308 stem cells treated with either DMSO or compound (−)-**20** for the indicated times and concentrations are shown. Only significant genes (FDR < 0.05 and |LFC| > 0.585) are presented here. Hierarchical clustering was performed using the complete linkage method and 1-Pearson as the distance. The genes were then classified into two clusters based on the dendrogram.

To gain additional mechanistic insights into GBM cell death induced by (−)-**20**, we conducted several functional assays. We first derived single neurospheres from GBM0308 cells cultured in stem cell conditions, which were treated with either DMSO or (−)-**20** at varying doses for 3 days followed by staining with PI and Hoechst dyes and subsequent imaging. The results presented in Figure 5A show that (−)-**20** exhibits notable cytotoxic effects on CSC neurospheres, as evidenced by PI staining starting from a concentration of 0.12 μM onward. Additionally, a significant dose-dependent reduction in the neurosphere size was observed. Notably, when the neurospheres were treated with RK-33, a commercially available small-molecule inhibitor of DDX3,^52^ cytotoxic effects were also observed, with >3 μM concentration required to observe a noticeable effect on neurosphere size (Figure S11). Overall, these results suggest that (−)-**20** is significantly more potent in eliminating GBM CSCs than RK-33. To capture the kinetics of apoptotic events, we next employed caspase 3/7 staining of GBM0308 neurospheres that were treated with either DMSO or (−)-**20** and subsequently evaluated at three time points over a 48 h period. Caspases are a family of enzymes involved in apoptosis, and specifically, caspase-3 and caspase-7 are key players in the execution phase.^53^ We observed positive staining of caspases 3/7 in cells treated with 1 μM (−)-**20** at 24 and 48 h time points, indicative of apoptosis (Figure 5B). We further performed flow-cytometry-based analysis of Annexin V/PI staining in GBM0308 cells at these same time points, confirming that 1 μM (−)-**20** treatment induced apoptosis in GBM CSCs (Figure 5C).

Next, to further determine the role of DDX3X in GBM CSCs, we performed a knockdown experiment in GBM0308 cells. A short-hairpin RNA (shRNA)-mediated knockdown of DDX3X reduced GBM0308 growth in the culture (Figure S12), suggesting that DDX3X genetic inhibition may indeed impact the proliferation of GBM CSCs.

To understand the transcriptomic changes underlying the effects of (−)-**20** on GBM CSCs, we performed RNA sequencing experiments in GBM0308-derived neurospheres treated with (−)-**20**. Gene set enrichment analysis (GSEA) across all hallmark gene sets in the Molecular Signature Database (MSigDB) showed that the upregulated hallmark gene sets in (−)-**20** treated condition were related to apoptosis, G2/M checkpoint, and TNFα and NF-κB signaling pathways (Figure 5D). Though NF-κB regulation is often associated with cell survival,^54^ increasing evidence suggests that TNF receptors mediate alternate cell death pathways.^55−58^ Interestingly, earlier studies underscored the interplay between RNA helicase DDX3 and the NF-κB subunit p65, shedding light on DDX3 involvement in regulating transcriptional activity within the NF-κB signaling pathway.^59^ It is possible that disruption of the TNF-α–NF-κB signaling cascade by compound (−)-**20** may contribute to the TNF-mediated induction of cell death pathways in GBM CSCs. Furthermore, upon compound treatment, we observed the downregulation of several hallmark gene sets associated with critical cellular processes, including glycolysis, MTORC1 signaling, NOTCH signaling, epithelial–mesenchymal transition (EMT), and angiogenesis (Figures 5D and S13A). These data suggest that (−)-**20** may inhibit multiple oncogenic pathways in GBM CSCs. This observation emphasizes the complexity of cell death mechanisms and the potential origins of the selectivity of (−)-**20** against CSCs. Further analysis also revealed that treatment with (−)-**20** downregulated the expression of genes previously shown to be involved in stem cell maintenance and survival, notably, NOTCH1, NOTCH2, NOTCH3, SALL3, and SOX2. In addition, the stem cell marker CD44 was also downregulated in (−)-**20**-treated CSCs (Figure 5E).

The expression level of DDX3X in GBM has been previously reported to be significantly higher than in normal brain tissue.^60^ Through an analysis of 31 patient-derived GBM samples, Sun and co-workers reported a significant correlation between high levels of DDX3 and Snail, a transcription factor known to drive EMT and cancer metastasis.^61^ Recently, Brai and co-workers identified BA103 as a micromolar CC_50_ anti-GBM agent blocking the helicase activity of DDX3X, further underscoring DDX3X-targeted small molecules as promising drug leads for GBM.^62^ Additionally, Kerr and co-workers reported that DDX3 was highly expressed in pluripotent stem cells, such as embryonic stem cells (ESCs) and embryonal carcinoma cells (ECCs), and that inhibition of DDX3 using the DDX3X inhibitor RK-33 decreased the proliferation of undifferentiated stem cells.^63^ In agreement with previous literature, we showed that RK-33 had effects on GBM CSC neurospheres, albeit at much higher drug concentrations than for Roc ASFs. In our transcriptomic profiling experiments in GBM CSCs, we also found that the expression of several genes downstream of DDX3X were affected after treatment with compound (−)-**20** (Figure S13B),^64^ including FOXM1, a transcription factor downregulated by inhibition of DDX3X. Shriwas and co-workers reported that inhibition of DDX3 reduced CSC populations in oral squamous cell carcinoma with suppressed expression of FOXM1.^65^ Notably, other genes that have roles in CSC maintenance and survival were also downregulated upon treatment with (−)-**20**, which likely contributes to its potent anti-CSC activity.

## Conclusion

Our study reports the first identification of rocaglate congeners, including novel rocaglate acyl sulfamide (Roc ASF) derivatives, as selective inhibitors of glioblastoma (GBM) cancer stem cells. To access Roc ASFs, we developed new synthetic methods employing rocaglate β-lactone ring opening with nitrogen nucleophiles as a key step. A systematic dose–response study of rocaglaic acid *N*-acylated derivatives also established clear structure–activity relationships (SARs) for potent GBM cancer stem cell (CSC) targeting. Notably, we determined that *C2*-acyl sulfamoylation and *C4′-*bromination of the rocaglate scaffold both play important roles in improving potency and selectivity of rocaglates against GBM CSCs, with compound (−)-**20** showing high potency against GBM CSCs (EC_50_ = 45 nM) with limited detectable effects on GBM non-CSCs up to 10 μM and significantly dampened cap-dependent translation inhibition. We also assessed compound (−)-**20** in different GBM stemlike cell lines, including BT112 and BT145, both of which showed similar selective killing toward CSCs versus non-CSCs. Our study utilized a novel adaptation of PISA involving a polypurine RNA probe and the ATP analogue AMP-PNP as additives to assay for rocaglate:DEAD-box helicase target engagement. Additional mechanistic experiments and computational modeling of Roc ASFs implicate DDX3X and eIF4A paralogues as relevant targets contributing to the observed cytotoxic effects against CSCs. Using both PISA- and FP-based comparisons, we found that SAR trends for CSC potency and selectivity tracked more consistently with the relative strength of DDX3X engagement than that of eIF4A1 engagement or cap-dependent translation inhibition.^18^

Overall, the targeted array of derivatives and technologies used in our study have expanded our understanding of DEAD-box helicase targets for rocaglates and support the potential of designed rocaglates as CSC agents. Further development of Roc ASFs, including the synthesis and optimization of targeted congeners, is currently in progress and will be reported in future publications. Future studies will also aim to define the set of mRNAs that are regulated by (−)-**20** and related compounds *via* DDX3 in GBM stem cells using ribosome profiling, RNA Bind-n-Seq,^41^ and RNA-seq,^66,67^ techniques which have been reported using rocaglates, and potentially PAR-CLIP, which has been used to identify binding between DDX3 and helix 16 on the human 40S ribosome^68^ and to isolate RNA transcripts that copurify with endogenous eIF4A1 in MYCN-amplified neuroblastoma cells in the presence of an amidino rocaglate (ADR) derivative.^69^

## Methods

### Cell Culture

Glioblastoma stem cell lines GBM0308, BT145, and BT112 were cultured in neurobasal medium (NBE) (ThermoFisher Scientific) containing N-2 and B-27 supplements (ThermoFisher Scientific), EGF (Stem Cell Technologies), bFGF (Stem Cell Technologies), l-glutamine (ThermoFisher Scientific), and penicillin–streptomycin (Sigma-Aldrich) as described previously.^19^ Adherent GBM cells were cultured in Dulbecco’s modified Eagle’s medium (DMEM) (HyClone) supplemented with 10% v/v fetal bovine serum (FBS) (Atlanta Biologicals). Cells were incubated at 37 °C and 5% CO_2_ to allow neurosphere formation. For seeding and subculturing, the neurospheres were gently trypsinized with 0.025% trypsin to form a single-cell suspension.

### Dose–Response Testing in GBM Cells

Briefly, on day 1, GBM0308 cells were seeded in triplicate. For CSCs, 1500 cells/well were plated in a low-attachment 96-well plate with NBE medium and were incubated for 3 days at 37 °C and 5% CO_2_ to allow the formation of neurospheres. For non-CSCs, 3000 cells/well were plated in a 96-well plate with DMEM supplemented with 10% FBS. Similarly, the assay plates were also incubated for the same duration. Compound treatments started on day 4. Each compound was resuspended in DMSO and then diluted to different concentrations in media to a final concentration of DMSO of less than 1%. The compounds were added at concentrations of 10, 3.3, 1.11, 0.37, 0.12, 0.041, 0.013, and 0.004 μM, respectively. DMSO-treated cells served as a control. After 72 h of incubation with the compounds, the fluorescent stains propidium iodide and Hoechst 33342 (Life Technologies, Carlsbad, CA) were used to stain the GBM cells to determine cell viability. A staining solution in 1× PBS was prepared by mixing PI and Hoechst 33342 to working concentrations of 2.5 and 20 μM, respectively. Then 20 μL of this staining solution was added per well, and plates were incubated at 37 °C and 5% CO_2_ for 60 min. After incubation, plates were read by a Celigo image cytometer (Nexcelom Bioscience) using an inbuilt application, Cell Viability (Dead + Total). For the viability measurement, the total numbers of neurospheres/cells and dead neurospheres/cells in each well were counted by the cytometer. The percentage of PI+ cells was normalized to DMSO and plotted against the drug concentration to calculate the EC_50_ with a four-parameter, variable-slope dose–response curve with an upper constraint of <100% dead cells and a lower constraint of 0% dead cells (GraphPad Prism v.10.0.0). For all dose–response experiments, the experimental maximal efficacy (maximum observed % cell death) is reported. Relative EC_50_ values are reported for all compounds killing >50% of cells.

### Neurosphere Formation Assays

Single neurospheres were generated by seeding GBM0308 cells (400 cells/well) in a 384-well ultralow attachment round-bottom microplate (Nexcelom Bioscience, ULA-384 U). The plates were then centrifuged at 300*g* for 10 min to cluster cells at the bottom of the wells, followed by incubation at 37 °C and 5% CO_2_ for 4 days to allow the formation of single neurospheres, and treated with either DMSO or drugs at concentrations of 3.3, 1.11, 0.37, 0.12, 0.041, 0.013, and 0.004 μM. On day 7, neurospheres were stained with propidium iodide and Hoechst as described above. The “neurosphere 1 + Mask” application was used to measure the fluorescence intensities of PI using the Celigo image cytometer as described previously.^70^

### Apoptosis Assays

For the caspase 3/7 assay, single neurospheres were generated as described above. On day 4, neurospheres were treated with compound (−)-**20**. Apoptosis in treated tumorspheres was assessed using Nexcelom’s ViaStainTM Live Caspase 3/7 Detection Kit (Nexcelcom, CS1-V0002-1). The kit consists of a nucleic acid-binding dye with a fluorescent probe attached to a four amino acid peptide sequence DEVD (Asp-Glu-Val-Asp), forming a cell-membrane-permeable DEVD:DNA complex. During apoptosis, caspase 3/7 proteins cleaved the DEVD:DNA dye complex and released the high-affinity DNA binding dye, producing a bright-green fluorescence signal. After treatment, wells were stained with caspase dye (2 μM) at multiple time points, and plates were incubated for 60 min at 37 °C. Plates were analyzed by the Celigo image cytometer using the “neurosphere 1 + Mask” application to measure fluorescence intensities of the caspase dye as described previously.^70^ Apoptosis was quantified using a FITC Annexin V apoptosis detection kit II (Invitrogen). Annexin V staining was done per the manufacturer instructions. GBM0308 neurospheres were treated with (−)-**20** at 1 μM concentration. At 12 and 24 h after treatment, neurospheres were gently dissociated using 0.025% trypsin, stained with PI and FITC, and analyzed by flow cytometery using a Bio-Rad ZE5 cell analyzer.

### Chemical Synthesis

Detailed procedures for the chemical synthesis and characterization of new compounds are provided in the Supporting Information.

### Other Methods

Detailed protocols for the modified PISA assay, fluorescence polarization (FP) and *in vitro* translation assays, computational methods, RNA sequencing and analysis, and DDX3X knockdown experiments employing GBM0308 cells can also be found in the Supporting Information.

## Data Availability

The crystal structure of (+)-rocaglaic acid (−)-quinine salt **24** has been deposited in the Cambridge Crystallographic Data Centre (CCDC 2305159). The RNASeq data have been deposited in the GEO repository under accession number GSE246936. The mass spectrometry proteomics data have been deposited to the ProteomeXchange Consortium *via* the PRIDE partner repository with the data set identifier PXD047006.

## Supporting Information Available

Crystallographic data for compound **24** (CIF)
Supplementary figures, detailed experimental procedures for chemical synthesis and characterization, experimental protocols for the modified PISA assay, FP assays, *in vitro* translation assays, computational methods, RNA sequencing and analysis, and DDX3X knockdown experiments in GBM0308 cells (PDF)

## Supplementary Material

oc4c01073_si_001.cif
