# Supplementary material for: Identification of Rocaglate Acyl Sulfamides as Selective Inhibitors of Glioblastoma Stem Cells
Source: ACS Cent Sci. 2024 Aug 8;10(8):1640–56. doi: 10.1021/acscentsci.4c01073 (PMC11363328; doi:10.1021/acscentsci.4c01073)
Supplement: Supplementary file 2 — oc4c01073_si_002.pdf [file oc4c01073_si_002.pdf]

Supporting Information

Identification of Rocaglate Acyl Sulfamides (Roc ASFs) as Selective Inhibitors of Glioblastoma Stem Cells

Zihao Wang[1,7], Ritesh P. Thakare[2,7], Shalaka Chitale[2], Alok K. Mishra[2], Stanley I. Goldstein[3,4], Alice C. Fan[1,3], Rui Li[2,5], Lihua Julie Zhu[2,5], Lauren E. Brown[1], Regina Cencic[6], Sidong Huang[6], Michael R. Green[2,8], Jerry Pelletier[6,9], Sunil K. Malonia[2,*] , and John A. Porco, Jr.[1,3,10,*]

AUTHOR ADDRESSES

[1]Department of Chemistry and Center for Molecular Discovery (BU-CMD), Boston University, 590 Commonwealth Avenue, Boston, MA 02215, United States

[2]Department of Molecular, Cell and Cancer Biology, University of Massachusetts Chan Medical School, Worcester, MA 01605, United States

[3]Boston University Target Discovery Laboratory (BU-TDL), Boston, MA 02215, United States

[4]Department of Pharmacology, Physiology, and Biophysics, Boston University, Boston, MA 02118, United States

[5]Department of Molecular Medicine, and Program in Bioinformatics and Integrative Biology, University of Massachusetts Chan Medical School, Worcester, MA 01605, United States

[6]Department of Biochemistry, McGill University, Montreal, QC H3G 1Y6, Canada

[7]These authors contributed equally

[8]Deceased February 10, 2023

[9]Deceased September 17, 2023

[10]Lead contact

*Corresponding authors: sunil.malonia@umassmed.edu (S.K.M.); porco@bu.edu (J.A.P., Jr.)

Table of Contents

I. Supplementary Figures S2

II. Supplemental Methods for Biology and Proteomics S10

III. Computational Methods S14

IV. General Methods for Chemical Synthesis S15

V. Chemical Synthesis Procedures and Compound Characterization S16

VI. Select NMR Spectra S22

VII. X-ray Crystallographic Data for Compound 24 .... S35

VIII. Supplementary References S36

I. Supplementary Figures

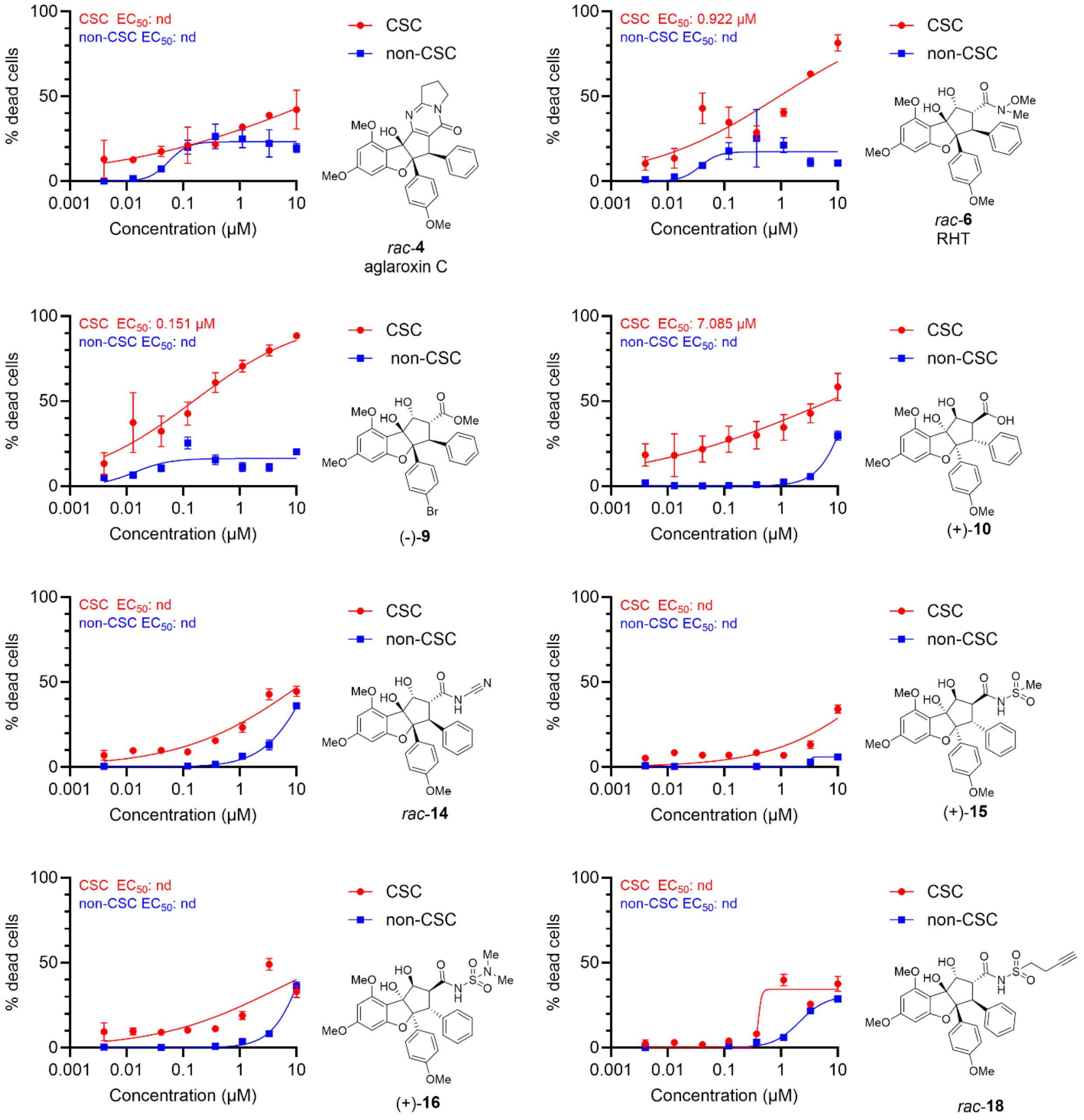

Figure S1. Dose-response curves and chemical structures for all tested compounds (**Table 1** and **Table 2**) not appearing in **Figures 1B** and **2**.

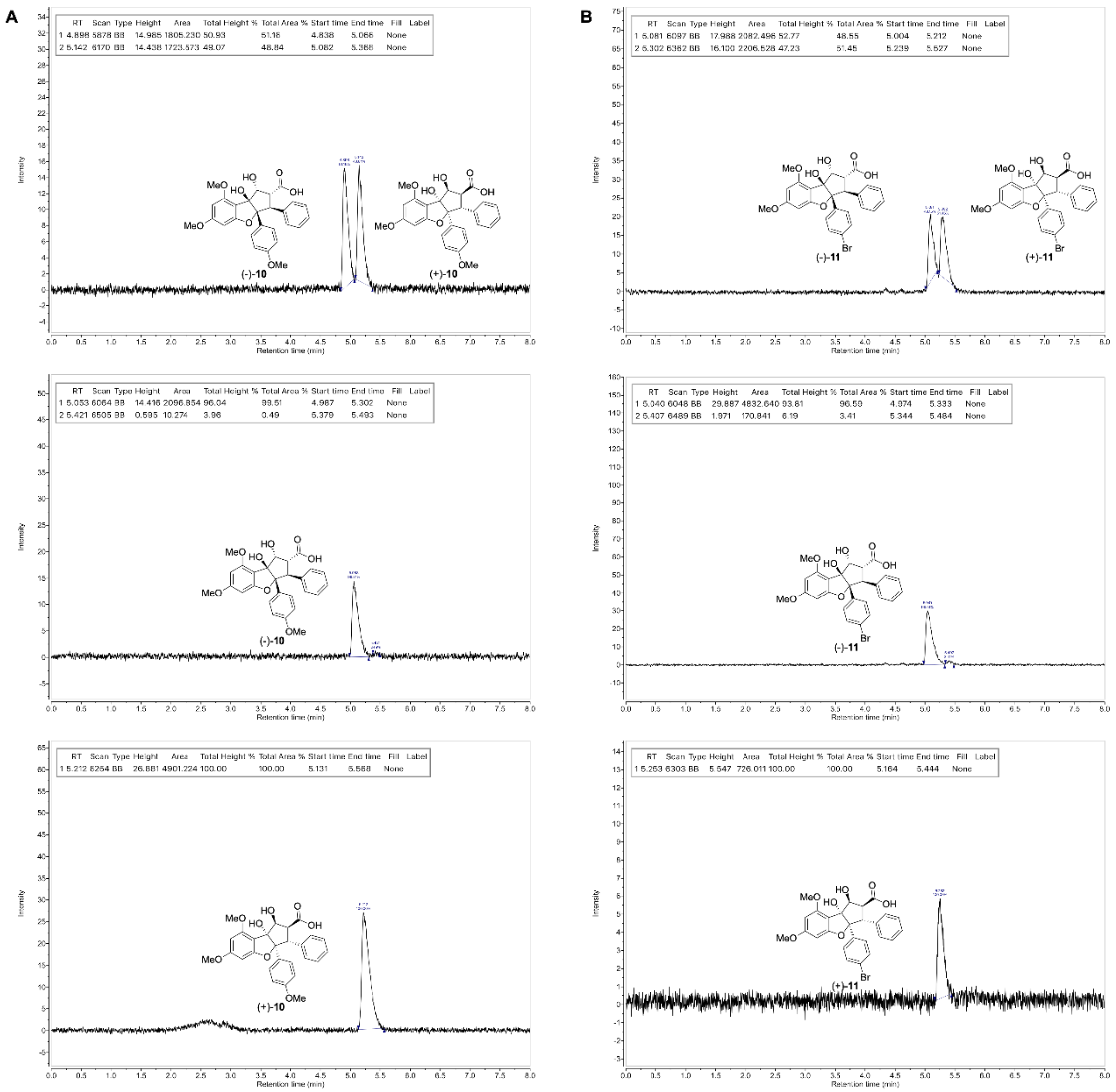

Figure S2. Chiral UPC[2] analysis for enantioenriched rocaglaic acid. Column: 2.5 µm Trefoil CEL2; Method: 5 % to 40 % MeOH in supercritical CO_2 over 4 minutes and 40 % MeOH in supercritical CO_2 for 2 minutes.

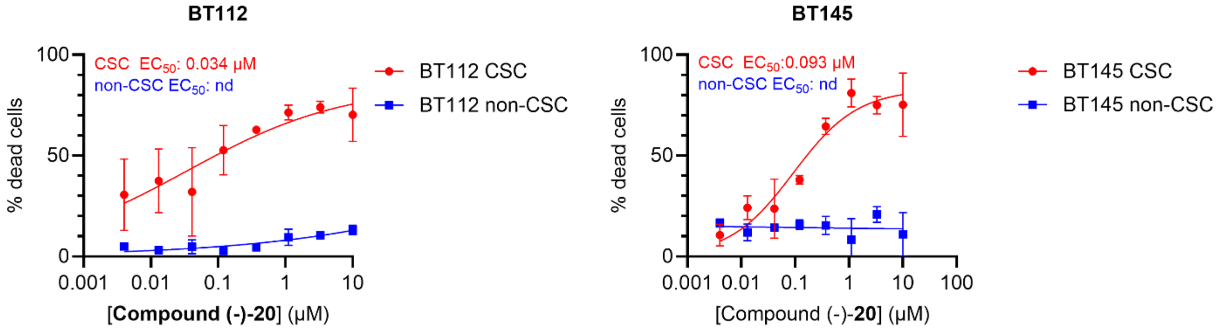

Figure S3. Dose-response curves comparing the killing of CSC (*red*) and non-CSC (*blue*) populations using compound (-)-**20** in BT112 (*left*) and BT145 (*right*) glioblastoma cell lines.

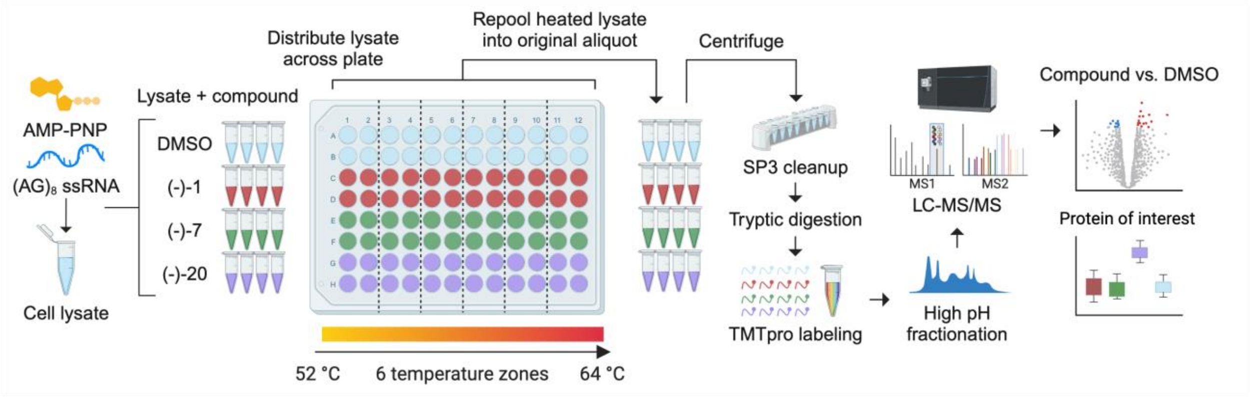

Figure S4. Schematic of the PISA workflow employed in this study. Cell lysates were pre-loaded with AMP-PNP and $(AG)_8$ RNA, treated with test compounds, then aliquoted across a PCR plate and heated to one of six different temperatures. For each sample corresponding to a single TMTpro plex, the six individually heated lysate aliquots were then repooled back into a single "integral" tube. The 16 repooled samples were centrifuged to remove aggregates, subjected to an SP3-based cleanup, digested with trypsin, then labeled with TMTpro 16plex reagents and combined. Peptides were fractionated and analyzed *via* LC-MS/MS. Protein abundances were compared between DMSO and compound treated samples using a moderated t-test with a Benjamini-Hochberg correction.

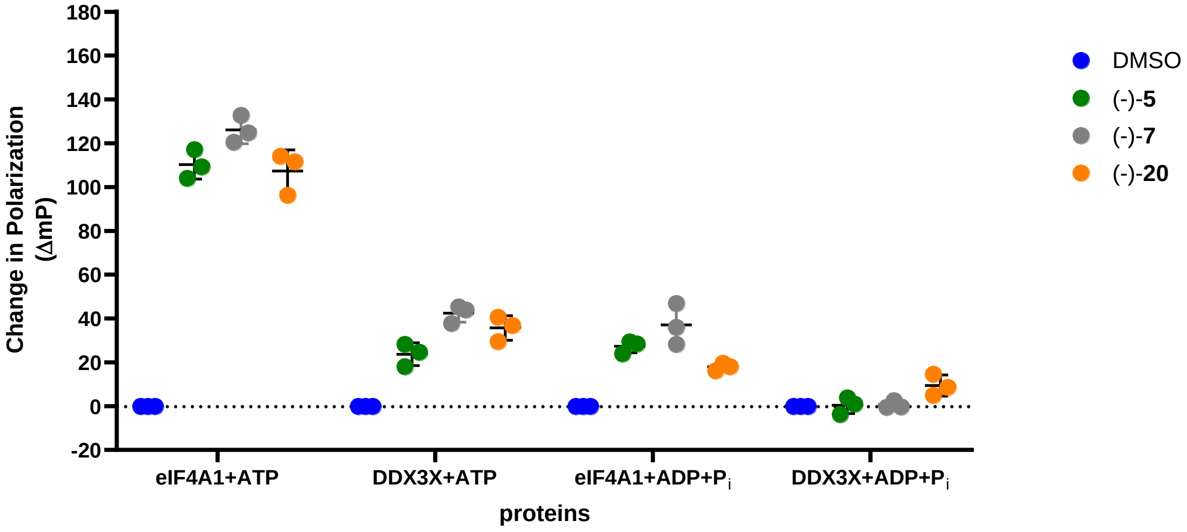

Figure S5. ATP independent clamping of eIF4A1 and DDX3X. Fluorescence polarization assay was performed to assess change in polarization (ΔmP) obtained with eIF4A1:FAM-labeled poly $r(AG)_8$ and DDX3X:FAM-labeled poly $r(AG)_8$ in the presence of (-)-**5**, (-)-**7**, or (-)-**20** (10 μM) and either 1 mM ATP or 1 mM ADP+P_i. N = 3 ± SEM.

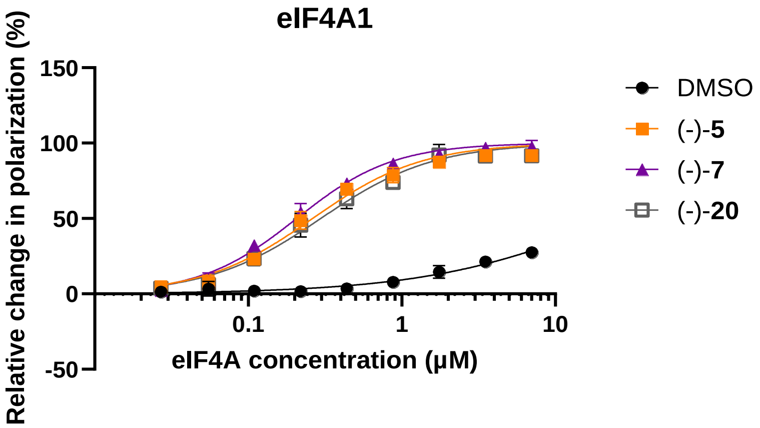

Condition	EC_{50}
DMSO	nd
(-)-**5** (50 μM)	263 nM
(-)-**7** (50 μM)	207 nM
(-)-**20** (50 μM)	296 nM

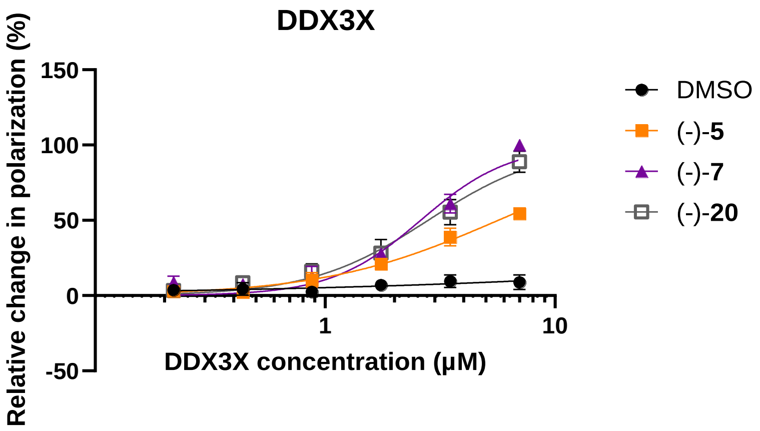

Condition	EC_{50}
DMSO	nd
(-)-**5** (50 μM)	5.7 μM
(-)-**7** (50 μM)	2.6 μM
(-)-**20** (50 μM)	2.8 μM

Figure S6. Protein titration experiments measuring rocaglate-induced stimulation of complex formation between DEAD-box proteins and a FAM-labeled $(AG)_8$ RNA probe. *Top*: Rocaglate (50 μM) stimulation of eIF4A1 binding to $(AG)_8$ RNA probe (10 nM) in the presence of ATP (1 mM) shows comparable stimulation by all three compounds relative to DMSO, with EC_{50}s for complex formation between 200-300 nM. *Bottom*: Rocaglate stimulation of DDX3X binding to $(AG)_8$ RNA probe in the presence of ATP shows significant enhancement of binding stimulation for *C*4′-brominated congeners (-)-**7** and (-)-**20** over *C*4′-methoxy substituted (-)-**5**. N = 3 ± SEM.

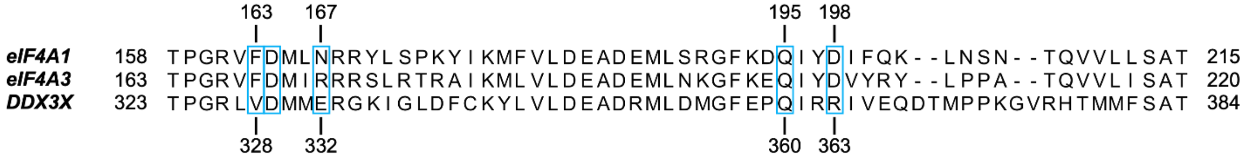

Figure S7. Sequence alignment for eIF4A1, eIF4A3, and DDX3X protein sequences at the rocaglate binding site. Key residues at the rocaglate binding site are boxed in light blue. Residue labels reflect eIF4A1 (*top*) and DDX3X (*bottom*) sequence numbering.

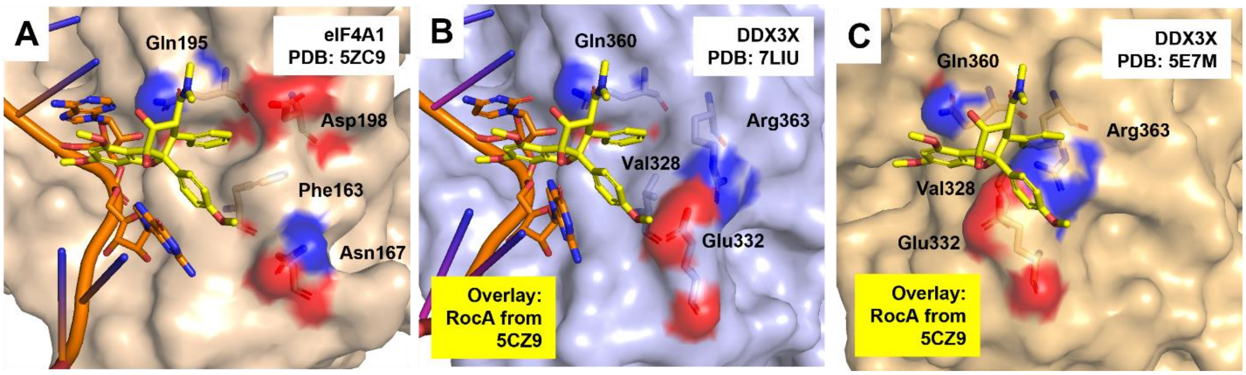

Figure S8. Comparative overlays of ligand RocA (-)-**1** in the rocaglate binding pocket of (**A**) the crystallographically-determined eIF4A-RNA-RocA complex (PDB: 5ZC9), (**B**) a DDX3X-DNA/RNA hybrid complex (PDB: 7LIU), and (**C**) a non-oligo-bound DDX3X structure (PDB: 5E7M). Overlay analysis reveals varying degrees of steric clashes between the RocA B-ring and Glu332 in both DDX3X structures (B-C), as well as significant C-ring clashes with DDX3X Arg363 in the 5E7M structure (C). The 7LIU structure was selected for rigid receptor and induced-fit modeling experiments based on this analysis.

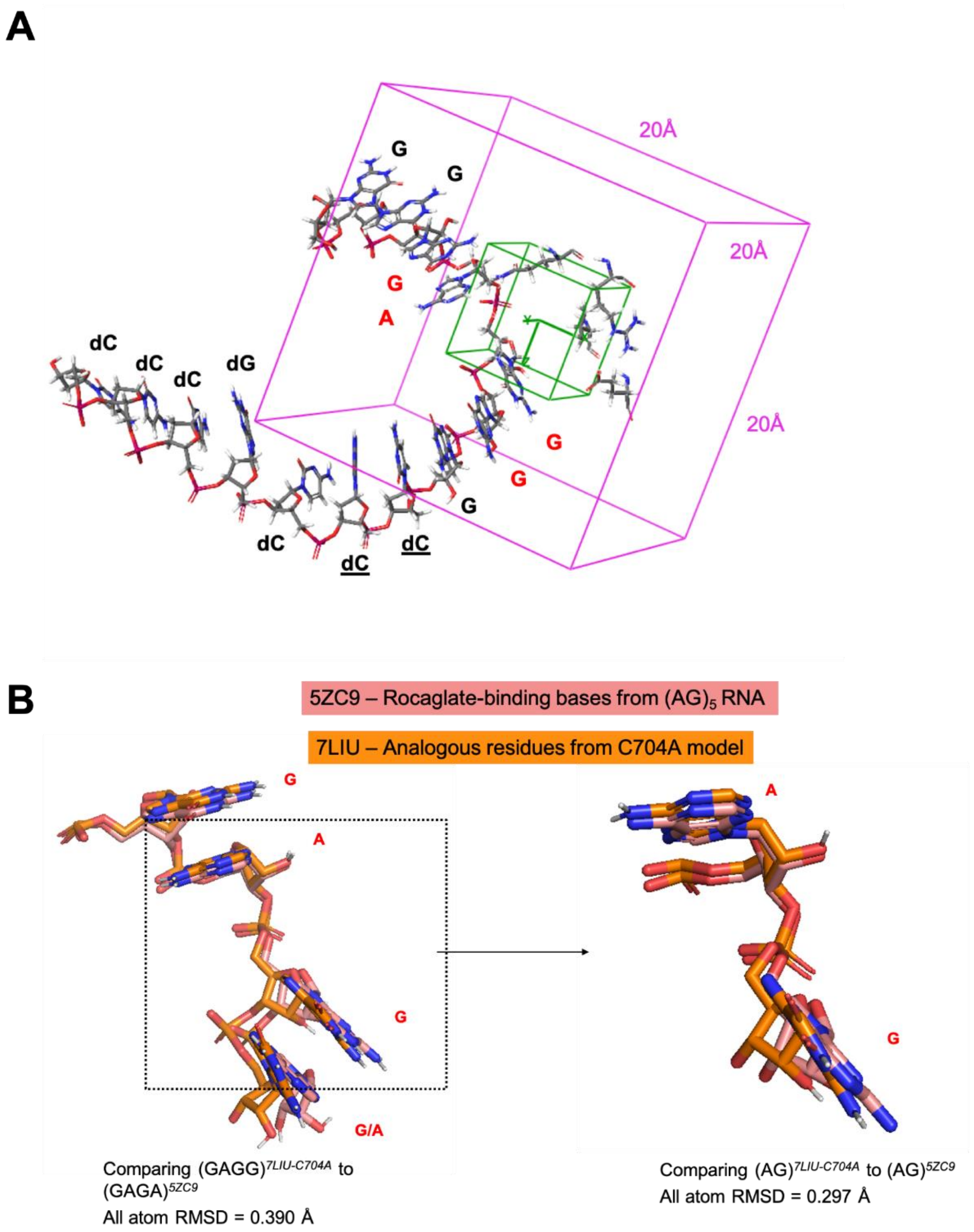

Figure S9. Analysis of the 7LIU-C704A receptor used for rocaglate-DDX3X modeling experiments. (**A**) In the modified model, all residues within the docking grid are purines (GGGAGGG). The residues highlighted in red flank the rocaglate binding site and are consistent with the enriched tetramer motifs identified by a Bind-n-Seq experiment with RocA and DDX3X.[S1] (**B**). Superposition of (GAGG) from the 7LIU-C704A model and (GAGA) from a RocA-eIF4A1-poly(AG) complex (PDB: 5ZC9) shows excellent overlap with low root mean square deviations (RMSD), suggesting an RNA conformation competent for rocaglate binding.

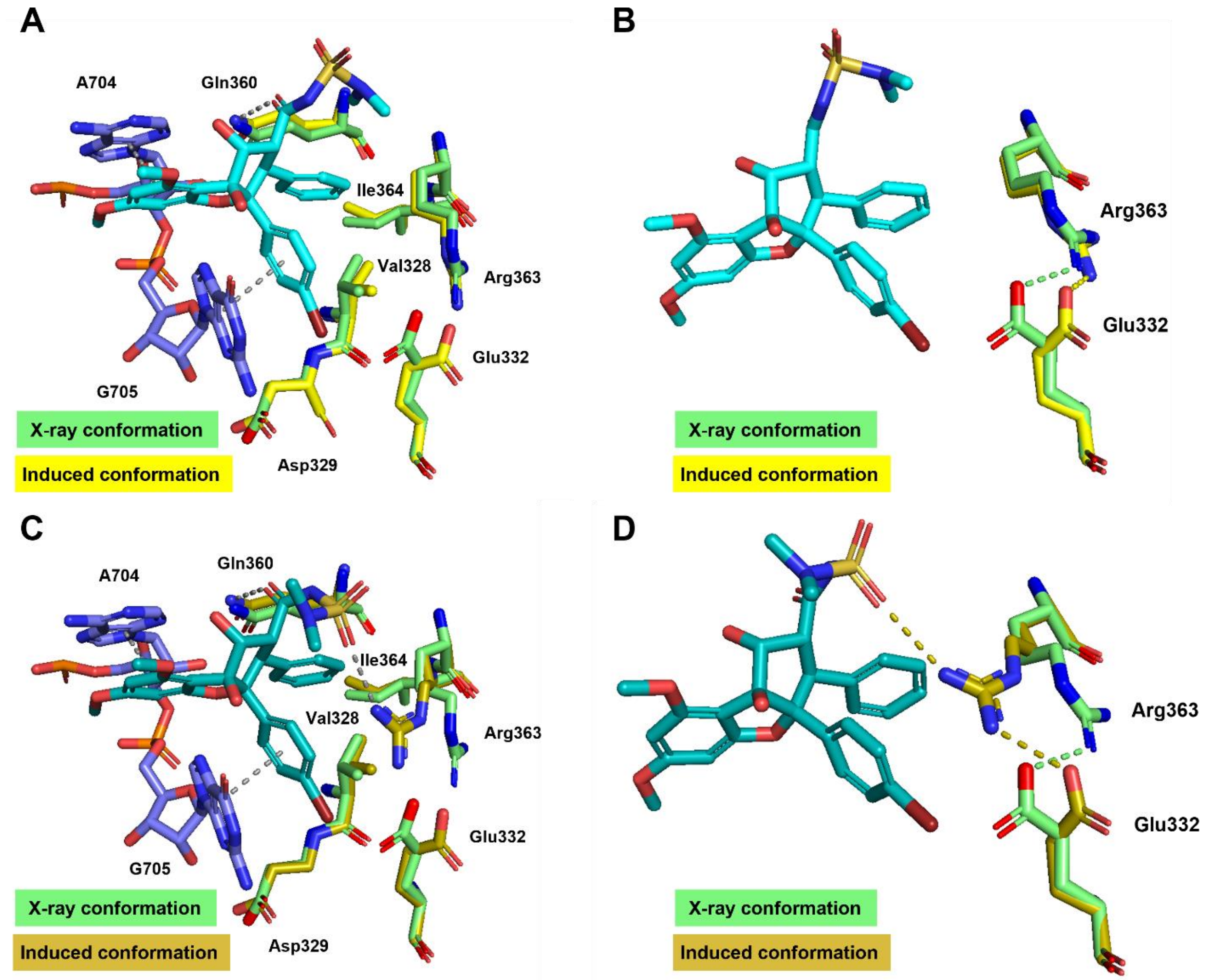

Figure S10. Comparison of induced fit docking (IFD) poses for (-)-**20** and DDX3X to the parent DDX3X X-ray structure (PDB: 7LIU). (**A**) View of the top-scored IFD pose comparing the ligand-accommodating adjustments for binding site residues between the parent X-ray (*lime*) and the induced-fit receptor (*yellow*). C704A-mutated RNA (*lavender*) was held rigid in the induced fit docking. (**B**) Adjusted view of (A) highlighting the rotation of Glu332, to avoid steric clash with the *C*4′ bromine substituent while maintaining a salt bridge to Arg363. (**C**) View of the second-ranked IFD pose comparing the ligand-accommodating adjustments for binding site residues between the parent X-ray (*lime*) and the induced-fit receptor (*olive*). C704A-mutated RNA (*lavender*) was held rigid in the induced fit docking. (**D**) Close-up view of (C) highlighting the rotation of Glu332 to avoid steric clash with the *C*4′ bromine substituent, rotation of the Arg363 sidechain to engage in a hydrogen bond with (-)-**20**, and the Glu332-Arg363 salt bridge interaction.

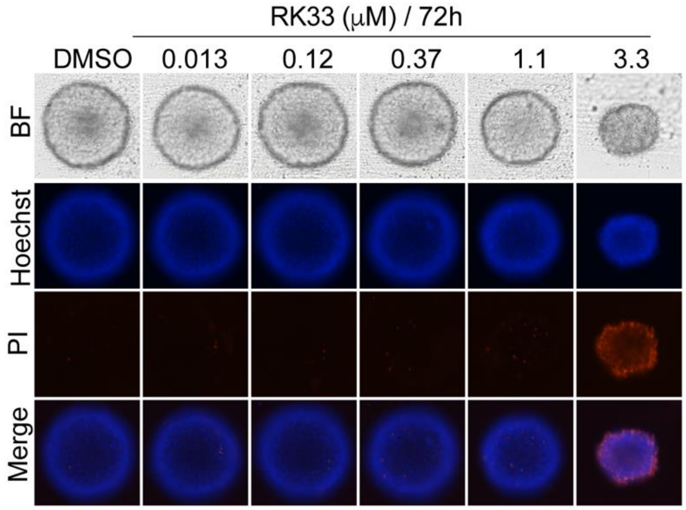

Figure S11. Propidium iodide (PI) and Hoechst staining of GBM neurospheres treated with varying doses of **RK-33** for 72 hours.

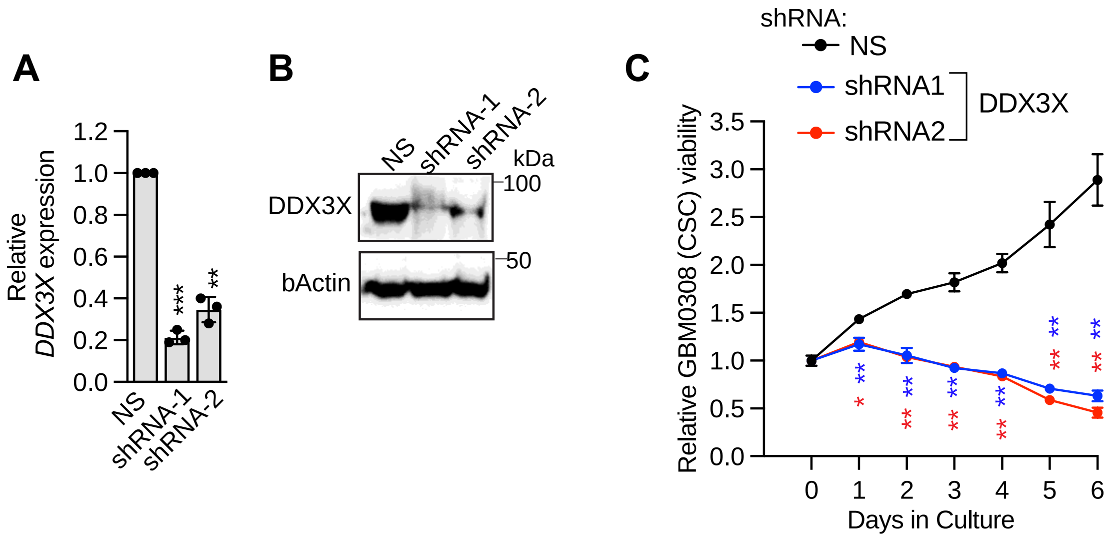

Figure S12. DDX3X knockdown in GBM0308 (CSCs) and its effects on proliferation. (**A**) QRT PCR and (**B**) immunoblot monitoring DDX3X mRNA and protein levels in GBM0308 cells expressing either non-silencing (NS) or the two independent DDX3X shRNAs (shRNA-1 and shRNA-2). (**C**) Cell viability assay in GBM0308 (CSCs) after shRNA-mediated knockdown of DDX3X. GBM0308 cells were transduced with two independent DDX3X shRNA lentiviruses. 12 hours post-transduction, cells were subjected to puromycin selection for 2 days. After selection, an equal number of cells expressing either non-specific (NS) shRNA or the two independent DDX3X shRNAs were seeded in a 96-well plate in triplicates. Cell viability was measured from day 1 to day 7 using a PrestoBlue reagent. Data are presented as mean ± SD. *P* values were calculated using Welch's t-test for (A) and Two-way ANOVA for (C). **P<0.05*, ***P<0.01*, ****P<0.001*.

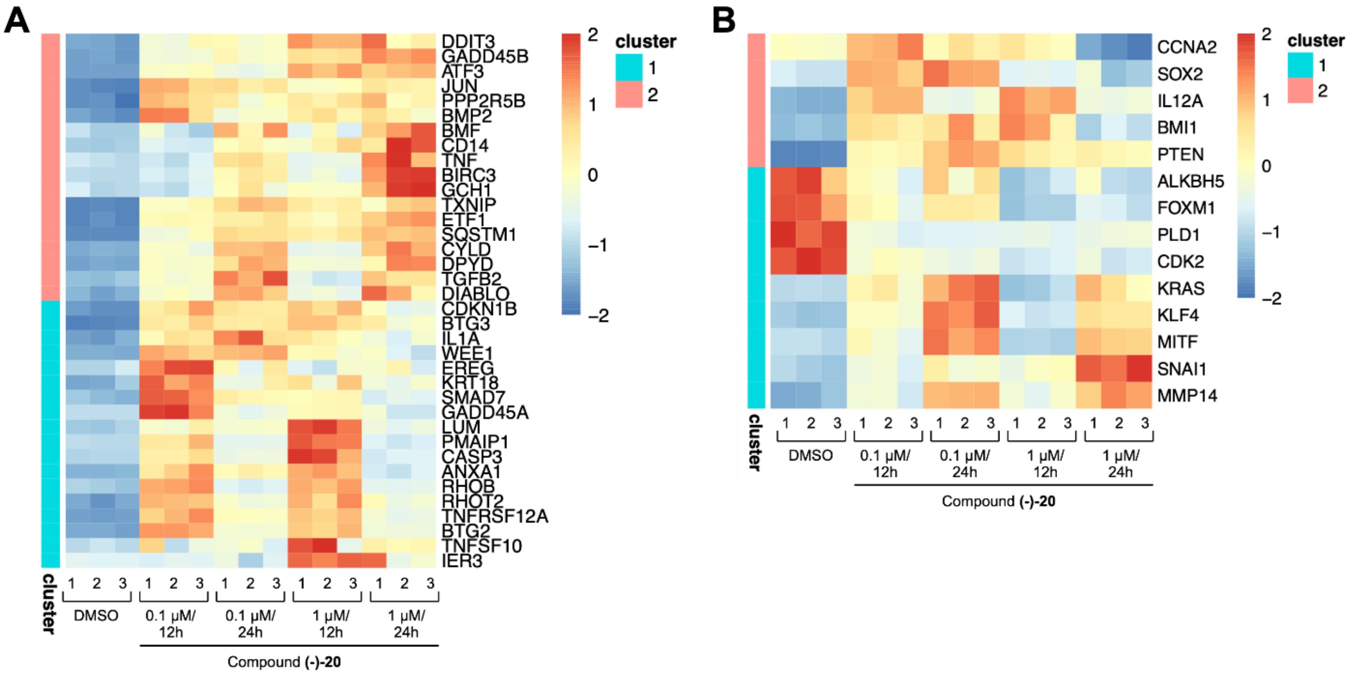

Figure S13. Z-score heatmap showing differentially expressed gene (DEG) signatures in GBM0308 stem cells treated with either DMSO or compound (-)-**20** for the indicated time and concentration. Only significant genes (FDR < 0.05 and |LFC| > 0.585) are presented here. Hierarchical clustering was performed using the complete linkage method and 1-Pearson as the distance. The genes were then classified into two clusters based on the dendrogram. (**A**) Heatmap visualizing significantly enriched DEGs in apoptotic pathways and (**B**) downstream of DDX3X.

II. Supplemental Methods for Biology and Proteomics

PISA assay in GBM0308 cell lysates

The PISA assay was performed in lysates as described by Gaetani and coworkers [S2] with slight modification. Pelleted GBM0308 cells were resuspended in ice-cold lysis buffer (1X PBS pH 7.4, 2 mM $MgCl_2$, 0.5 mM AMP-PNP, 1 μM $(AG)_8$ RNA, HALT protease inhibitor cocktail EDTA-free) and subjected to three freeze-thaw cycles with liquid nitrogen to extract lysate proteins. Thawing was performed at room temperature until ~80% thawed, then completed on ice. Lysate was clarified by centrifugation for 10 minutes at 20,000 RCF and 4 ºC, transferred to a fresh Eppendorf tube, then centrifuged again for 10 minutes at 20,000 RCF and 4 °C to ensure removal of debris. Lysate concentration was determined using a Pierce BCA Protein Assay Kit and adjusted to 2.0 mg/mL in lysis buffer. Lysate was mixed 1:1 with a 2X solution of test compound or DMSO in lysis buffer, gently aspirated to ensure proper mixing, and incubated at room temperature for 10 minutes. After incubation, lysate was aliquoted across a 96-well PCR plate (25 μL per well) and heated for 3 minutes in an Applied Biosystems ProFlex PCR System with six temperature zones (52, 54.4, 56.8, 59.2, 61.6, 64 ºC). After heating, the PCR plate was placed on ice and an equal volume of lysate (20 μL) was used to pool lysate from each of six temperature points back into one "integrated" sample per replicate. Precipitated protein aggregates were pelleted by centrifugation at 30,000 RCF and 4 ºC for 30 minutes. 20 μL of

supernatant (~10 μg protein) was transferred to fresh tubes containing 2 μL of Benzonase solution (~30 units) and incubated on ice for 30 minutes. Finally, samples were mixed with 11 μL of 3X SP3 sample preparation buffer (600 mM HEPES pH 8.5, 1.5% SDS, 30 mM TCEP, 120 mM chloroacetamide) and heated to 95 ºC for 10 minutes to denature, reduce, and alkylate proteins. Samples were then processed using solid-phase-enhanced sample-preparation (SP3).

Proteomics Sample Preparation

For SP3-based sample cleanup, [S3, 4] samples were mixed with Sera-Mag carboxylate-modified magnetic beads (10:1 bead to protein ratio by mass) and proteins precipitated onto beads with a 4X volume of ethanol. After 20 minutes of incubation in a ThermoMixer (24 ºC, 1000 RPM), samples were centrifuged for 1 minute at 500 RCF and beads pelleted against a magnet for 2 minutes. Upon removal of liquid, beads were washed 3X with 80% ethanol and 1X with 100% acetonitrile, briefly dried by SpeedVac, and resuspended in 50 μL of digestion buffer (100 mM HEPES pH 8.5, 1 mM $CaCl_2$, 0.01 μg/μL Trypsin/Lys-C mix). Proteins were digested on-bead in a ThermoMixer set to 37 ºC for 18 h with shaking. After digestion, samples containing beads were evaporated to dryness, resuspended in LC-MS grade water, then reacted with ~65 μg of TMTpro reagents (final reaction volume 20 μL, 30% acetonitrile) for 1 h. Samples were quenched with hydroxylamine, pooled, and evaporated to dryness in a SpeedVac. The pooled sample was resuspended in 2% acetonitrile, acidified with formic acid, and desalted using a Pierce Peptide Desalting Spin Column. The eluate was again evaporated to dryness in a SpeedVac prior to high pH reversed-phase peptide fractionation.

High pH Reversed-Phase Peptide Fractionation

TMTpro-labeled peptides were fractionated using high pH reversed-phase chromatography on an Agilent 1260 Infinity Capillary LC equipped with an XBridge Peptide BEH C18 column (300Å, 3.5 µm, 1 mm x 150 mm, Waters Corporation). Prior to fractionation, the column was flushed with 100% methanol containing 0.1% formic acid for 1 hour. Peptides were resuspended in 2% acetonitrile, 0.1% NH_4OH in water, injected manually, and separated using a gradient of mobile phase A (2% acetonitrile, 0.1% NH_4OH in water) to mobile phase B (2% water, 0.1% NH_4OH in acetonitrile) at a flow rate of 75 µL/min. Separation conditions were 100% mobile phase A for 5 minutes, a linear gradient to 12% mobile phase B for 5 minutes, a linear gradient to 32% mobile phase B for 35 minutes, a linear gradient to 45% mobile phase B for 8 minutes, a linear gradient to 70% mobile phase B for 1 minute, and a wash with 70% mobile phase B for 16 minutes (70 minutes total). Eluate fractions were collected from 19 to 67 minutes in 30 second time slices for a total of 96 fractions. Fractions were then concatenated into 24 pooled fractions and evaporated to dryness in a SpeedVac.

LC-MS/MS Data Acquisition and Database Searching

Pooled fractions were resuspended in 2% acetonitrile, 0.1% formic acid in water at ~0.1 µg/µL, and ~0.5 µg of peptides were separated on an Easy-nLC 1200 system connected to an Orbitrap Eclipse mass spectrometer equipped with a FAIMS Pro Interface. Mobile phase A consisted of 2% acetonitrile, 0.1% formic acid in water, and mobile phase B consisted of 80% acetonitrile, 0.1% formic acid in water. Peptides were first loaded onto an Acclaim PepMap C18 nano-trap column (100Å, 3 µm, 75 µm x 2 cm, Thermo Scientific) in mobile phase A, then separated on an EASY-Spray column (100Å, 2 µm, 75 µm x 500 mm, Thermo ES903, Scientific). Flow rate was set to 250 nL/min. Separation conditions were a linear gradient from 2% to 6% mobile phase B

over five minutes, a linear gradient to 30% mobile phase B for 112 minutes, a linear gradient to 40% mobile phase B for seven minutes, a linear gradient to 100% mobile phase B for four minutes, and a hold at 100% mobile phase B for six minutes before re-equilibration. The mass spectrometer was operated in positive ion mode with a spray voltage of 2500V and a capillary temperature of 275 °C. Data dependent acquisition was performed in cycle time mode with 3 FAIMS compensation voltages (-35, -50, -65) and a cycle time of 1 second per compensation voltage. Precursor ion scans were acquired in the Orbitrap with a resolution of 60,000, a normalized AGC target of 100%, maximum injection time set to automatic (50 ms), and a scan range of 400-1600 m/z. Additional filters included MIPS mode, a 60 second dynamic exclusion window, an intensity threshold of $4e^4$, and charge states of 2-6 for MS2. For MS2 scans, data were acquired in the Orbitrap with a resolution of 50,000, a normalized AGC target of 200%, maximum injection time set to automatic (86 ms), and HCD collision energy set to 35%.

MS/MS spectra were searched using the Andromeda search engine [S5] in MaxQuant software (version 2.0.3.0) [S6] against the UniProt Human complete FASTA database (downloaded February 8, 2023) and the MaxQuant contaminants database. Searches were performed using default MaxQuant settings with minor modification. Briefly, reporter ion MS2 mode was used with internal and terminal TMTpro 16plex labels corrected according to manufacturer-supplied correction values. Searches allowed for methionine oxidation and N-terminal protein acetylation as variable modifications, while cysteine carbamidomethylation was set as a fixed modification. Enzyme specificity was set to Trypsin/P with a maximum of two missed cleavages allowed. Precursor ions were searched with a mass tolerance set to 4.5 ppm and fragment ions were searched with a mass tolerance set to 20 ppm. Peptide spectrum match and protein FDRs were set at 0.01 using the target-decoy database search strategy.[S7] The minimum score for modified peptides was left at 40. Only unique peptides were used for quantification.

Proteomics Data Analysis

Additional data filtering and statistical analysis were conducted using R: *A language and environment for Statistical Computing* (R Foundation for Statistical Computing, http://www.R-project.org) using the Omics Notebook analysis pipeline. [S8] The MaxQuant output file designated "proteinGroups" provided a table of protein intensities that, after removal of contaminants and reverse hits, was log transformed and normalized. Group comparisons were conducted using moderated t-tests with a Benjamini-Hochberg correction to contain the false discovery rate at 1%. Only proteins that were quantified with at least 2 unique peptides were included in analyses. Volcano plots were built using the R package ggplot2 and dot plots were generated in Prism (version 9.3.1).

Fluorescence Polarization (FP) Assays

Recombinant DDX3X or eIF4A1 (1.5 μM) were incubated with 10 nM FAM-labelled RNA probe and test compounds for 10 min in FP buffer (14.4 mM HEPES- KOH (pH 8), 108 mM NaCl, 1 mM $MgCl_2$, 14.4% glycerol, 0.1% DMSO, 2 mM DTT, 1 mM ATP) at room temperature in black, low-volume 384 well plates (Corning 3820). FP readings were performed using a Pherastar FS microplate reader (BMG Labtech). Compounds were tested at a final concentration of 10 μM. [S9] For ADP+P_i conditions, ATP was substituted with 1 mM ADP and 1 mM Na_2HPO_4. To assess potency of complex formation, proteins were titrated in the presence of 10 nM probe, 50 μM compounds and 1 mM ATP. Experiments were run in three replicates, with each replicate employing the same master mix of protein, probe and buffer split across the four

test conditions (DMSO + three test compounds). Within each replicate, data was normalized such that the highest response across the four tested conditions (as raw ΔmP counts) was used as 100% relative change in polarization, and the lowest response across the four tested conditions was used as 0% relative change in polarization. Data was analyzed using GraphPad Prism 8.4.0, with a “[Agonist] vs. normalized response” nonlinear regression model used to determine the EC_{50} for stimulation of helicase-RNA probe binding in the presence of test compound.

***In vitro* translation assays**
In vitro translations were performed using 4 ng/μL of bicistronic FF-HCV-Ren/pA51 reporter mRNA and the indicated compound concentrations in Krebs-2 extracts at 30 °C for 1 h as described previously.[S10] Cap-dependent FLuc and cap-independent RLuc activities were assessed on a Berthold Lumat LB 9507 luminometer (Berthold Technologies). Curves were determined using a non-linear regression model on GraphPad Prism 8.4.0.

Lentivirus packaging, transduction, and shRNA knockdown
All shRNA clones were obtained from Open Biosystems/Thermo Scientific through the UMMS RNAi Core Facility. For packaging lentiviral shRNAs, 1 x 10^6 HEK293T cells were transfected with shRNA vectors expressing non-silencing (NS) or DDX3X shRNAs (shRNA1: TRCN0000000003 shRNA2: TRCN0000000004) along with packaging plasmids psPAX2 and pMD2.G in a 2:2:1 ratio using Effectene transfection reagent (QIAGEN, cat# 301425). The next day, the medium was replaced to remove DNA complexes, and a fresh NBE medium was added. Forty-eight hours post-transfection, medium containing lentiviral particles were collected and filtered through a 0.45 μm filter. For shRNA knockdown, 2 x 10^6 GBM0308 cells per well were seeded in 6-well plates and transduced with 750 µL lentivirus particles in a total volume of 1 mL of NBE medium supplemented with 8 µg/mL polybrene (Sigma, Cat#1003). The medium was replaced after overnight incubation to remove polybrene and viral particles, and cells were then subjected to puromycin selection (1 µg/mL) for 2 days. The knockdown of DDX3X was confirmed by qRT-PCR, and immunoblotting with DDX3X antibody.

Quantitative RT-PCR
Total RNA was extracted from cells using Trizol (Invitrogen, Cat# 15596). cDNA was synthesized using Proto Script II reverse transcription kit (NEB, Cat# E6560) and real-time PCR reactions were performed using Quant Studio 3 (Applied Biosystems by Thermo Scientific) using primer sequences given below. Expression of *DDX3X* was normalized to that of *GAPDH*.

DDX3-Forward	5′-AGCAGTTTTGGATCTCGTAGTG-3′
DDX3-Reverse	5′-ACTGTTTCCACCACGTTCAAAT-3′
GAPDH-Forward	5′-GTCTCCTCTGACTTCAACAGCG-3′
GAPDH-Reverse	5′-ACCACCCTGTTGCTGTAGCCAA-3′

Immunoblot Analysis
GBM0308 cells were lysed in RIPA buffer (50 mM Tris-HCl pH 7.4, 1% NP-40, 0.25% sodium deoxycholate, 150 mM NaCl, 1 mM EDTA) containing 1X protease inhibitor cocktail (Roche) and 1 mM PMSF. Total cell lysates (50 µg) were subjected to SDS-PAGE and transferred to nitrocellulose membrane, which were blocked with 5% non-fat dry milk, and incubated with anti-DDX3 (1:1000 dilution; Cell Signaling, Cat#2635), or anti-β-actin (1:2000 dilution; Sigma)

antibodies overnight at 4 °C. Rabbit and mouse HRP-conjugated secondary antibodies (Jackson Laboratories) were used for detection with ECL western blotting substrate (Thermofisher Scientific). Protein bands were detected and analyzed using ChemiDoc image analyzer (Bio-Rad).

Cell Proliferation Assay

GBM0308 cells were transduced with a lentivirus expressing non-silencing (NS) or DDX3X shRNA, cells were selected with puromycin for 2 days. The selected DDX3X KD cells and NS cells were seeded at a density of 3000 cells per well in 96 well plates. Cell proliferation was monitored for 7 days. Cell viability was assessed using PrestoBlue Cell Viability Reagent (Invitrogen™, Cat#A13261) as per the manufacturer's instructions.

RNA Sequencing and Analysis

GBM0308 cells cultured in NBE medium were treated with compound (-)-**20** at concentrations of 0.1 µM, 1 µM and DMSO. At 12 h and 24 h timepoints, total RNA was extracted using RNeasy mini kit (Qiagen) as per the manufacturer's instruction. Preparation of the RNA library and transcriptome sequencing were conducted by Novogene. RNASeq data analysis was performed with OneStopRNAseq.[S11] Paired-end reads were aligned to human genome hg38, with star_2.5.3a,[S12] annotated with GENCODE GRCh38.p12 annotation release 34.[S13] Aligned exon fragments with mapping quality higher than 20 were counted toward gene expression with featureCounts_1.5.2.[S14] Differential expression (DE) analysis was performed with DESeq2_1.20.0.[S15] Within DE analysis, 'ashr' was used to create log2 Fold Change (LFC) shrinkage for each comparison.[S16] Gene set enrichment analysis were performed with GSEA.[S17] GSEA analysis were performed on ranked gene list based on both LFC and p-value from the DE analysis (LFC-GSEA and pvalue-GSEA).

III. Computational Methods

Ligand Preparation

Compound (-)-**20** was prepared for docking using Schrödinger's LigPrep in the Maestro software environment (Version 14.0.134, Release 2024-2). During ligand preparation, Epik was used to predict possible protonation states in the pH range of 7.4 +/- 2.0. LigPrep produced two predicted protonation states for docking: the neutral species, and *C*2 acylsulfamide anion negatively charged at the amide oxygen.

DDX3X Receptor Preparation

The "7LIU-C704A" receptor was prepared from PDB X-ray structure 7LIU, point mutated to adenosine at residue C704 using Pymol (Version 2.4.0, Schrödinger LLC). The resulting structure was prepared for docking using the default Protein Preparation Workflow in the Maestro Software environment (Version 14.0.134, Release 2024-2), which involved structure pre-processing, hydrogen-bond optimization, restrained minimization (S-OPLS force field, hydrogen atoms freely minimized and heavy atoms minimized to r.m.s.d. 0.3), and removal of waters >4 Å from heteroatoms.

Glide Rigid Receptor Docking

Glide rigid receptor docking was performed in Schrödinger's Maestro software environment (Version 14.0.134, Release 2024-2). EIF4A1 X-ray structure (PDB: 5ZC9) was prepared for use as the docking receptor through default Protein Preparation Workflow, which involved structure preprocessing, hydrogen-bond optimization, restrained minimization (S-OPLS force field, hydrogen atoms freely minimized and heavy atoms minimized to r.m.s.d. 0.3), and removing water >4 Å from heteroatoms. To define the binding site, compound (-)-**20** was confined to the centroid of Workspace ligand (i.e. RocA) during receptor grid generation. The highest-scored docking pose was selected for further analysis.

Induced Fit Docking (IFD)

Induced Fit Docking was performed in Schrödinger's Maestro software environment (Version 14.0.134, Release 2024-2). To define the binding site, the center-of-mass of each docked ligand was restrained to a 10 Å box positioned at the centroid of the following residues: Val328, Glu332, Gln360, Arg363 (from DDX3X), A704 and G705 (from the DNA-RNA hybrid oligonucleotide). During Induced Fit Docking, all protein sidechains within 5Å of the docked ligand were designated flexible, while all oligonucleotide sidechains were designated as rigid. Of note, the oxygen-anion input ligand produced by LigPrep exclusively produced output IFD poses bearing a negative charge at nitrogen. The top-scored and the second-scored IFD poses were both obtained for the compound (-)-**20** nitrogen anion (docking score -6.986 kcal/mol and -6.131, respectively) and were selected for analysis.

IV. General Methods for Chemical Synthesis

Unless otherwise stated, ^{1}H NMR and ^{13}C NMR spectra were obtained in $CDCl_3$, CD_3OD (Cambridge Isotope Laboratories, Inc.) using a Varian Innova-400 MHz 500 MHz spectrometer. Chemical shifts are reported in parts per million relative to the internal solvent peak ($CDCl_3$: δ 7.26 for ^{1}H; δ 77.16 for ^{13}C. CD_3OD: δ 3.31 for ^{1}H; δ 49.00 for ^{13}C). Data for ^{1}H NMR are reported as follows: chemical shift, multiplicity (br = broad, s = singlet, d = doublet, t = triplet, q = quartet, m = multiplet), coupling constants, and integration. All ^{13}C NMR spectra were recorded with complete proton decoupling. Infrared (IR) spectra were recorded on a Bruker ALPHA P FT-IR spectrometer equipped with a diamond ATR module. High resolution mass spectra (HRMS) (ESI) were obtained using a Waters Q-TOF mass spectrometer at the Boston University Chemical Instrumentation Center, or by DART at the Boston College Mass Spectrometry Center. Melting points were recorded on a Mel-temp apparatus (Laboratory Devices) and are uncorrected. Analytical LC-MS and chiral HPLC analysis was performed on a Waters Acquity UPC^2 system (Waters MassLynx Version 4.2) with a binary solvent manager (Supercritical CO_2 and MeOH), a QDa mass spectrometer, a Waters PDA (PhotoDiode Array) detector, and an ELSD (Evaporative Light Scattering Detector). Optical rotations were measured on Rudolph Autopol II at 589 nm, and specific rotations are given $[\alpha]_D$. All reactions were carried out in oven-dried glassware under an argon/nitrogen atmosphere unless otherwise noted. Analytical thin layer chromatography (TLC) was conducted using 0.25 mm silica gel F-254 plates (SiliCycle). Flash chromatography was conducted using silica gel (SiliaFlash P60, particle size 40 – 63 µm) purchased from SiliCycle.

Materials: Chemicals were purchased from Sigma-Aldrich, Fisher, Alfa Aesar, TCI, Oakwood, Enamine, and ChemScene and were used without further purification.

V. Chemical Synthesis Procedures and Compound Characterization

General method for synthesis of rocaglate ß-lactones
Synthesis of rocaglate ß-lactones using previously reported conditions

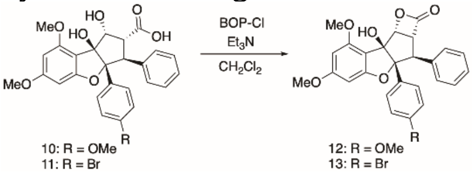

To a dry 4 mL vial was added rocaglaic acid **10** or **11**, a stir bar and dry dichloromethane under argon, and the resulting solution was cooled to 0 ºC before adding triethylamine (3.0 equiv.) and *bis*(2-oxo-3-oxazolidinyl)phosphinic chloride (BOP-Cl, 1.3 equiv.). The reaction mixture was warmed to room temperature while stirring for 3 h. Water (2 mL) and CH_2Cl_2 (2 mL) were added after the reaction was complete and the aqueous layer was further extracted with CH_2Cl_2 (3 X 10 mL). The combined organic layers were washed with water and brine before being dried over sodium sulfate, filtered, and concentrated *in vacuo* to afford a yellow foam. The crude yellow solid was purified using silica gel column chromatography (35:65 EtOAc/hexanes) to afford ß-lactone **12** or **13** (50%, 67%, respectively) as a white solid. Characterization data for **12** agreed with the literature report. [S18]

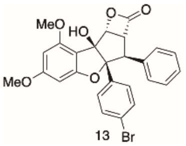

(2a*R*,3*S*,3a*R*,8b*S*,8c*R*)-3a-(4-bromophenyl)-8b-hydroxy-6,8-dimethoxy-3-phenyl-3,3a,8b,8c-tetrahydrooxeto[2',3':3,4]cyclopenta[1,2-*b*]benzofuran-2(2a*H*)-one (13)

^{1}H NMR (500 MHz, $CDCl_3$) δ 7.21 (d, *J* = 1.9 Hz, 1H), 7.20 (d, *J* = 1.9 Hz, 1H), 7.17 (dd, *J* = 4.9, 1.8 Hz, 3H), 7.00 (d, *J* = 1.9 Hz, 1H), 6.99 (d, *J* = 1.9 Hz, 1H), 6.96 – 6.92 (m, 2H), 6.23 (d, *J* = 1.9 Hz, 1H), 6.08 (d, *J* = 1.9 Hz, 1H), 5.40 (d, *J* = 5.0 Hz, 1H), 4.57 (t, *J* = 4.9 Hz, 1H), 4.13 – 4.10 (m, 1H), 3.81 (s, 3H), 3.80 (s, 3H) ppm. ^{13}C NMR (126 MHz, $CDCl_3$) δ 169.0, 164.1, 159.6, 157.3, 136.7, 134.1, 130.4, 129.3, 128.7, 128.3, 127.5, 122.1, 107.5, 106.4, 93.0, 89.9, 89.8, 82.9, 60.7, 55.7, 55.6, 54.6 ppm. IR υ_{max}: 3498, 2927, 2847, 1832, 1601, 1501, 1454, 1218, 1200, 1148, 1126, 1073, 817, 735 cm^{-1}. M.p. : 140-145 ºC (EtOAc:hexanes). TLC R_f = 0.3 (Eluent: EtOAc:hexanes = 35:65). HRMS (DART): m/z calculated for $[C_{25}H_{21}BrO_6 + H]^+$ 509.0594, found 509.0590.

General method for the synthesis of rocaglaic acid derivatives through ring-opening of rocaglate ß-lactones:

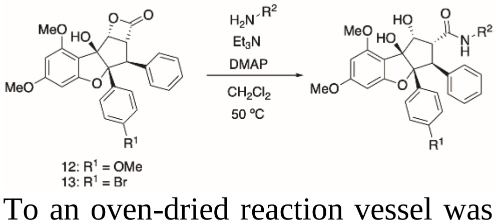

To an oven-dried reaction vessel was added rocaglate ß-lactone **12** or **13**, a stir bar, anhydrous CH_2Cl_2 (0.1 M), triethylamine (3 equiv.), primary amine (2 equiv.), and 4-dimethylaminopyridine (DMAP, 1 equiv.) at room temperature. After stirring the reaction mixture at 50 ºC for 18 h, 1M HCl was added to the solution until the aqueous layer reached a pH of ~2. The organic phase was separated before the aqueous layer was further extracted with CH_2Cl_2 (3 x 10 mL). The combined organic layers were washed sequentially with water and

brine, dried over sodium sulfate, filtered, and concentrated *in vacuo* to afford the crude product mixture as a yellow foam. Flash chromatography or preparative TLC was utilized to obtain pure rocaglate derivatives as white solids (note: the corresponding yields employing various amines are shown in **Scheme 1**).

Chiral resolution of (±)- rocaglaic acids using (-)-quinine:
To a 20 mL vial was added (±)- rocaglaic acid, a stir bar, and ethanol (0.2 M), and to the resulting solution was added (-)-quinine (1 equiv.). The reaction was stirred at room temperature for 18 h. The solvent was removed *in vacuo* to afford a white solid before conducting recrystallization to separate the diastereomeric salts using acetone. The precipitated white solid (diastereomeric salts **22** or **24**) was collected by filtration, and the mother liquor was dried under vacuum to afford the diastereomeric salts **23** or **25**. The separated salts were suspended in ethyl acetate before adding 5% HCl and the biphasic mixture was stirred for 0.5 h at room temperature. The aqueous layer was further extracted with ethyl acetate and the combined organic fractions were washed with water and brine, dried over sodium sulfate, filtered, and concentrated *in vacuo* to afford the enantioenriched rocaglaic acid derivative.

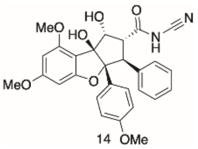

(1*R*,2*R*,3*S*,3a*R*,8b*S*)-*N*-cyano-1,8b-dihydroxy-6,8-dimethoxy-3a-(4-methoxyphenyl)-3-phenyl-2,3,3a,8b-tetrahydro-1*H*-cyclopenta[*b*]benzofuran-2-carboxamide (14)
^{1}H NMR (500 MHz, $CDCl_3$) δ 7.11 – 7.06 (m, 5H), 6.88 (t, *J* = 4.1 Hz, 2H), 6.67 (d, *J* = 8.2 Hz, 2H), 6.29 (d, *J* = 1.7 Hz, 1H), 6.15 (d, *J* = 1.8 Hz, 1H), 5.11 – 5.06 (m, 1H), 4.18 (d, *J* = 13.1 Hz, 1H), 4.13 – 3.93 (m, 1H), 3.89 (s, 3H), 3.84 (s, 3H), 3.71 (s, 3H) ppm. ^{13}C NMR (126 MHz, $CDCl_3$) δ 169.9, 164.4, 160.9, 159.0, 156.9, 135.0, 129.0, 128.2, 127.9, 127.2, 125.0, 112.8, 106.9, 106.6, 101.2, 93.4, 92.9, 89.6, 78.5, 56.4, 55.9, 55.8, 55.1, 50.8 ppm. IR υ_{max}: 3447, 3059, 2928, 2843, 2260, 1735, 1598, 1440, 1147, 1030, 869 cm^{-1}. M.p. : 170-175 ºC ($MeOH:CH_2Cl_2$). TLC R_f = 0.1 (Eluent: $MeOH:CH_2Cl_2$ = 5:95). HRMS (ESI): m/z calculated for $[C_{28}H_{26}N_2O_7 + Na]^+$ 525.1638, found 525.1624.

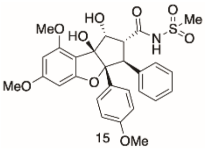

(1*R*,2*R*,3*S*,3a*R*,8b*S*)-1,8b-dihydroxy-6,8-dimethoxy-3a-(4-methoxyphenyl)-*N*-(methylsulfonyl)-3-phenyl-2,3,3a,8b-tetrahydro-1*H*-cyclopenta[*b*]benzofuran-2-carboxamide (15)
^{1}H NMR (500 MHz, $CDCl_3$) δ 7.11 – 7.04 (m, 5H), 6.90 (dd, *J* = 6.7, 3.0 Hz, 2H), 6.29 (d, *J* = 1.9 Hz, 1H), 6.14 (d, *J* = 2.0 Hz, 1H), 5.04 (d, *J* = 6.3 Hz, 1H), 4.18 (d, *J* = 13.2 Hz, 1H), 3.88 (s, 3H), 3.84 (s, 3H), 3.81 (dd, *J* = 13.6, 6.0 Hz, 1H), 3.70 (s, 3H), 3.02 (s, 3H) ppm.^{13}C NMR (126 MHz, $CDCl_3$) δ 164.4, 160.9, 158.9, 156.9, 135.3, 129.0, 128.2, 127.9, 126.6, 125.8, 112.4, 106.7, 101.3, 93.4, 92.8, 89.5, 78.8, 77.2, 56.1, 55.9, 55.8, 55.1, 52.0, 43.4, 40.9 ppm. IR υ_{max}: 3520, 3254, 2957, 2932, 2845, 1719, 1600, 1514, 1453, 1440, 1274, 1200, 1172, 1031, 869 cm^{-1}. M.p. : 135-140 ºC ($MeOH:CH_2Cl_2$). TLC R_f = 0.2 (Eluent: $MeOH:CH_2Cl_2$ = 5:95). HRMS (ESI): m/z calculated for $[C_{28}H_{29}NO_9S + Na]^+$ 598.1461, found 598.1441.

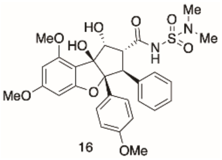

(1*R*,2*R*,3*S*,3a*R*,8b*S*)-*N*-(*N*,*N*-dimethylsulfamoyl)-1,8b-dihydroxy-6,8-dimethoxy-3a-(4-methoxyphenyl)-3-phenyl-2,3,3a,8b-tetrahydro-1*H*-cyclopenta[*b*]benzofuran-2-carboxamide (16)
^{1}H NMR (500 MHz, $CDCl_3$) δ 8.59 (s, 1H), 7.14 – 7.06 (m, 5H), 6.93 (dd, *J* = 6.9, 2.8 Hz, 2H), 6.69 – 6.65 (m, 2H), 6.30 (d, J = 1.9 Hz, 1H), 6.15 (d, *J* =

1.9 Hz, 1H), 4.97 (dd, *J* = 5.8, 1.7 Hz, 1H), 4.11 (d, *J* = 14.2 Hz, 1H), 3.90 (s, 3H), 3.85 (s, 3H), 3.78 (dd, *J* = 14.0, 5.1 Hz, 1H), 3.71 (s, 3H), 2.59 (s, 6H) ppm. ^{13}C NMR (126 MHz, $CDCl_3$) δ 168.6, 164.4, 160.8, 158.9, 156.9, 135.2, 129.1, 128.3, 127.9, 127.2, 125.7, 112.8, 106.8, 101.1, 93.6, 92.8, 89.5, 78.4, 56.8, 55.9, 55.8, 55.1, 52.4, 38.0 ppm. IR υ_{max}: 3481, 3251, 3059, 2960, 2924, 2850, 1724, 1623, 1514, 1454, 1199, 1147, 1015, 800 cm^{-1}. M.p. : 120-125 ºC ($MeOH:CH_2Cl_2$). TLC R_f = 0.2 (Eluent: $MeOH:CH_2Cl_2$ = 5:95). HRMS (ESI): m/z calculated for $[C_{29}H_{32}N_2O_9S + H]^+$ 585.1907, found 585.1927.

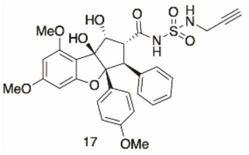

(1*R*,2*R*,3*S*,3a*R*,8b*S*)-1,8b-dihydroxy-6,8-dimethoxy-3a-(4-methoxyphenyl)-3-phenyl-*N*-(*N*-(prop-2-yn-1-yl)sulfamoyl)-2,3,3a,8b-tetrahydro-1*H*-cyclopenta[*b*]benzofuran-2-carboxamide (17)

^{1}H NMR (500 MHz, CD_3OD) δ 7.15 – 7.08 (m, 2H), 7.06 – 6.98 (m, 3H), 6.90 – 6.87 (m, 2H), 6.66 – 6.61 (m, 2H), 6.29 (d, *J* = 1.9 Hz, 1H), 6.18 (d, *J* = 2.0 Hz, 1H), 4.89 (s, 1H), 4.26 (d, *J* = 14.0 Hz, 1H), 3.90 – 3.86 (m, 1H), 3.85 (s, 3H), 3.82 (s, 3H), 3.73 – 3.68 (m, 1H), 3.66 (s, 4H), 3.66 – 3.62 (m, 1H), 2.54 (t, *J* = 2.6 Hz, 1H) ppm. ^{13}C NMR (126 MHz, CD_3OD) δ 168.9, 163.9, 160.7, 158.5, 157.8, 137.5, 128.8, 127.8, 127.7, 127.1, 125.9, 111.8, 107.9, 101.1, 93.8, 91.7, 79.0, 78.1, 72.1, 54.7, 54.6, 54.6, 54.0, 51.4, 32.1 ppm. IR υ_{max}: 3485, 3279, 2928, 2843, 1712, 1600, 1513, 1453, 1200, 1146, 1032, 827, 643 cm^{-1}. M.p. : 175-180 ºC ($MeOH:CH_2Cl_2$). TLC R_f = 0.1 (Eluent: $MeOH:CH_2Cl_2$ = 10:90). HRMS (ESI): m/z calculated for $[C_{30}H_{30}N_2O_9S + H]^+$ 595.1750 found 595.1750.

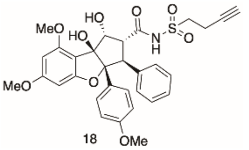

(1*R*,2*R*,3*S*,3a*R*,8b*S*)-*N*-(but-3-yn-1-ylsulfonyl)-1,8b-dihydroxy-6,8-dimethoxy-3a-(4-methoxyphenyl)-3-phenyl-2,3,3a,8b-tetrahydro-1*H*-cyclopenta[*b*]benzofuran-2-carboxamide (18)

^{1}H NMR (500 MHz, $CDCl_3$) δ 7.09 (dt, *J* = 7.0, 2.7 Hz, 5H), 6.92 – 6.85 (m, 2H), 6.71 – 6.64 (m, 2H), 6.30 (d, *J* = 1.9 Hz, 1H), 6.16 (d, *J* = 1.9 Hz, 1H), 5.03 (d, *J* = 6.2 Hz, 1H), 4.14 (d, *J* = 13.5 Hz, 1H), 3.80 (dd, *J* = 13.5, 6.2 Hz, 1H), 3.41 (t, *J* = 7.8 Hz, 2H), 2.53 – 2.31 (m, 2H), 1.94 (t, *J* = 2.7 Hz, 1H) ppm. ^{13}C NMR (126 MHz, $CDCl_3$) δ 169.2, 164.5, 160.8, 159.0, 156.8, 135.0, 129.0, 128.2, 128.0, 127.3, 125.6, 112.8, 106.7, 101.1, 93.4, 92.9, 89.6, 79.1, 78.7, 70.5, 56.3, 56.0, 55.8, 55.1, 52.2, 51.1, 13.4 ppm. IR υ_{max}: 3489, 3288, 3057, 2960, 2924, 2850, 1726, 1623, 1514, 1439, 1120, 1146, 1029, 869, 641 cm^{-1}. M.p. : 160-165 ºC ($MeOH:CH_2Cl_2$). TLC R_f = 0.1 (Eluent: $MeOH:CH_2Cl_2$ = 5:95). HRMS (ESI): m/z calculated for $[C_{31}H_{31}NO_9S + H]^+$ 594.1798, found 594.1788.

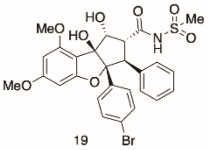

(1*R*,2*R*,3*S*,3a*R*,8b*S*)-3a-(4-bromophenyl)-1,8b-dihydroxy-6,8-dimethoxy-*N*-(methylsulfonyl)-3-phenyl-2,3,3a,8b-tetrahydro-1*H*-cyclopenta[*b*]benzofuran-2-carboxamide (19)

^{1}H NMR (500 MHz, CD_3OD) δ 7.20 (dd, *J* = 8.8, 2.3 Hz, 2H), 7.12 (dd, *J* = 8.8, 2.2 Hz, 2H), 7.04 (dtt, *J* = 13.8, 7.2, 3.5 Hz, 3H), 6.94 (d, *J* = 7.6 Hz, 2H), 6.29 (d, *J* = 2.1 Hz, 1H), 6.18 (d, *J* = 2.2 Hz, 1H), 4.60 (s, 1H), 4.35 (dd, *J* = 14.1, 2.2 Hz, 1H), 3.92 (ddd, *J* = 14.0, 6.3, 2.2 Hz, 1H), 3.84 (s, 3H), 3.82 (s, 3H), 3.09 (s, 3H) ppm. ^{13}C NMR (126 MHz, CD_3OD) δ 163.9, 160.6, 157.9, 137.3, 135.5, 129.5, 129.4, 127.6, 127.3, 126.1, 120.4, 107.5, 101.1, 93.9, 91.8, 88.5, 79.1, 70.1, 54.7, 54.6, 52.0, 39.5 ppm. IR υ_{max}: 3484, 3200, 2961, 2923, 2853, 1721, 1604, 1501, 1455, 1440, 1257, 1218, 1185, 1038, 869 cm^{-1}. M.p. : >200 ºC ($MeOH:CH_2Cl_2$). TLC R_f = 0.1 (Eluent: $MeOH:CH_2Cl_2$ = 10:90). HRMS (DART): m/z calculated for $[C_{27}H_{26}BrNO_8S + H]^+$ 604.0635, found 604.0631.

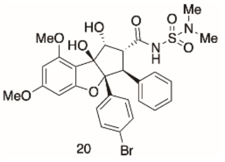

(1*R*,2*R*,3*S*,3a*R*,8b*S*)-3a-(4-bromophenyl)-*N*-(*N*,*N*-dimethylsulfamoyl)-1,8b-dihydroxy-6,8-dimethoxy-3-phenyl-2,3,3a,8b-tetrahydro-1*H*-cyclopenta[*b*]benzofuran-2-carboxamide (20)

^{1}H NMR (500 MHz, CD_3OD) δ 7.23 – 7.19 (m, 2H), 7.14 – 7.11 (m, 2H), 7.08 – 7.01 (m, 3H), 6.96 – 6.91 (m, 2H), 6.30 (d, *J* = 2.0 Hz, 1H), 6.18 (d, *J* = 1.9 Hz, 1H), 4.85 (d, *J* = 6.2 Hz, 1H), 4.33 (d, *J* = 14.1 Hz, 1H), 3.90 (dd, *J* = 14.1, 6.2 Hz, 1H), 3.84 (s, 3H), 3.82 (s, 3H), 2.76 (s, 6H) ppm. ^{13}C NMR (126 MHz, CD_3OD) δ 169.3, 163.9, 160.6, 157.9, 137.2, 135.4, 129.6, 129.4, 127.6, 127.3, 126.2, 120.4, 107.5, 101.0, 94.0, 91.8, 88.6, 78.9, 70.1, 54.8, 54.7, 54.6, 51.5, 37.2 ppm. IR υ_{max}: 3486, 3236, 2917, 2800, 1732, 1628, 1596, 1466, 1151, 1137, 1057, 808 cm^{-1}. M.p. : >200 ºC (MeOH:CH_2Cl_2). TLC R_f = 0.2 (Eluent: MeOH:CH_2Cl_2 = 10:90). HRMS (DART): m/z calculated for $[C_{28}H_{29}BrN_2O_8S + H]^+$ 633.0901, found 633.0905.

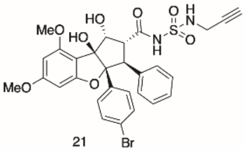

(1*R*,2*R*,3*S*,3a*R*,8b*S*)-3a-(4-bromophenyl)-1,8b-dihydroxy-6,8-dimethoxy-3-phenyl-*N*-(*N*-(prop-2-yn-1-yl)sulfamoyl)-2,3,3a,8b-tetrahydro-1*H*-cyclopenta[*b*]benzofuran-2-carboxamide (21)

^{1}H NMR (500 MHz, CD_3OD) δ 7.22 – 7.18 (m, 2H), 7.14 – 7.10 (m, 2H), 7.08 – 6.99 (m, 3H), 6.96 – 6.92 (m, 2H), 6.29 (d, *J* = 1.9 Hz, 1H), 6.18 (d, *J* = 2.0 Hz, 1H), 4.85 (d, *J* = 6.1 Hz, 1H), 4.61 (s, 1H), 4.35 (d, *J* = 14.0 Hz, 1H), 3.89 (dd, *J* = 14.0, 6.2 Hz, 1H), 3.84 (s, 3H), 3.82 (s, 3H), 3.73 – 3.63 (m, 2H), 2.56 (t, *J* = 2.5 Hz, 1H) ppm. ^{13}C NMR (126 MHz, CD_3OD) δ 168.1, 163.9, 160.7, 157.9, 137.3, 135.5, 129.6, 129.3, 127.8, 127.3, 126.1, 120.4, 107.5, 101.1, 93.9, 91.8, 88.5, 79.0, 78.3, 72.0, 54.9, 54.7, 54.6, 51.8, 32.2 ppm. IR υ_{max}: 3501, 3287, 2934, 2810, 1708, 1626, 1601, 1500, 1491, 1201, 1148, 1037, 816, 620 cm^{-1}. M.p. : 185-190 ºC (MeOH:CH_2Cl_2). TLC R_f = 0.2 (Eluent: MeOH:CH_2Cl_2 = 10:90). HRMS (DART): m/z calculated for $[C_{29}H_{27}BrN_2O_8S + H]^+$ 643.0744, found 643.0758.

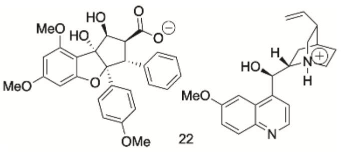

(1*S*,2*S*,4*S*,5*R*)-2-((*R*)-hydroxy(6-methoxyquinolin-4-yl)methyl)-5-vinylquinuclidin-1-ium (1*S*,2*S*,3*R*,3a*S*,8b*R*)-1,8b-dihydroxy-6,8-dimethoxy-3a-(4-methoxyphenyl)-3-phenyl-2,3,3a,8b-tetrahydro-1*H*-cyclopenta[*b*]benzofuran-2-carboxylate (22)

^{1}H NMR (500 MHz, CD_3OD) δ 8.60 (d, *J* = 4.6 Hz, 1H), 7.87 (d, *J* = 9.2 Hz, 1H), 7.69 (dd, *J* = 4.6, 0.8 Hz, 1H), 7.35 (dd, *J* = 9.2, 2.6 Hz, 1H), 7.30 (d, *J* = 2.7 Hz, 1H), 7.16 – 7.11 (m, 2H), 7.07 – 7.04 (m, 2H), 6.99 – 6.89 (m, 3H), 6.61 – 6.57 (m, 2H), 6.25 (d, *J* = 2.0 Hz, 1H), 6.09 (d, *J* = 2.0 Hz, 1H), 6.08 (s, 1H), 5.64 (ddd, *J* = 17.2, 10.4, 6.9 Hz, 1H), 5.00 (dt, *J* = 17.2, 1.3 Hz, 2H), 4.87 (d, *J* = 6.1 Hz, 1H), 4.25 (d, *J* = 14.2 Hz, 1H), 4.01 (tdd, *J* = 10.8, 4.9, 2.5 Hz, 1H), 3.86 (dd, *J* = 14.2, 6.1 Hz, 1H), 3.79 (s, 3H), 3.75 (s, 3H), 3.74 (s, 3H), 3.62 (s, 3H), 3.40 (ddd, *J* = 12.1, 6.0, 4.3 Hz, 1H), 3.22 (dd, *J* = 13.4, 10.6 Hz, 1H), 3.02 (ddd, *J* = 13.3, 5.3, 2.6 Hz, 1H), 2.95 – 2.86 (m, 1H), 2.58 (dtt, *J* = 12.1, 6.7, 2.1 Hz, 1H), 2.14 – 1.98 (m, 1H), 1.95 (q, *J* = 3.0 Hz, 1H), 1.75 (m, 1H), 1.35 (tt, *J* = 13.5, 3.3 Hz, 1H) ppm. ^{13}C NMR (126 MHz, CD_3OD) δ 177.6, 163.6, 160.9, 158.6, 158.3, 157.9, 146.5, 146.3, 143.1, 138.8, 138.4, 129.9, 128.8, 128.4, 128.1, 127.1, 126.1, 125.6, 122.2, 118.9, 115.2, 111.7, 108.4, 101.9, 100.9, 93.8, 91.5, 88.5, 79.5, 66.8, 59.5, 57.0, 55.5, 54.7, 54.5, 54.0, 53.9, 52.9, 43.4, 37.3, 27.0, 24.1, 18.0 ppm. M.p. : 175-180 ºC (MeOH:CH_2Cl_2). α_D^{19} = -9.94 (*c* = 0.1, MeOH).

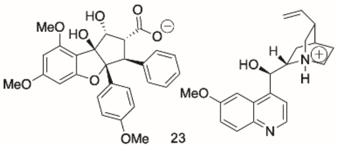

(1*S*,2*S*,4*S*,5*R*)-2-((*R*)-hydroxy(6-methoxyquinolin-4-yl)methyl)-5-vinylquinuclidin-1-ium (1*R*,2*R*,3*S*,3a*R*,8b*S*)-1,8b-dihydroxy-6,8-dimethoxy-3a-(4-methoxyphenyl)-3-phenyl-2,3,3a,8b-tetrahydro-1*H*-cyclopenta[*b*]benzofuran-2-carboxylate (23)

^{1}H NMR (500 MHz, CD_3OD) δ 8.58 (d, *J* = 4.6 Hz, 2H), 7.86 (d, *J* = 9.2 Hz, 1H), 7.68 (d, *J* = 4.6 Hz, 2H), 7.36 (d, *J* = 2.7 Hz, 2H), 7.32 (dd, *J* = 9.2, 2.6 Hz, 1H), 7.18 – 7.12 (m, 2H), 7.09 – 7.05 (m, 2H), 7.00 – 6.89 (m, 3H), 6.61 – 6.57 (m, 2H), 6.27 (d, *J* = 2.0 Hz, 1H), 6.16 (d, *J* = 2.0 Hz, 1H), 6.00 – 5.97 (m, 1H), 5.65 (ddd, *J* = 17.3, 10.4, 7.0 Hz, 1H), 5.01 (t, *J* = 1.4 Hz, 1H), 4.94 – 4.90 (m, 1H), 4.88 (d, *J* = 6.1 Hz, 1H), 4.27 (d, *J* = 14.1 Hz, 1H), 3.98 (tt, *J* = 10.8, 8.3, 3.6 Hz, 1H), 3.87 (dd, *J* = 14.2, 6.1 Hz, 1H), 3.80 (s, 3H), 3.78 (s, 3H), 3.75 (s, 4H), 3.61 (s, 3H), 3.30 (m, 1H), 3.16 (dd, *J* = 13.3, 10.5 Hz, 2H), 2.96 (ddd, *J* = 13.4, 5.3, 2.5 Hz, 2H), 2.88 (td, *J* = 11.8, 5.1 Hz, 1H), 2.53 – 2.47 (m, 2H), 2.01 (dddd, *J* = 16.1, 10.8, 5.5, 2.9 Hz, 3H), 1.74 – 1.66 (m, 2H), 1.36 (tt, *J* = 13.5, 3.2 Hz, 1H) ppm. ^{13}C NMR (126 MHz, CD_3OD) δ 178.0, 163.6, 161.0, 158.5, 158.3, 158.1, 147.0, 146.6, 143.2, 139.0, 138.8, 130.0, 128.8, 128.4, 128.2, 127.1, 126.2, 125.7, 122.2, 118.9, 115.0, 111.7, 108.6, 102.0, 100.9, 93.8, 91.5, 88.5, 67.6, 59.5, 57.2, 55.3, 54.7, 54.7, 54.4, 54.1, 53.0, 43.3, 37.8, 27.2, 24.7, 18.5 ppm. M.p. : 165-170 ºC (MeOH:CH_2Cl_2). α_D^{19} = -82.247 (*c* = 0.1, MeOH).

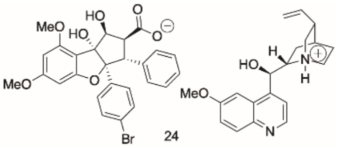

24

(1*S*,2*S*,4*S*,5*R*)-2-((*R*)-hydroxy(6-methoxyquinolin-4-yl)methyl)-5-vinylquinuclidin-1-ium (1*S*,2*S*,3*R*,3a*S*,8b*R*)-3a-(4-bromophenyl)-1,8b-dihydroxy-6,8-dimethoxy-3-phenyl-2,3,3a,8b-tetrahydro-1*H*-cyclopenta[*b*]benzofuran-2-carboxylate (24)

^{1}H NMR (500 MHz, CD_3OD) δ 8.64 (d, *J* = 4.6 Hz, 1H), 7.91 (d, *J* = 9.2 Hz, 1H), 7.71 (d, *J* = 4.6 Hz, 1H), 7.38 (dd, *J* = 9.2, 2.4 Hz, 1H), 7.33 (d, *J* = 2.6 Hz, 1H), 7.18 – 7.06 (m, 6H), 7.01 – 6.92 (m, 3H), 6.25 (d, *J* = 2.0 Hz, 1H), 6.11 (d, *J* = 2.0 Hz, 1H), 6.04 (s, 1H), 5.68 (ddd, *J* = 17.3, 10.4, 6.9 Hz, 1H), 5.05 – 4.94 (m, 2H), 4.82 (d, *J* = 5.8 Hz, 1H), 4.32 (d, *J* = 14.2 Hz, 1H), 4.08 – 3.98 (m, 1H), 3.86 (dd, *J* = 14.2, 5.8 Hz, 1H), 3.82 – 3.78 (m, 6H), 3.75 (s, 3H), 3.43 (t, *J* = 8.9 Hz, 1H), 3.28 – 3.24 (m, 1H), 3.05 (ddd, *J* = 13.3, 5.4, 2.4 Hz, 1H), 2.96 (td, *J* = 12.1, 5.0 Hz, 1H), 2.61 (t, *J* = 6.9 Hz, 1H), 2.12 – 2.07 (m, 1H), 1.97 (q, *J* = 3.2 Hz, 1H), 1.82 – 1.72 (m, 1H), 1.40 (ddt, *J* = 13.6, 10.4, 3.2 Hz, 1H) ppm. ^{13}C NMR (126 MHz, CD_3OD) δ 177.4, 163.6, 160.8, 158.6, 157.9, 146.6, 146.4, 143.2, 138.5, 138.4, 136.0, 130.0, 129.6, 129.2, 128.0, 127.2, 126.1, 125.8, 122.2, 120.2, 118.9, 115.2, 108.0, 101.7, 101.0, 93.9, 91.6, 88.4, 79.4, 67.1, 59.6, 57.0, 55.4, 54.7, 54.5, 54.1, 53.0, 43.4, 37.4, 27.0, 24.2, 18.2 ppm. M.p. : 175-180 ºC (MeOH:CH_2Cl_2). α_D^{20} = -18.00 (*c* = 0.1, MeOH).

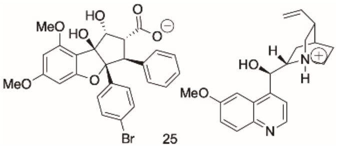

25

(1*S*,2*S*,4*S*,5*R*)-2-((*R*)-hydroxy(6-methoxyquinolin-4-yl)methyl)-5-vinylquinuclidin-1-ium (1*R*,2*R*,3*S*,3a*R*,8b*S*)-3a-(4-bromophenyl)-1,8b-dihydroxy-6,8-dimethoxy-3-phenyl-2,3,3a,8b-tetrahydro-1*H*-cyclopenta[*b*]benzofuran-2-carboxylate (25)

^{1}H NMR (500 MHz, CD_3OD) δ 8.65 (d, *J* = 4.6 Hz, 1H), 7.93 (d, *J* = 9.1 Hz, 2H), 7.72 (d, *J* = 4.6 Hz, 1H), 7.40 (d, *J* = 9.2 Hz, 3H), 7.18 – 7.06 (m, 5H), 7.03 – 6.92 (m, 3H), 6.27 (d, *J* = 2.0 Hz, 1H), 6.16 (d, *J* = 2.0 Hz, 1H), 5.97 (s, 1H), 5.71 (ddd, *J* = 17.3, 10.4, 7.0 Hz, 1H), 5.04 (d, *J* = 17.1 Hz, 1H), 4.97 (d, *J* = 10.4 Hz, 1H), 4.80 (d, *J* = 5.8 Hz, 1H), 4.33 (d, *J* = 14.2 Hz, 1H), 4.02 (s, 1H), 3.87 – 3.83 (m, 1H), 3.82 – 3.80 (m, 9H), 3.47 – 3.41 (m, 2H), 3.25 (d, *J* = 11.9 Hz, 1H), 3.06 (d, *J* = 12.7 Hz, 1H), 2.97 (s, 1H), 2.60 (s, 1H), 2.08 (d, *J* = 7.7 Hz, 1H), 1.98 (d, *J* = 3.2 Hz, 1H), 1.43 (tt, *J* = 10.4, 3.1 Hz, 1H) ppm. ^{13}C NMR (126 MHz, CD_3OD) δ 179.3, 164.3,

160.8, 158.6, 157.7, 146.6, 143.3, 139.1, 138.0, 136.0, 130.0, 129.6, 129.2, 128.0, 127.2, 126.2, 125.8, 122.1, 120.2, 118.9, 115.1, 108.1, 101.8, 101.0, 93.9, 91.6, 88.2, 79.3, 67.5, 59.6, 57.1, 55.3, 54.7, 54.5, 54.3, 43.4, 37.6, 27.1, 24.4, 18.4 ppm. M.p. : 170-175 ºC (MeOH:CH_2Cl_2). α_D^{21} = -77.52 (*c* = 0.1, MeOH).

Additional optical rotations for compounds in this study:

(-)-10, α_D^{21} = -46.32 (*c* = 0.1, $CHCl_3$)
(+)-10, α_D^{24} = +45.14 (*c* = 0.1, $CHCl_3$)
(-)-11, α_D^{20} = -25.924 (*c* = 0.1, $CHCl_3$)
(+)-11, α_D^{21} = +28.523 (*c* = 0.1, $CHCl_3$)
(-)-13, α_D^{21} = -141.32 (*c* = 0.1, $CHCl_3$)
(-)-15, α_D^{21} = -40.22 (*c* = 0.1, $CHCl_3$)
(+)-15, α_D^{23} = +39.48 (*c* = 0.1, $CHCl_3$)
(-)-16, α_D^{22} = -27.60 (*c* = 0.1, $CHCl_3$)
(+)-16, α_D^{23} = +28.44 (*c* = 0.1, $CHCl_3$)
(-)-19, α_D^{21} = -20.88 (*c* = 0.1, $CHCl_3$)
(-)-20, α_D^{22} = -20.49 (*c* = 0.1, $CHCl_3$)

VI. Select NMR Spectra

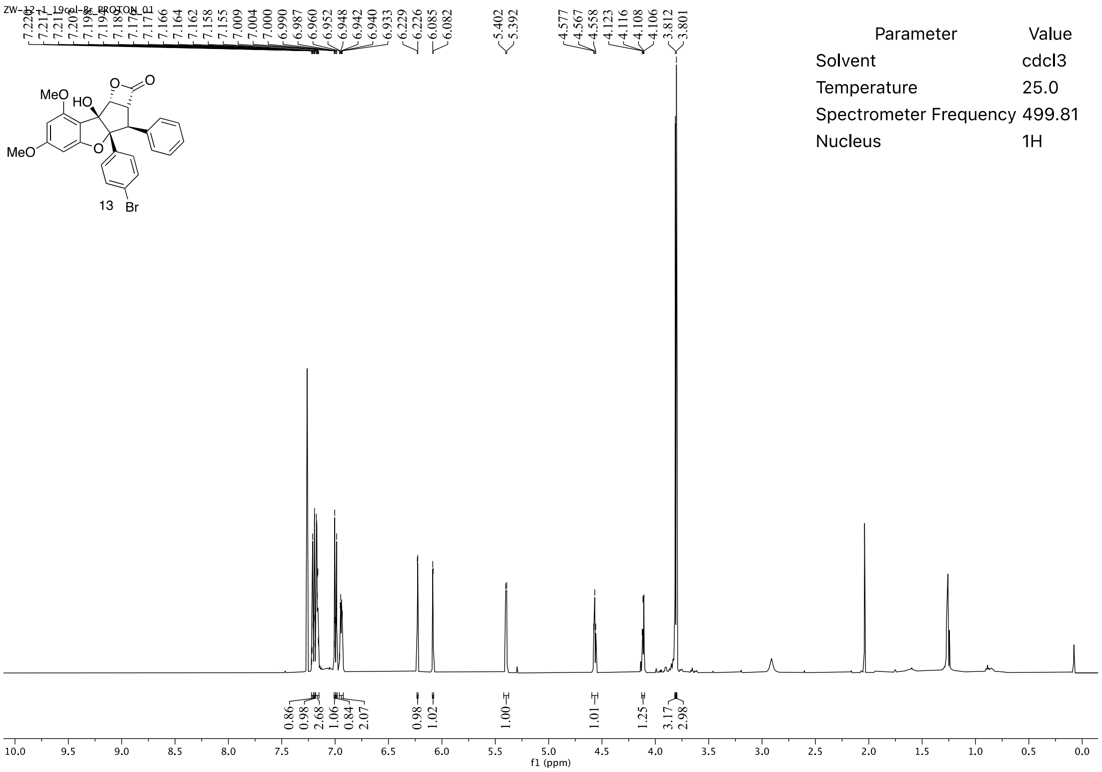

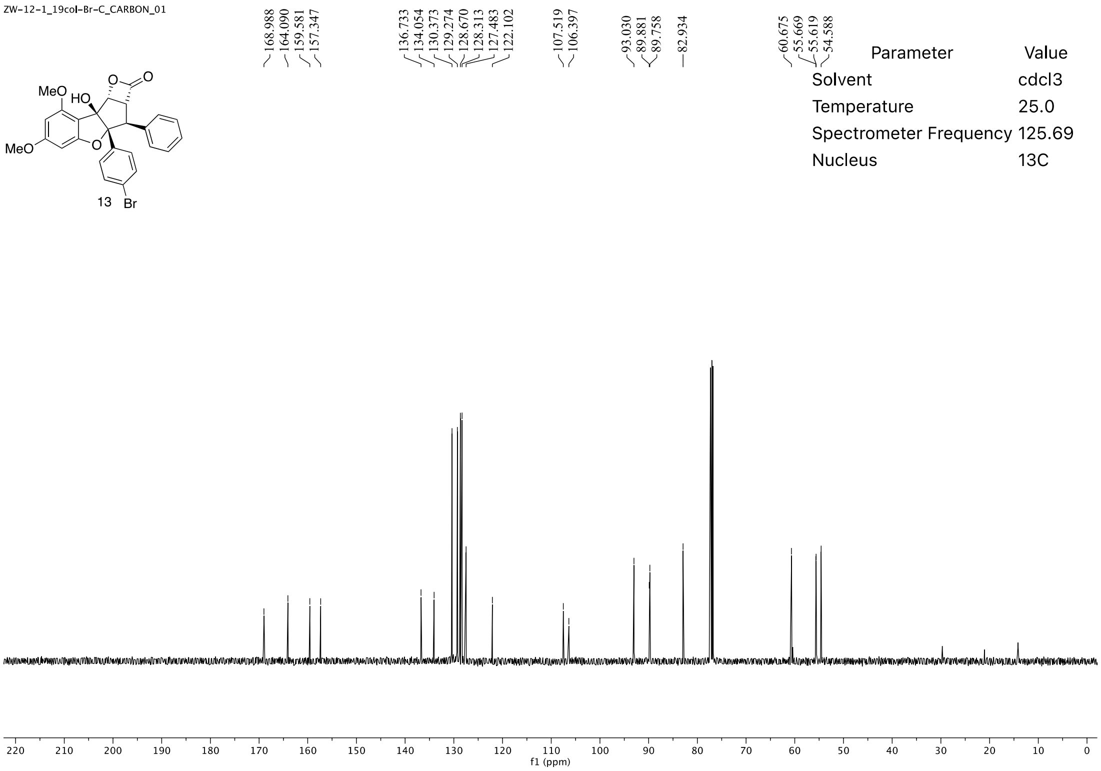

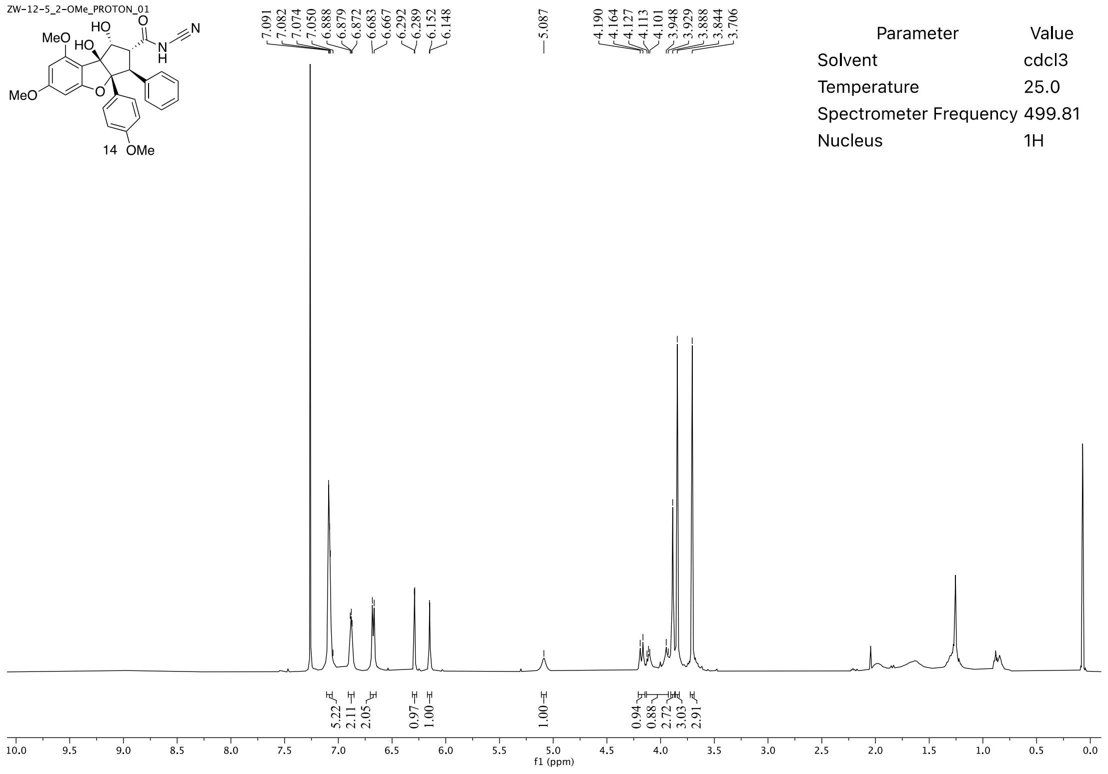
ZW-12-5_2-OMe_PROTON_01
MeO
HO
HO
O
N
H
N
MeO
O
14 OMe
7.091
7.082
7.074
7.050
6.888
6.879
6.872
6.683
6.667
6.292
6.289
6.152
6.148
5.087
4.190
4.164
4.127
4.113
4.101
3.948
3.929
3.888
3.844
3.706
Parameter Value
Solvent cdcl3
Temperature 25.0
Spectrometer Frequency 499.81
Nucleus 1H
5.22
2.11
2.05
0.97
1.00
1.00
0.94
0.88
2.72
3.03
2.91
f1 (ppm)

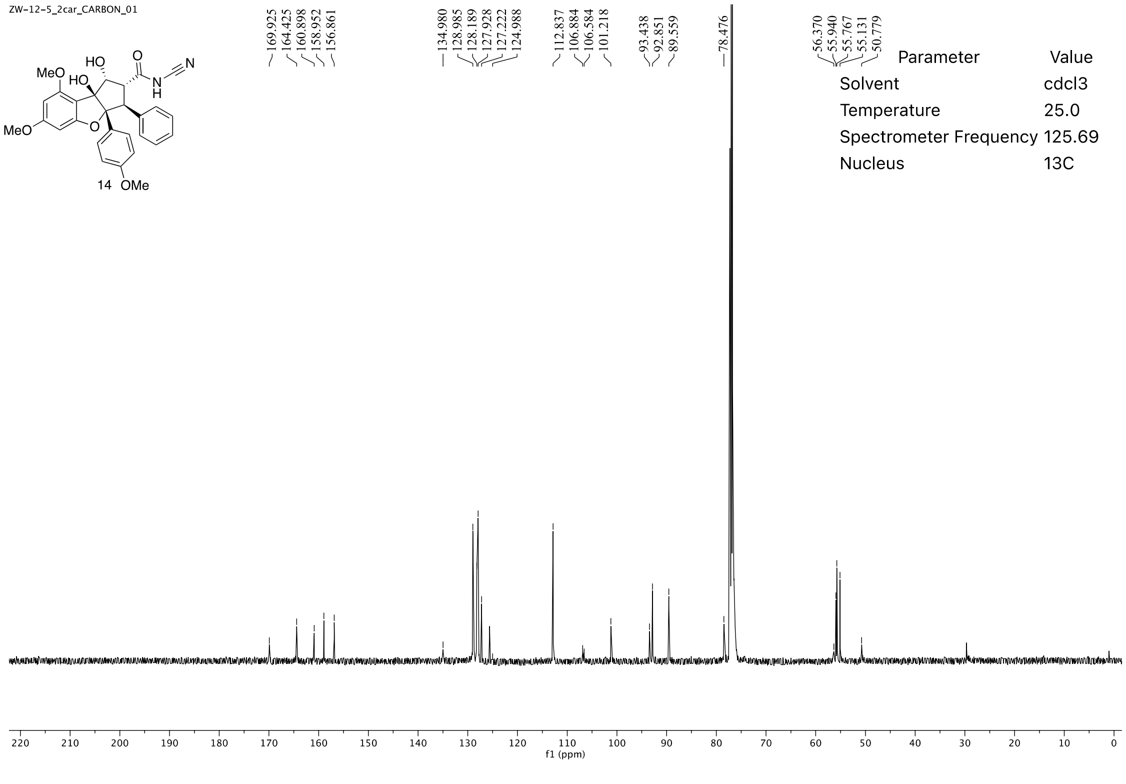
ZW-12-5_2car_CARBON_01
MeO
HO
HO
O
N
H
N
MeO
O
14 OMe
169.925
164.425
160.898
158.952
156.861
134.980
128.985
128.189
127.928
127.222
124.988
112.837
106.884
106.584
101.218
93.438
92.851
89.559
78.476
56.370
55.940
55.767
55.131
50.779
Parameter Value
Solvent cdcl3
Temperature 25.0
Spectrometer Frequency 125.69
Nucleus 13C
f1 (ppm)

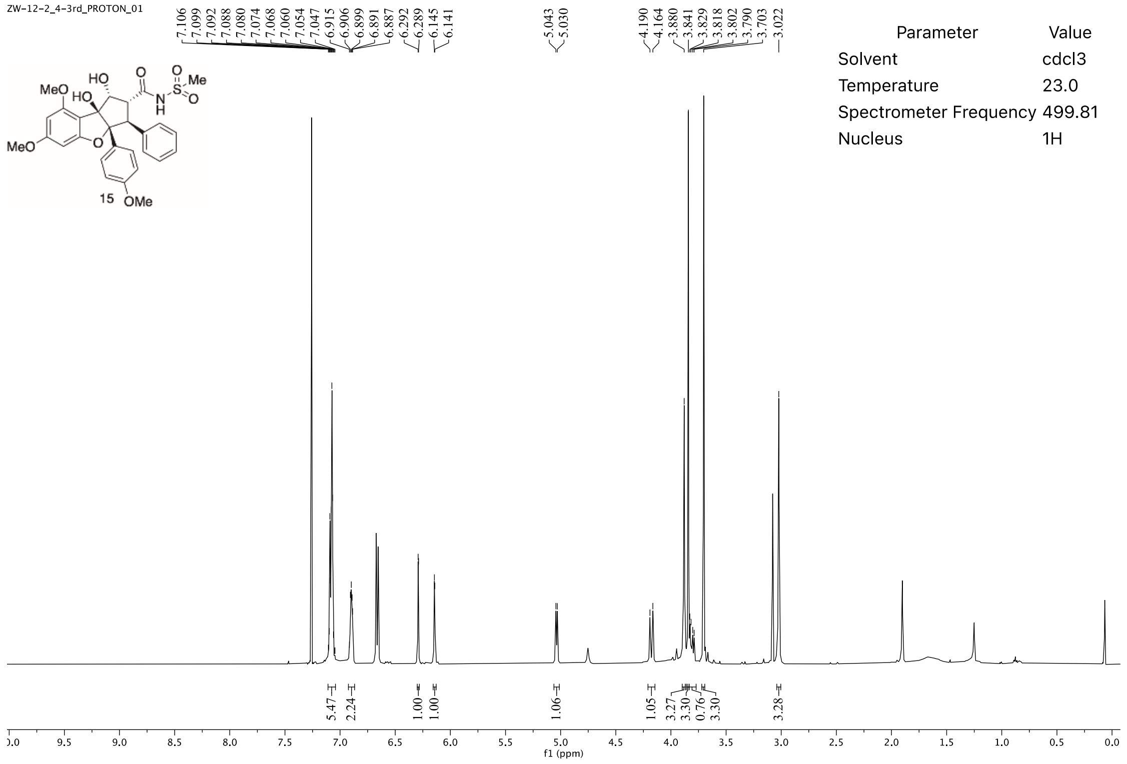
ZW-12-2_4-3rd_PROTON_01
7.106 7.099 7.092 7.088 7.080 7.074 7.068 7.060 7.054 7.047 6.915 6.906 6.899 6.891 6.887 6.292 6.289 6.145 6.141
5.043 5.030
4.190 4.164 3.880 3.841 3.829 3.818 3.802 3.790 3.703 3.022
Parameter Value
Solvent cdcl3
Temperature 23.0
Spectrometer Frequency 499.81
Nucleus 1H
15
5.47 2.24 1.00 1.00 1.06 1.05 3.27 3.30 0.76 3.30 3.28
f1 (ppm)

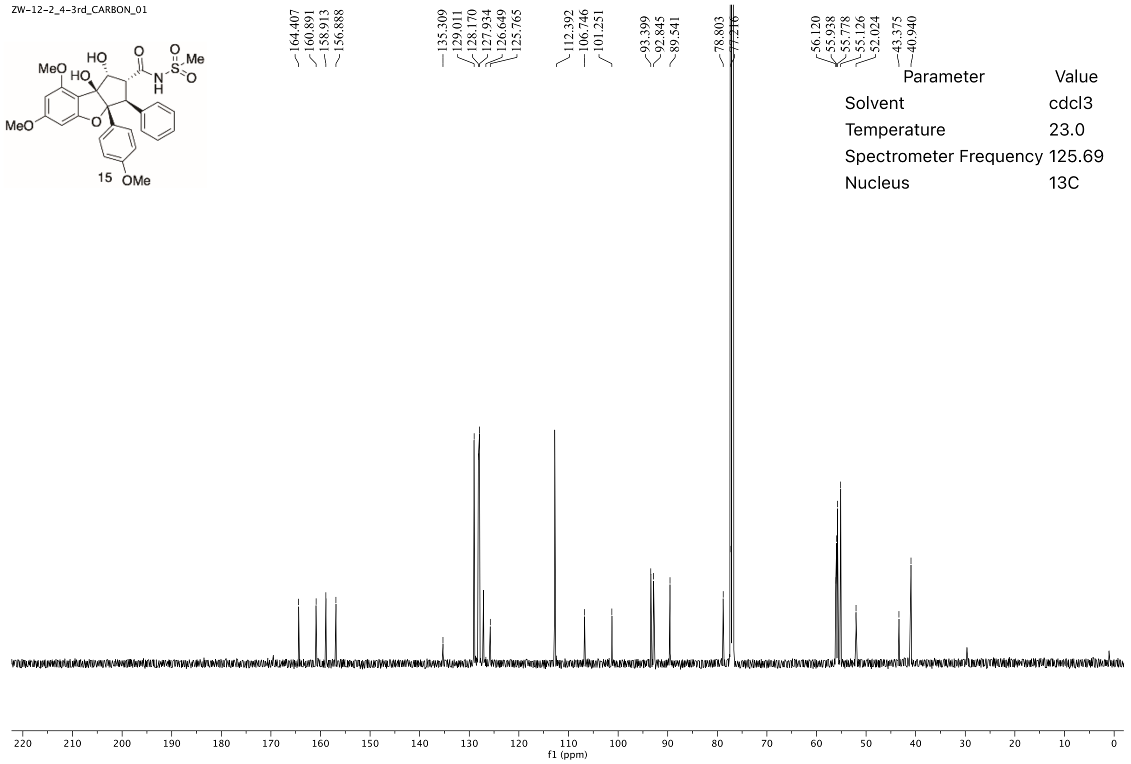
ZW-12-2_4-3rd_CARBON_01
164.407 160.891 158.913 156.888
135.309 129.011 128.170 127.934 126.649 125.765
112.392 106.746 101.251
93.399 92.845 89.541
78.803 77.216
56.120 55.938 55.778 55.126 52.024 43.375 40.940
Parameter Value
Solvent cdcl3
Temperature 23.0
Spectrometer Frequency 125.69
Nucleus 13C
15
f1 (ppm)

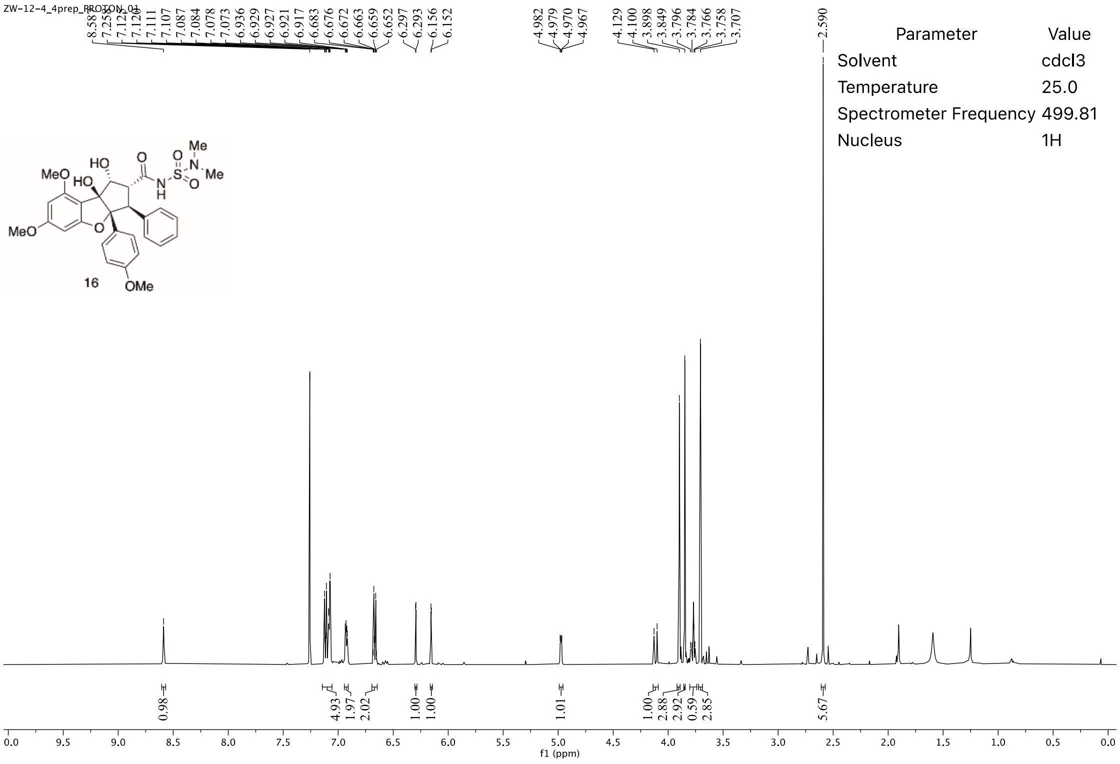

Parameter	Value
Solvent	cdcl3
Temperature	25.0
Spectrometer Frequency	499.81
Nucleus	1H

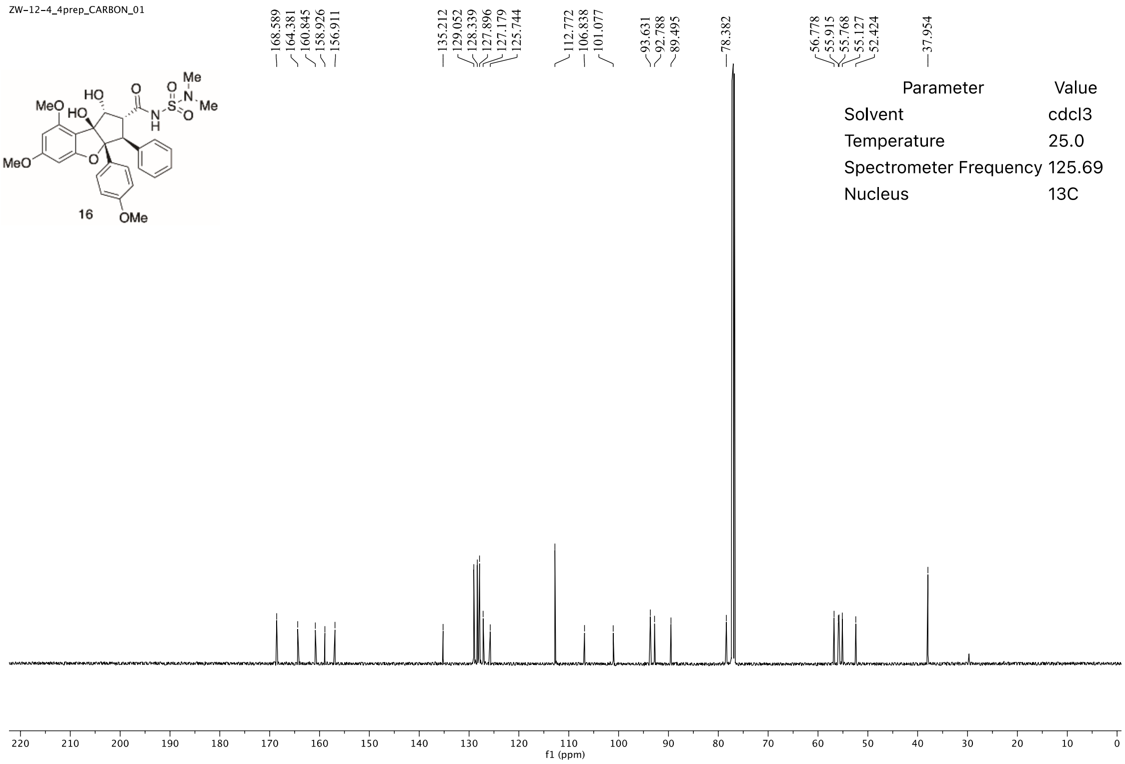

Parameter	Value
Solvent	cdcl3
Temperature	25.0
Spectrometer Frequency	125.69
Nucleus	13C

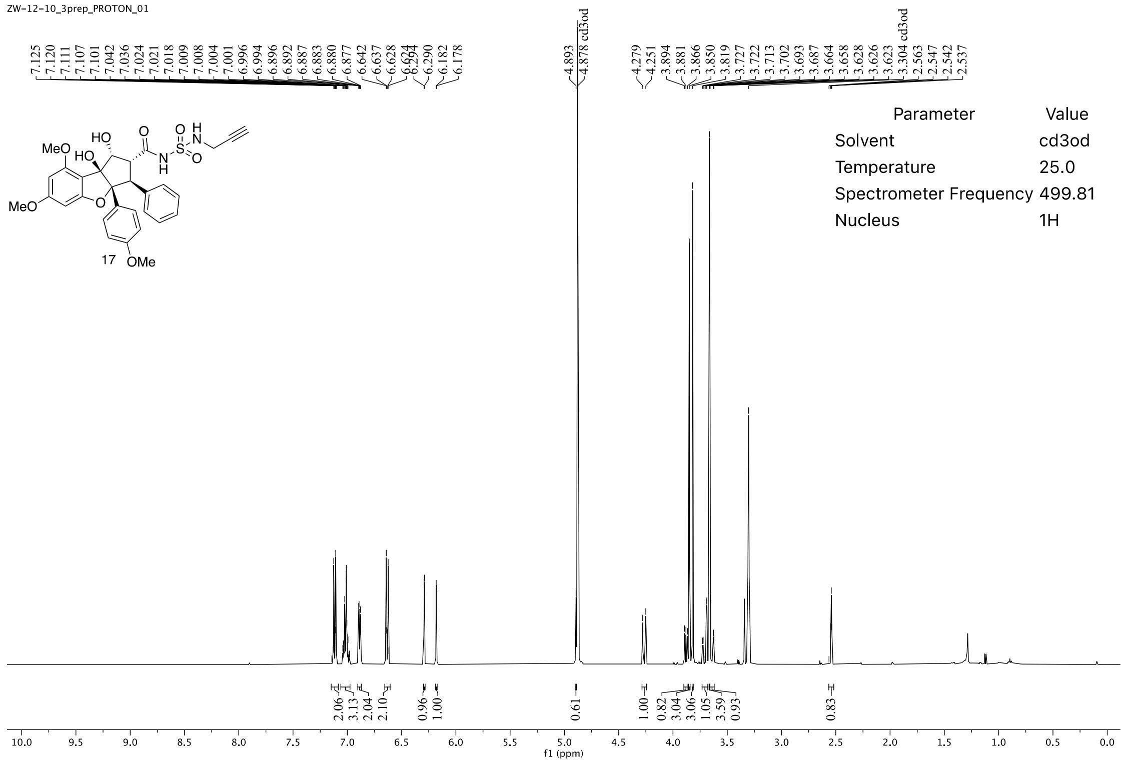
ZW-12-10_3prep_PROTON_01
7.125 7.120 7.111 7.107 7.101 7.042 7.036 7.024 7.021 7.018 7.009 7.008 7.004 7.001 6.996 6.994 6.896 6.892 6.887 6.883 6.880 6.877 6.642 6.637 6.628 6.624 6.294 6.290 6.182 6.178
4.893 4.878 cd3od
4.279 4.251 3.894 3.881 3.866 3.850 3.819 3.727 3.722 3.713 3.702 3.693 3.687 3.664 3.658 3.628 3.626 3.623 3.304 cd3od 2.563 2.547 2.542 2.537
Parameter Value
Solvent cd3od
Temperature 25.0
Spectrometer Frequency 499.81
Nucleus 1H
MeO
HO
HO
O
O
S
O
H
N
N
H
MeO
O
17
OMe
2.06 3.13 2.04 2.10 0.96 1.00 0.61 1.00 0.82 3.04 3.06 1.05 3.59 0.93 0.83
f1 (ppm)

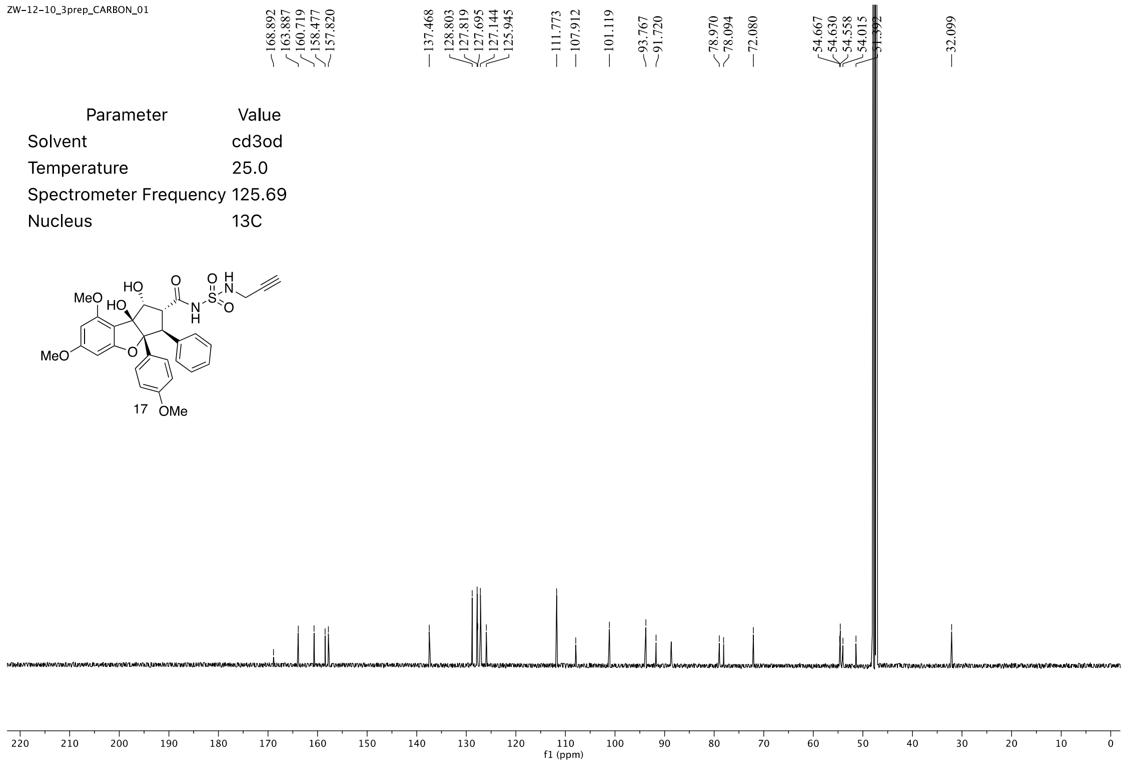
ZW-12-10_3prep_CARBON_01
168.892 163.887 160.719 158.477 157.820 137.468 128.803 127.819 127.695 127.144 125.945 111.773 107.912 101.119 93.767 91.720 78.970 78.094 72.080 54.667 54.630 54.558 54.015 51.392 32.099
Parameter Value
Solvent cd3od
Temperature 25.0
Spectrometer Frequency 125.69
Nucleus 13C
MeO
HO
HO
O
O
S
O
H
N
N
H
MeO
O
17
OMe
f1 (ppm)

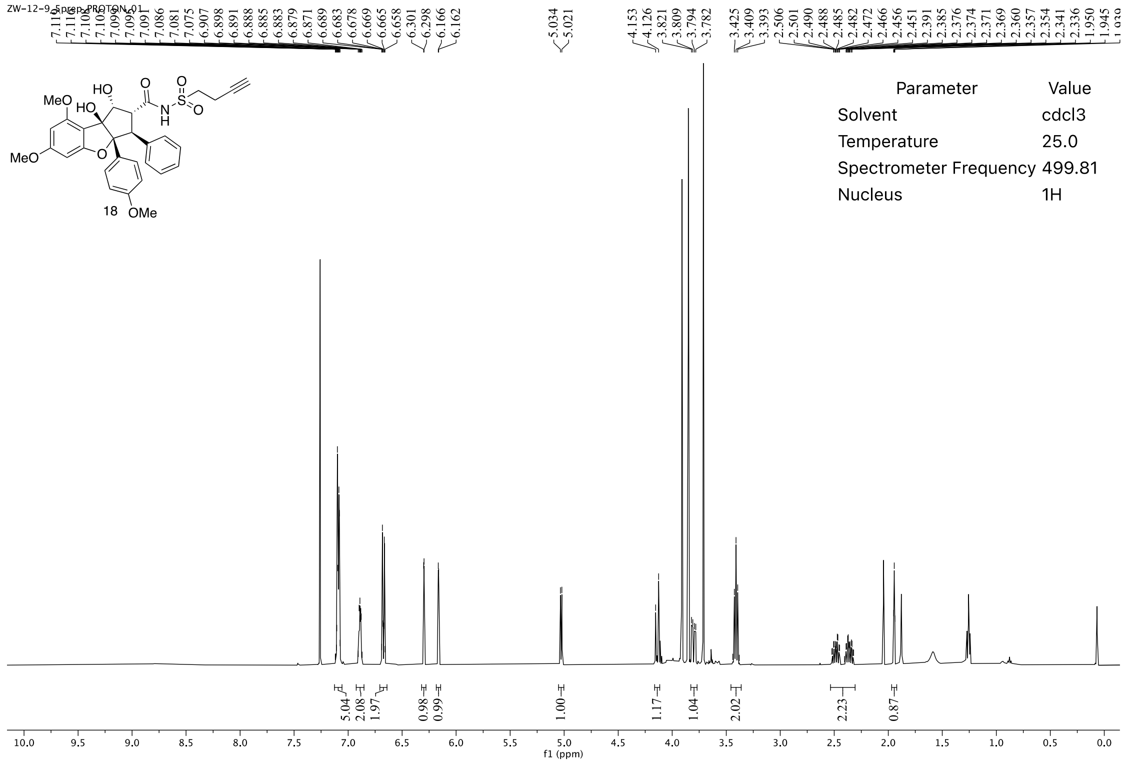
ZW-12-9_5prep_PROTON_01
7.091
7.086
7.081
7.075
6.907
6.898
6.891
6.888
6.885
6.883
6.879
6.871
6.689
6.683
6.678
6.669
6.665
6.658
6.301
6.298
6.166
6.162
5.034
5.021
4.153
4.126
3.821
3.809
3.794
3.782
3.425
3.409
3.393
2.506
2.501
2.490
2.488
2.485
2.482
2.472
2.466
2.456
2.451
2.391
2.385
2.376
2.374
2.371
2.369
2.360
2.357
2.354
2.341
2.336
1.950
1.945
MeO
HO
HO
O
S
O
O
N
H
MeO
O
18
OMe
Parameter
Value
Solvent
cdcl3
Temperature
25.0
Spectrometer Frequency
499.81
Nucleus
1H
5.04
2.08
1.97
0.98
0.99
1.00
1.17
1.04
2.02
2.23
0.87
10.0
9.5
9.0
8.5
8.0
7.5
7.0
6.5
6.0
5.5
5.0
4.5
4.0
3.5
3.0
2.5
2.0
1.5
1.0
0.5
0.0
f1 (ppm)

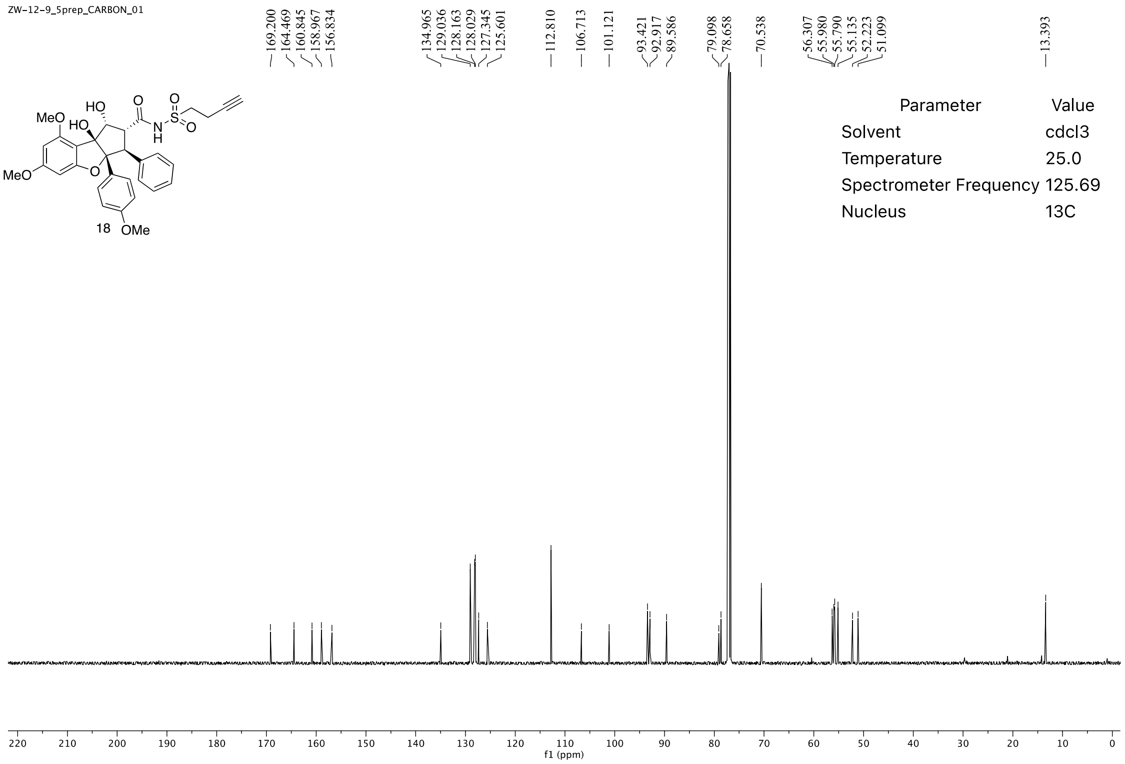
ZW-12-9_5prep_CARBON_01
169.200
164.469
160.845
158.967
156.834
134.965
129.036
128.163
128.029
127.345
125.601
112.810
106.713
101.121
93.421
92.917
89.586
79.098
78.658
70.538
56.307
55.980
55.790
55.135
52.223
51.099
13.393
MeO
HO
HO
O
S
O
O
N
H
MeO
O
18
OMe
Parameter
Value
Solvent
cdcl3
Temperature
25.0
Spectrometer Frequency
125.69
Nucleus
13C
220
210
200
190
180
170
160
150
140
130
120
110
100
90
80
70
60
50
40
30
20
10
0
f1 (ppm)

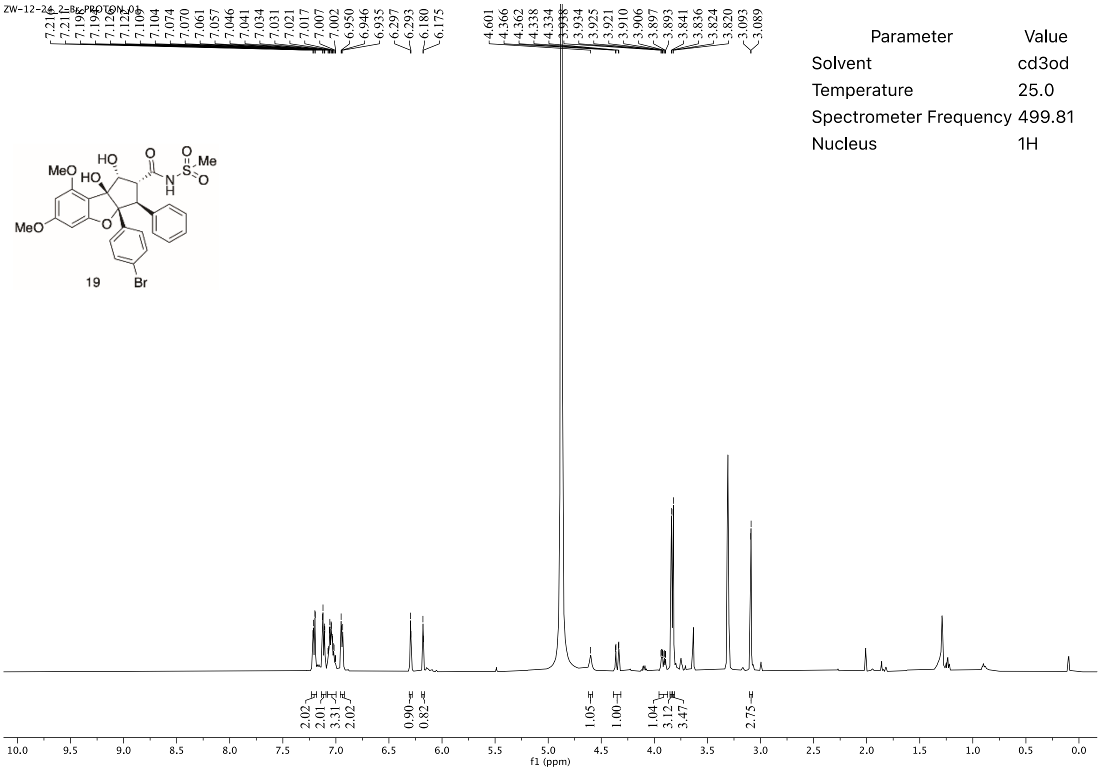
ZW-12-24_2-Br_PROTON_01
Parameter Value
Solvent cd3od
Temperature 25.0
Spectrometer Frequency 499.81
Nucleus 1H
19
f1 (ppm)

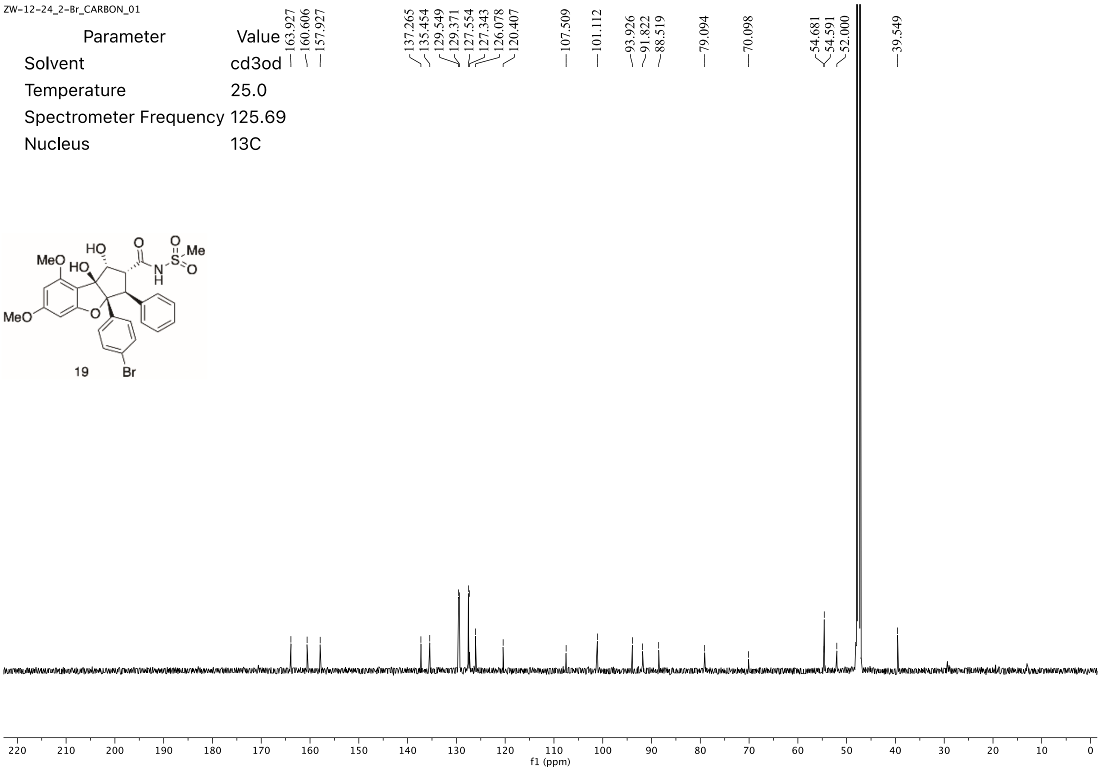
ZW-12-24_2-Br_CARBON_01
Parameter Value
Solvent cd3od
Temperature 25.0
Spectrometer Frequency 125.69
Nucleus 13C
19
f1 (ppm)

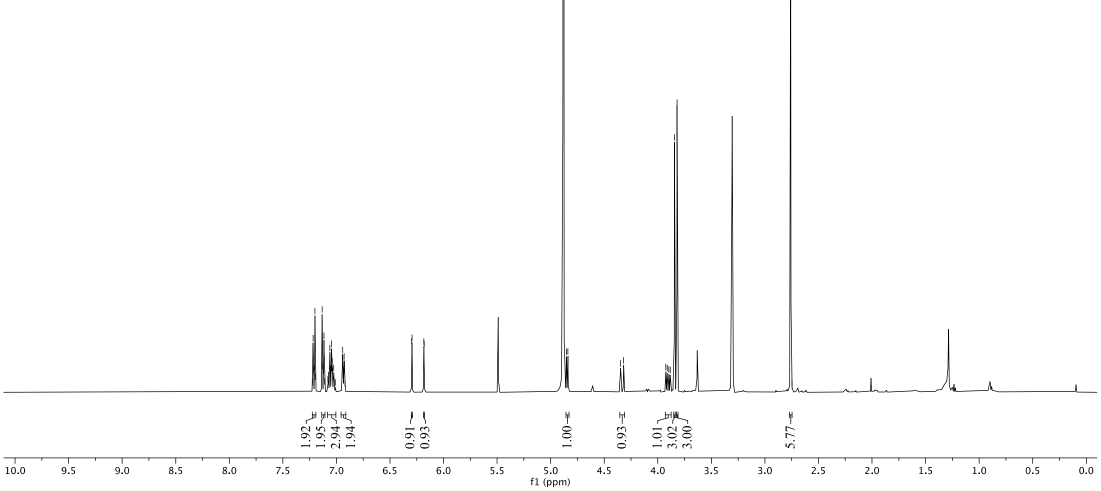

Parameter	Value
Solvent	cd3od
Temperature	25.0
Spectrometer Frequency	499.81
Nucleus	1H

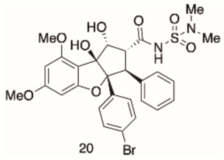

20

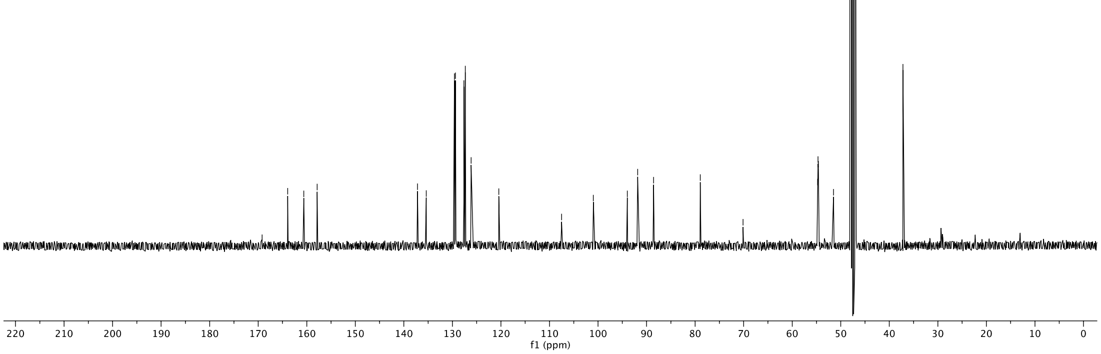

Parameter	Value
Solvent	cd3od
Temperature	25.0
Spectrometer Frequency	125.69
Nucleus	13C

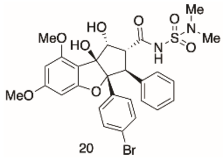

20

ZW-12-26prep2_PROTON_01

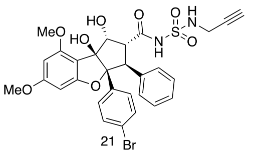

Parameter	Value
Solvent	cd3od
Temperature	25.0
Spectrometer Frequency	499.81
Nucleus	1H

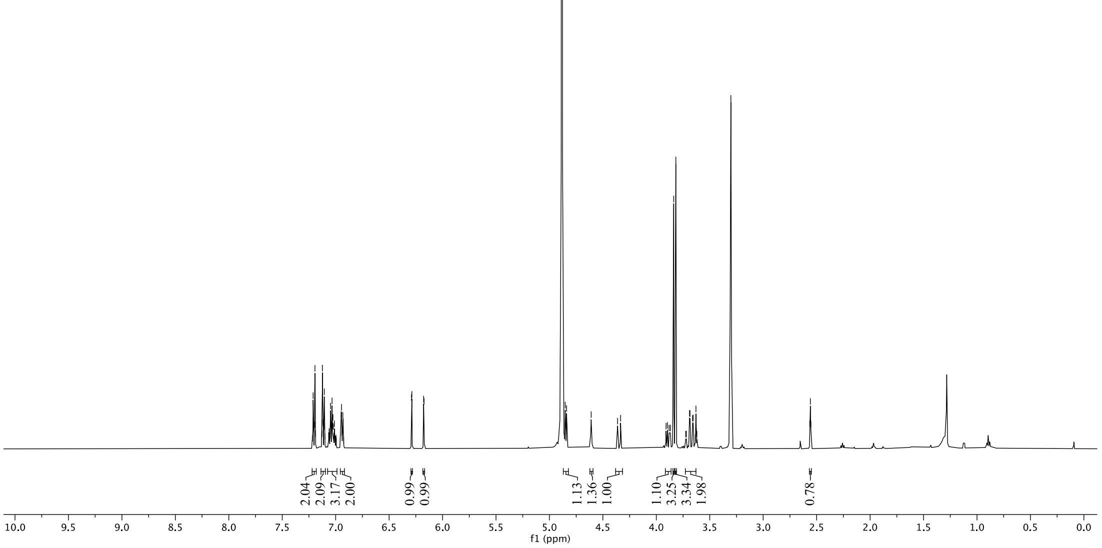

ZW-12-26_2_CARBON_01

Parameter	Value
Solvent	cd3od
Temperature	25.0
Spectrometer Frequency	125.69
Nucleus	13C

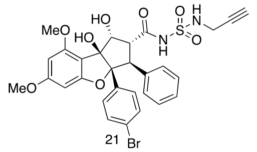

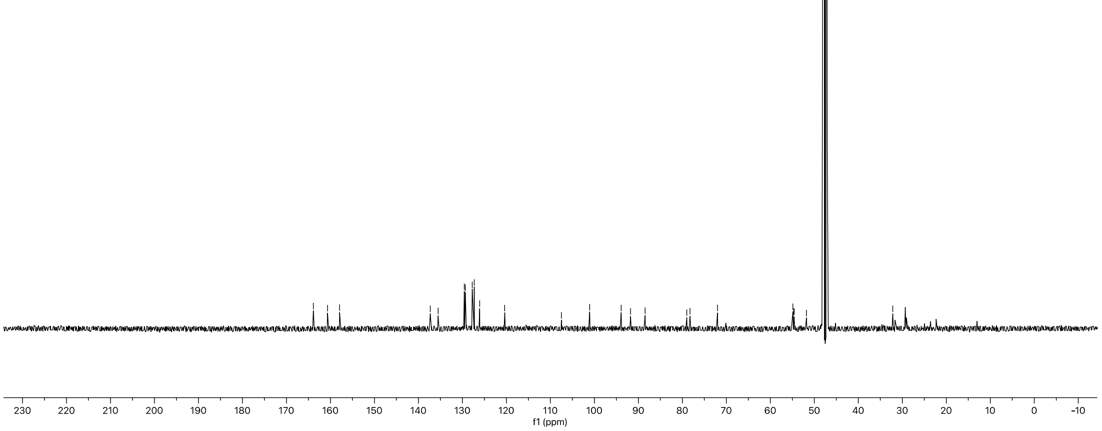

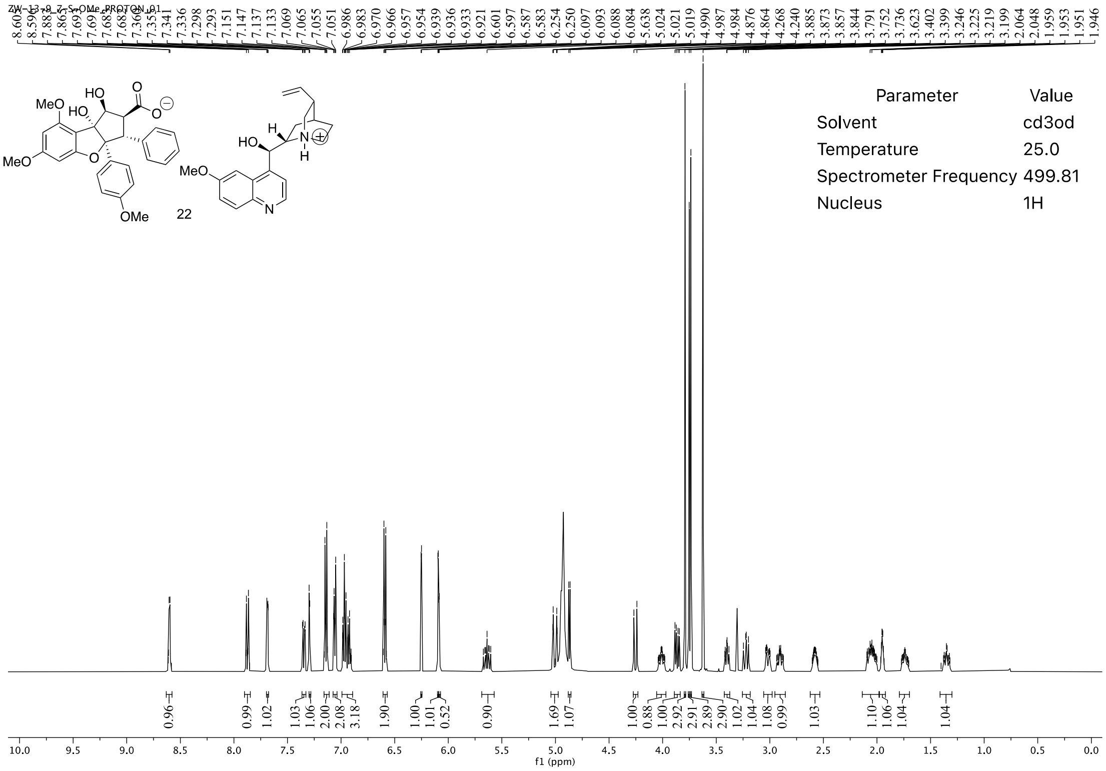
ZW-13-9_7-S-OMe_PROTON_01
7.336
7.298
7.293
7.151
7.147
7.137
7.133
7.069
7.065
7.055
7.051
6.986
6.983
6.970
6.966
6.957
6.954
6.939
6.936
6.933
6.921
6.601
6.597
6.587
6.583
6.254
6.250
6.097
6.093
6.088
6.084
5.638
5.024
5.021
5.019
4.990
4.987
4.984
4.876
4.864
4.268
4.240
3.885
3.873
3.857
3.844
3.791
3.752
3.736
3.623
3.402
3.399
3.246
3.225
3.219
3.199
2.064
2.048
1.959
1.953
1.951
1.946
Parameter Value
Solvent cd3od
Temperature 25.0
Spectrometer Frequency 499.81
Nucleus 1H
MeO
HO
HO
O
O
MeO
OMe
22
H
HO
N
H
MeO
N
0.96
0.99
1.02
1.03
1.06
2.00
2.08
3.18
1.90
1.00
1.01
0.52
0.90
1.69
1.07
1.00
0.88
1.00
2.92
2.91
2.89
2.90
1.02
1.04
1.08
0.99
1.03
1.10
1.06
1.04
1.04
10.0 9.5 9.0 8.5 8.0 7.5 7.0 6.5 6.0 5.5 5.0 4.5 4.0 3.5 3.0 2.5 2.0 1.5 1.0 0.5 0.0
f1 (ppm)

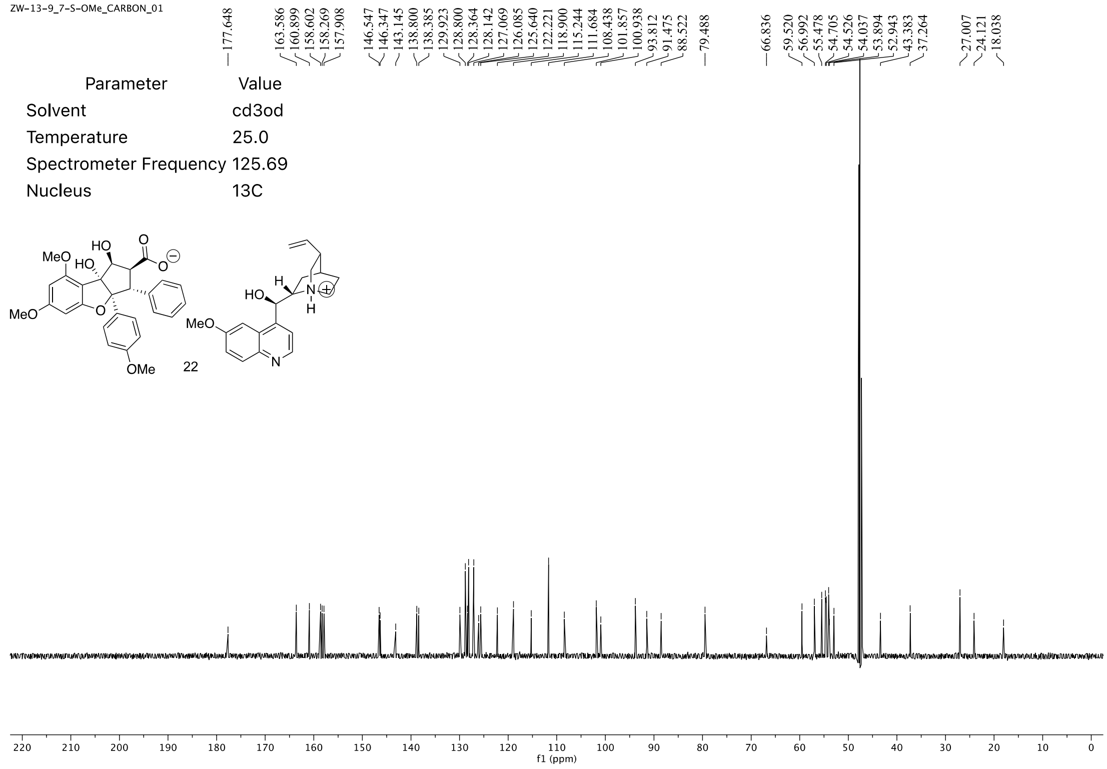
ZW-13-9_7-S-OMe_CARBON_01
177.648
163.586
160.899
158.602
158.269
157.908
146.547
146.347
143.145
138.800
138.385
129.923
128.800
128.364
128.142
127.069
126.085
125.640
122.221
118.900
115.244
111.684
108.438
101.857
100.938
93.812
91.475
88.522
79.488
66.836
59.520
56.992
55.478
54.705
54.526
54.037
53.894
52.943
43.383
37.264
27.007
24.121
18.038
Parameter Value
Solvent cd3od
Temperature 25.0
Spectrometer Frequency 125.69
Nucleus 13C
MeO
HO
HO
O
O
MeO
OMe
22
H
HO
N
H
MeO
N
220 210 200 190 180 170 160 150 140 130 120 110 100 90 80 70 60 50 40 30 20 10 0
f1 (ppm)

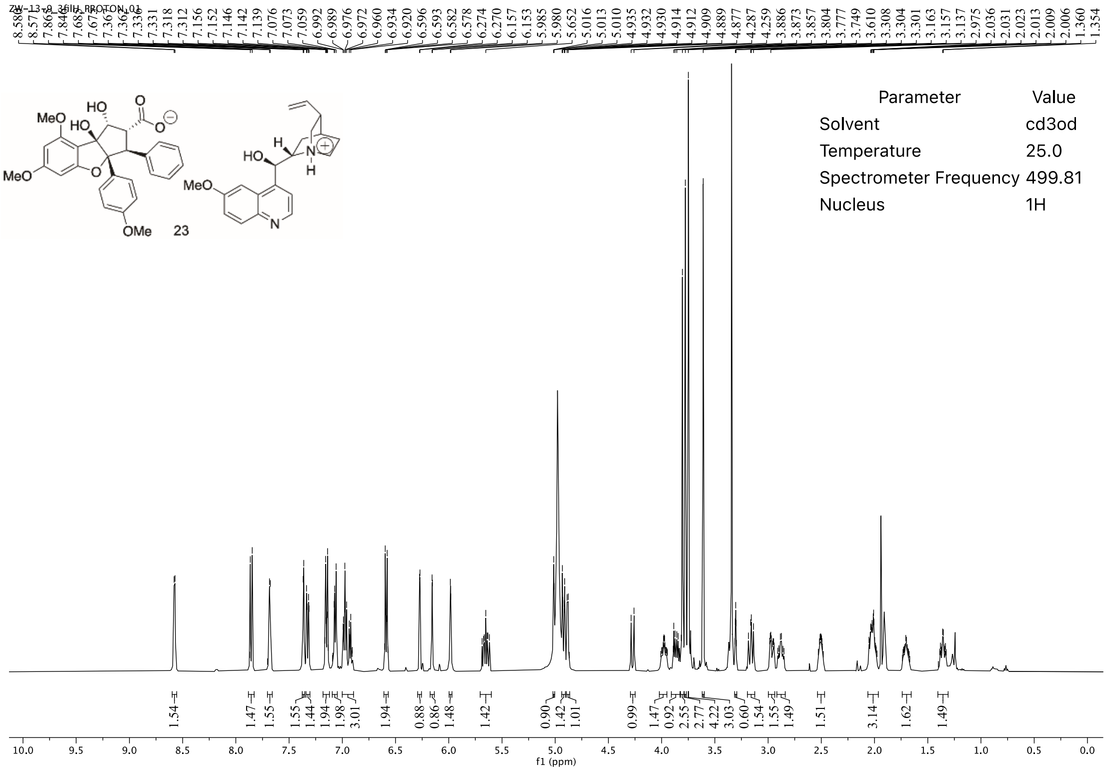

Parameter	Value
Solvent	cd3od
Temperature	25.0
Spectrometer Frequency	499.81
Nucleus	1H

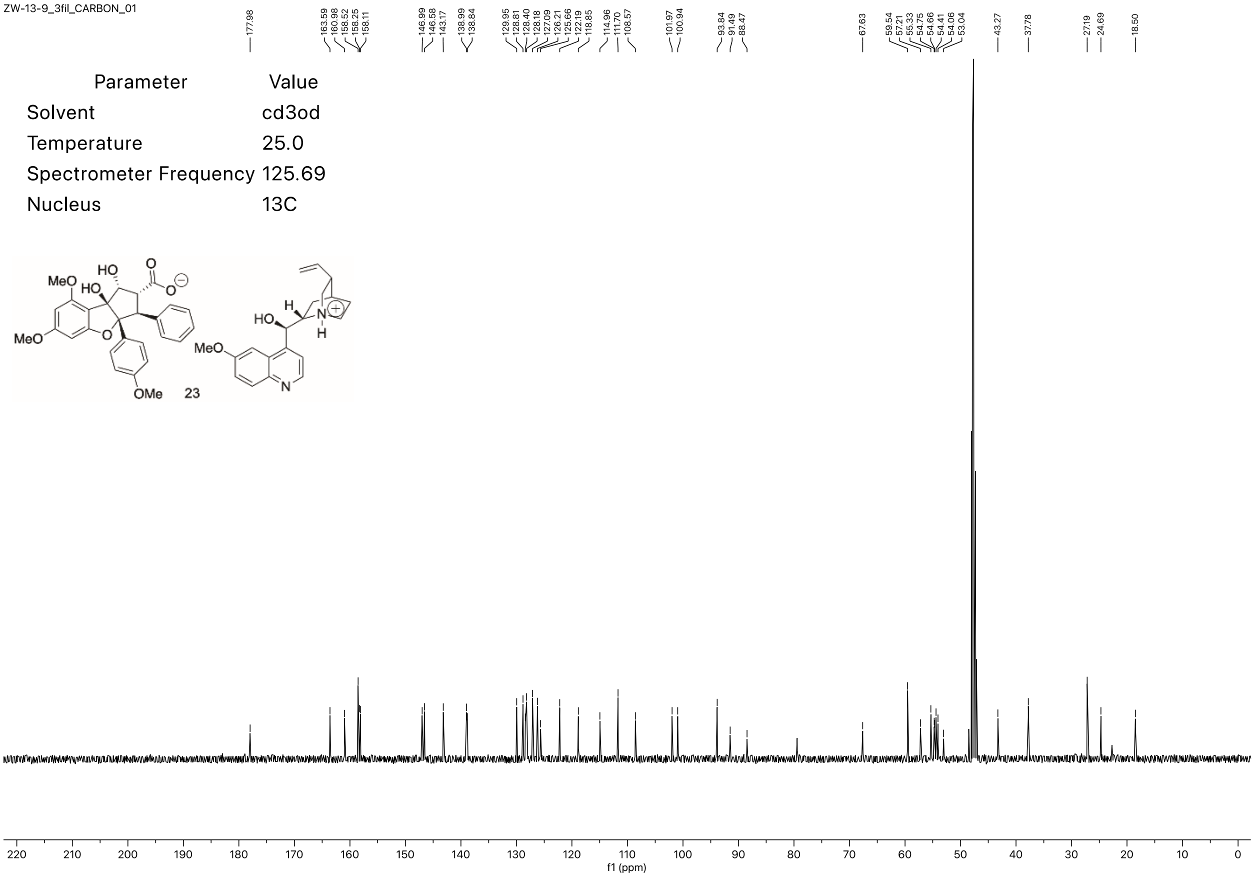

Parameter	Value
Solvent	cd3od
Temperature	25.0
Spectrometer Frequency	125.69
Nucleus	13C

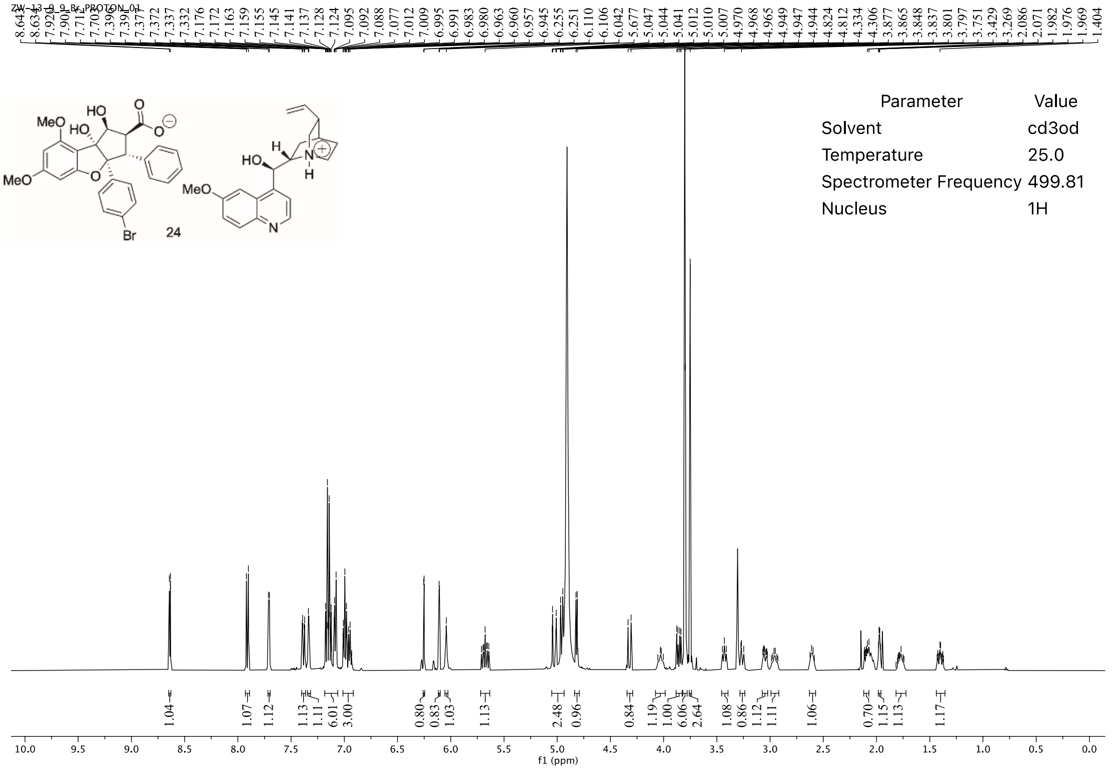

Parameter	Value
Solvent	cd3od
Temperature	25.0
Spectrometer Frequency	499.81
Nucleus	1H

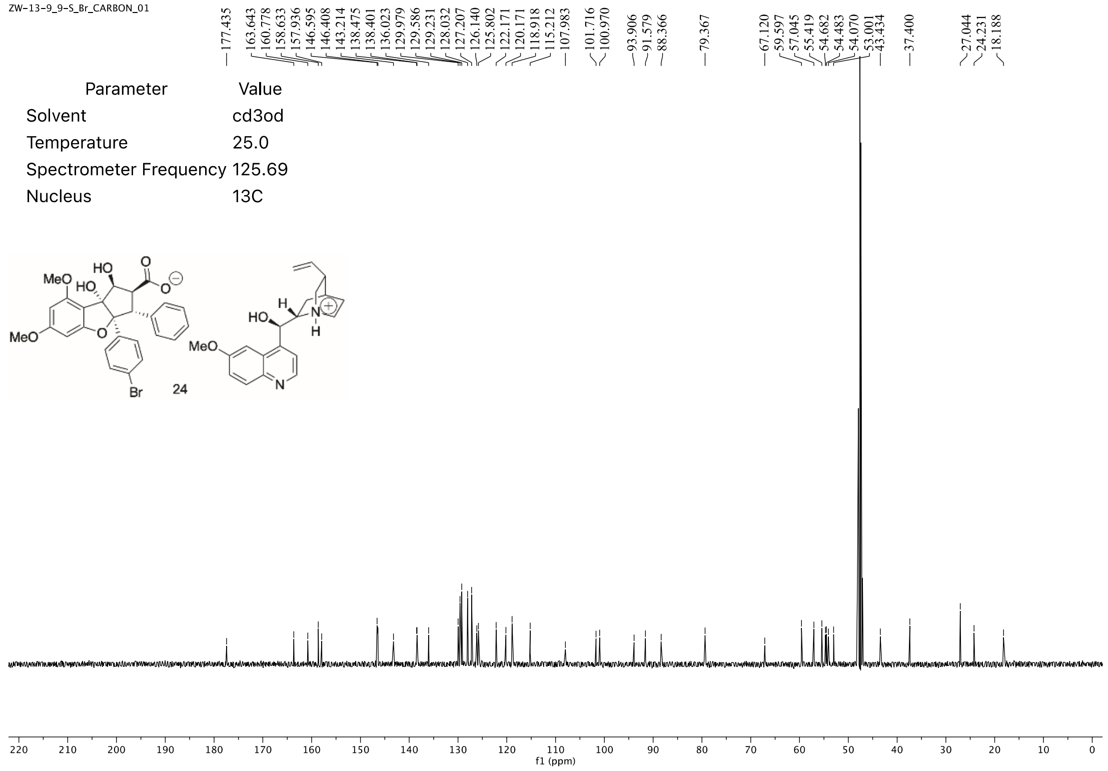

Parameter	Value
Solvent	cd3od
Temperature	25.0
Spectrometer Frequency	125.69
Nucleus	13C

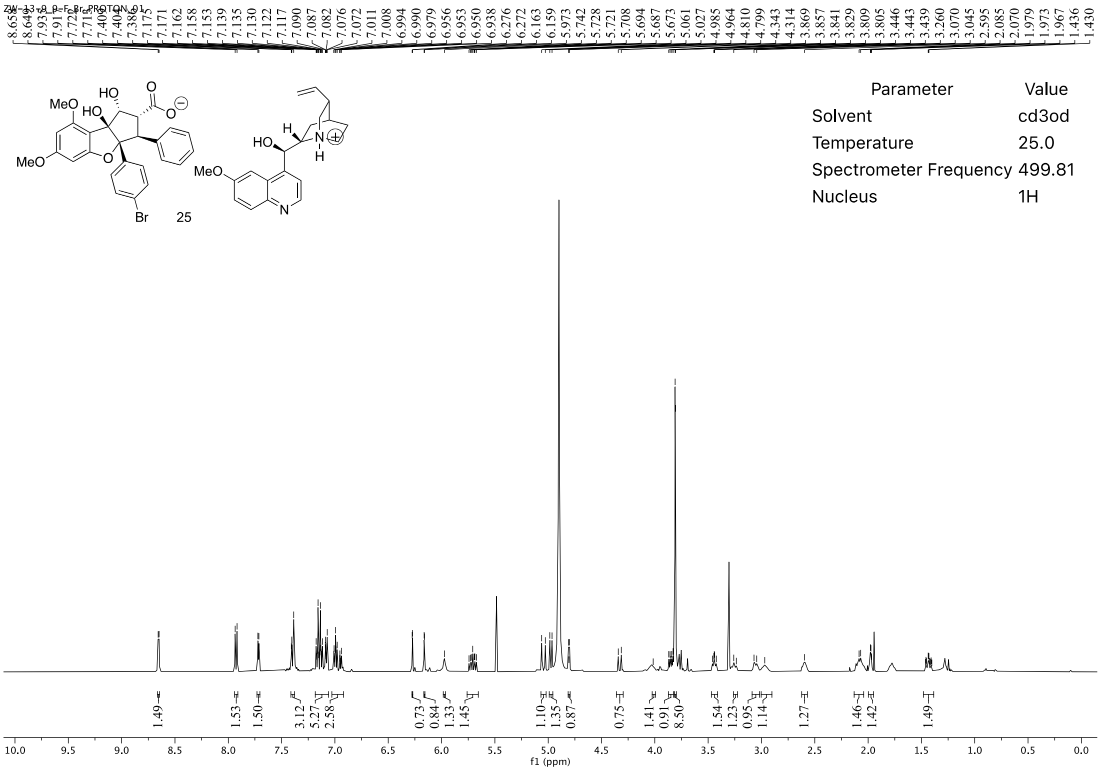
ZW-13-9_9-F_Br_PROTON_01
8.658
8.649
7.933
7.404
7.386
7.175
7.171
7.162
7.158
7.153
7.139
7.135
7.130
7.122
7.117
7.090
7.087
7.082
7.076
7.072
7.011
7.008
6.994
6.990
6.979
6.956
6.953
6.950
6.938
6.276
6.272
6.163
6.159
5.973
5.742
5.728
5.721
5.708
5.694
5.687
5.673
5.061
5.027
4.985
4.964
4.810
4.799
4.343
4.314
3.869
3.857
3.841
3.829
3.809
3.805
3.446
3.443
3.439
3.260
3.070
3.045
2.595
2.085
2.070
1.979
1.973
1.967
1.436
1.430
Parameter | Value
Solvent | cd3od
Temperature | 25.0
Spectrometer Frequency | 499.81
Nucleus | 1H
25
1.49
1.53
1.50
3.12
5.27
2.58
0.73
0.84
1.33
1.45
1.10
1.35
0.87
0.75
1.41
0.91
8.50
1.54
1.23
0.95
1.14
1.27
1.46
1.42
1.49
f1 (ppm)

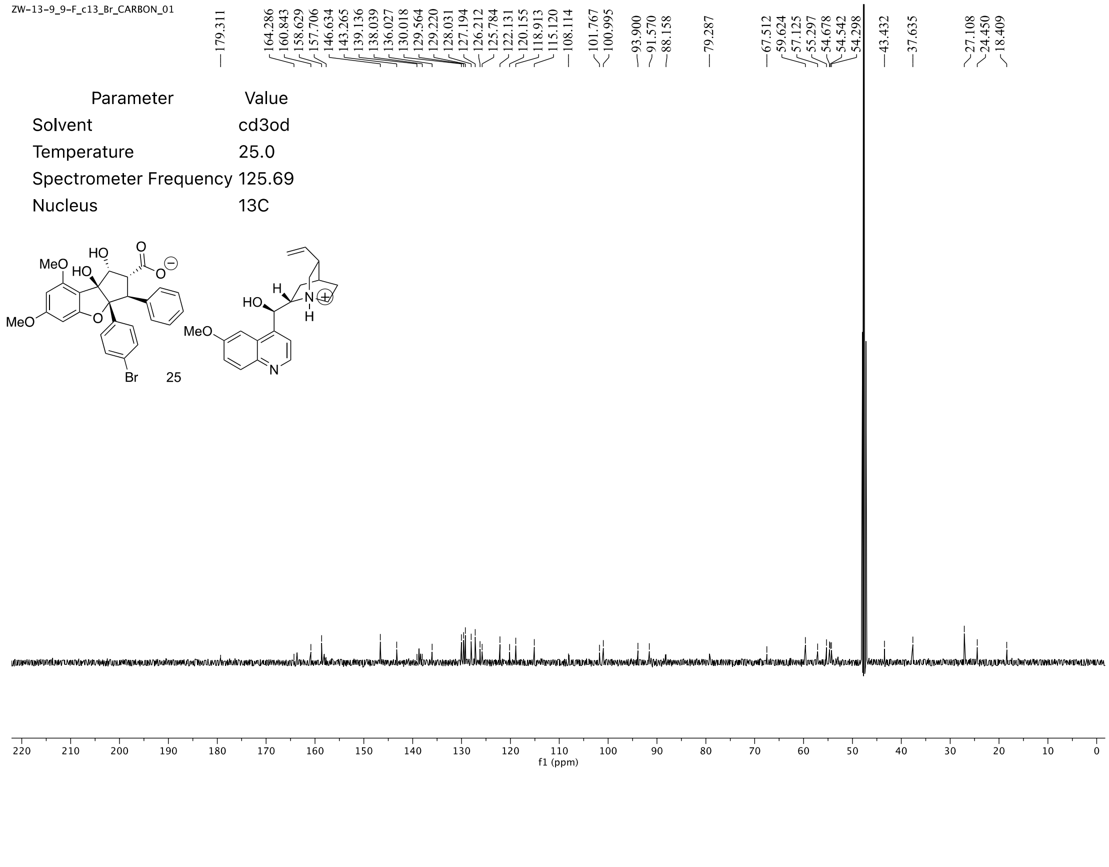
ZW-13-9_9-F_c13_Br_CARBON_01
179.311
164.286
160.843
158.629
157.706
146.634
143.265
139.136
138.039
136.027
130.018
129.564
129.220
128.031
127.194
126.212
125.784
122.131
120.155
118.913
115.120
108.114
101.767
100.995
93.900
91.570
88.158
79.287
67.512
59.624
57.125
55.297
54.678
54.542
54.298
43.432
37.635
27.108
24.450
18.409
Parameter | Value
Solvent | cd3od
Temperature | 25.0
Spectrometer Frequency | 125.69
Nucleus | 13C
25
f1 (ppm)

VII. X-ray Crystallographic Data for Compound 24

X-ray crystal diffraction data for (+)-rocaglaic acid – (-)-quinine salt **24**. Crystals of compound **24** suitable for X-ray analysis were obtained by slow evaporation from methanol.

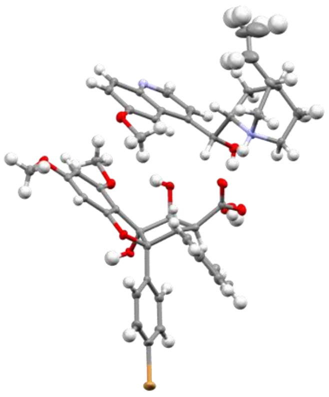

Crystal data

$C_{26}H_{22}BrO_7 \cdot C_{20}H_{25}N_2O_2 \cdot H_2O$	D_x = 1.366 Mg m^{-3}
M_r = 869.78	Cu *K*a radiation, l = 1.54184 Å
Orthorhombic, $P2_12_12_1$	Cell parameters from 46591 reflections
a = 7.0577 (1) Å	q = 2.4–75.6°
b = 18.6325 (3) Å	m = 1.83 mm^{-1}
c = 32.1571 (4) Å	*T* = 100 K
V = 4228.74 (10) Å^3	Plank, white
Z = 4	0.28 × 0.09 × 0.08 mm
F(000) = 1816	

Data collection

Saxi-CrysAlisPro-abstract goniometer imported SAXI images diffractometer	8364 independent reflections
Radiation source: fine-focus sealed X-ray tube, Enhance (Cu) X-ray Source	8251 reflections with *I* > 2s(*I*)
Graphite monochromator	R_{int} = 0.099
w and f scans	q_{max} = 75.6°, q_{min} = 2.8°
Absorption correction: multi-scan *CrysAlis PRO* 1.171.42.49 (Rigaku Oxford Diffraction, 2022) Empirical absorption correction using spherical harmonics, implemented in SCALE3 ABSPACK scaling algorithm.	*h* = -8 → 8
T_{min} = 0.816, T_{max} = 1.000	*k* = -22 → 21
140268 measured reflections	*l* = -38 → 39

Refinement

Refinement on F^2	H atoms treated by a mixture of

	independent and constrained refinement
Least-squares matrix: full	$w = 1/[s^2(F_o^2) + (0.0335P)^2 + 2.8976P]$ where $P = (F_o^2 + 2F_c^2)/3$
$R[F^2 > 2s(F^2)] = 0.035$	$(D/s)_{max} = 0.001$
$wR(F^2) = 0.084$	$D\rho_{max} = 0.62$ e Å^{-3}
$S = 1.09$	$D\rho_{min} = -0.41$ e Å^{-3}
8364 reflections	Extinction correction: *SHELXL2018*/3 (Sheldrick 2018), $Fc^*=kFc[1+0.001xFc^2l^3/sin(2q)]^{-1/4}$
570 parameters	Extinction coefficient: 0.00085 (10)
19 restraints	Absolute structure: Flack x determined using 3281 quotients [(I+)-(I-)]/[(I+)+(I-)] (Parsons, Flack and Wagner, Acta Cryst. B69 (2013) 249-259).
Primary atom site location: dual	Absolute structure (Flack) parameter: 0.002 (5)
Hydrogen site location: mixed	

VIII. Supplementary References

S1. Chen, M.; Asanuma, M.; Takahashi, M.; Shichino, Y.; Mito, M.; Fujiwara, K.; Saito, H.; Floor, S. N.; Ingolia, N. T.; Sodeoka, M.; Dodo, K.; Ito, T.; Iwasaki, S., Dual targeting of DDX3 and eIF4A by the translation inhibitor rocaglamide A. *Cell Chem. Biol.* **2021,** *28* (4), 475-486.e8.

S2. Gaetani, M.; Sabatier, P.; Saei, A. A.; Beusch, C. M.; Yang, Z.; Lundström, S. L.; Zubarev, R. A., Proteome Integral Solubility Alteration: A High-Throughput Proteomics Assay for Target Deconvolution. *J. Proteome Res.* **2019,** *18* (11), 4027-4037.

S3. Moggridge, S.; Sorensen, P. H.; Morin, G. B.; Hughes, C. S., Extending the Compatibility of the SP3 Paramagnetic Bead Processing Approach for Proteomics. *J. Proteome Res.* **2018,** *17* (4), 1730-1740.

S4. Hughes, C. S.; Moggridge, S.; Müller, T.; Sorensen, P. H.; Morin, G. B.; Krijgsveld, J., Single-pot, solid-phase-enhanced sample preparation for proteomics experiments. *Nat. Protoc.* **2019,** *14* (1), 68-85.

S5. Cox, J.; Neuhauser, N.; Michalski, A.; Scheltema, R. A.; Olsen, J. V.; Mann, M., Andromeda: A Peptide Search Engine Integrated into the MaxQuant Environment. *J. Proteome Res.* **2011,** *10* (4), 1794-1805.

S6. Cox, J.; Mann, M., MaxQuant enables high peptide identification rates, individualized p.p.b.-range mass accuracies and proteome-wide protein quantification. *Nat. Biotechnol.* **2008,** *26* (12), 1367-1372.

S7. Elias, J. E.; Gygi, S. P., Target-decoy search strategy for increased confidence in large-scale protein identifications by mass spectrometry. *Nat. Methods* **2007,** *4* (3), 207-214.

S8. Blum, B. C.; Emili, A., Omics Notebook: robust, reproducible and flexible automated multiomics exploratory analysis and reporting. *Bioinform. Adv.* **2021,** *1* (1), vbab024.

S9. Chu, J.; Zhang, W.; Cencic, R.; Devine, W. G.; Beglov, D.; Henkel, T.; Brown, L. E.; Vajda, S.; Porco, J. A.; Pelletier, J., Amidino-Rocaglates: A Potent Class of eIF4A Inhibitors. *Cell Chem. Biol.* **2019,** *26* (11), 1586-1593.e3.

S10. Novac, O.; Guenier, A. S.; Pelletier, J., Inhibitors of protein synthesis identified by a high throughput multiplexed translation screen. *Nucleic Acids Res.* **2004,** *32* (3), 902-915.

S11. Li, R.; Hu, K.; Liu, H.; Green, M. R.; Zhu, L. J., OneStopRNAseq: A Web Application for Comprehensive and Efficient Analyses of RNA-Seq Data. *Genes* **2020,** *11* (10), 1165.

S12. Dobin, A.; Davis, C. A.; Schlesinger, F.; Drenkow, J.; Zaleski, C.; Jha, S.; Batut, P.; Chaisson, M.; Gingeras, T. R., STAR: ultrafast universal RNA-seq aligner. *Bioinformatics* **2012,** *29* (1), 15-21.

S13. Harrow, J.; Frankish, A.; Gonzalez, J. M.; Tapanari, E.; Diekhans, M.; Kokocinski, F.; Aken, B. L.; Barrell, D.; Zadissa, A.; Searle, S.; Barnes, I.; Bignell, A.; Boychenko, V.; Hunt, T.; Kay, M.; Mukherjee, G.; Rajan, J.; Despacio-Reyes, G.; Saunders, G.; Steward, C.; Harte, R.; Lin, M.; Howald, C.; Tanzer, A.; Derrien, T.; Chrast, J.; Walters, N.; Balasubramanian, S.; Pei, B.; Tress, M.; Rodriguez, J. M.; Ezkurdia, I.; van Baren, J.; Brent, M.; Haussler, D.; Kellis, M.; Valencia, A.; Reymond, A.; Gerstein, M.; Guigo, R.; Hubbard, T. J., GENCODE: the reference human genome annotation for The ENCODE Project. *Genome Res.* **2012,** *22* (9), 1760-74.

S14. Liao, Y.; Smyth, G. K.; Shi, W., featureCounts: an efficient general purpose program for assigning sequence reads to genomic features. *Bioinformatics* **2013,** *30* (7), 923-930.

S15. Love, M. I.; Huber, W.; Anders, S., Moderated estimation of fold change and dispersion for RNA-seq data with DESeq2. *Genome Biol.* **2014,** *15* (12), 550.

S16. Stephens, M., False discovery rates: a new deal. *Biostatistics* **2016,** *18* (2), 275-294.

S17. Subramanian, A.; Tamayo, P.; Mootha, V. K.; Mukherjee, S.; Ebert, B. L.; Gillette, M. A.; Paulovich, A.; Pomeroy, S. L.; Golub, T. R.; Lander, E. S.; Mesirov, J. P., Gene set enrichment analysis: A knowledge-based approach for interpreting genome-wide expression profiles. *Proc. Natl. Acad. Sci. U.S.A.* **2005,** *102* (43), 15545-15550.

S18. Lajkiewicz, N. J.; Cognetta, A. B., III; Niphakis, M. J.; Cravatt, B. F.; Porco, J. A., Jr., Remodeling Natural Products: Chemistry and Serine Hydrolase Activity of a Rocaglate-Derived β-Lactone. *J. Am. Chem. Soc.* **2014,** *136* (6), 2659-2664.
